# Glucose-derived receptors for photo-controlled binding of amino acid esters in water

**DOI:** 10.1038/s42004-025-01445-x

**Published:** 2025-02-19

**Authors:** Mario M. Most, Linus B. Boll, Peter Gödtel, Zbigniew L. Pianowski, Bartosz Lewandowski

**Affiliations:** 1 Institute of Organic Chemistry, Karlsruhe Institute of Technology (KIT), Kaiserstrasse 12, 76131 Karlsruhe, Germany; 2 Laboratory of Organic Chemistry, ETH Zürich, Vladimir-Prelog-Weg 3, 8093 Zürich, Switzerland; 3 Institute of Biological and Chemical Systems - Functional Molecular Systems IBCS-FMS, Karlsruhe Institute of Technology (KIT), Kaiserstrasse 12, 76131 Karlsruhe, Germany

**Keywords:** Molecular capsules, Self-assembly, Bioanalytical chemistry

## Abstract

Selective receptors of amino acids in aqueous media are highly sought after as they may enable the creation of novel diagnostic and sensing tools. Photoswitchable receptors are particularly attractive for such purposes as their response and selectivity towards bioanalytes can be modulated using light. Herein we report glucose-based photoswitchable receptors of amino-acid methyl esters and biogenic amines in water. The tetra-*ortho*-fluoroazobenzene unit in the receptors structure allows to control the distance between their binding sites using light. The *Z*-isomers of both receptors, having these sites in closer proximity, bind lysine, ornithine and arginine esters significantly stronger compared to *E*-isomers, where the binding sites are further apart.

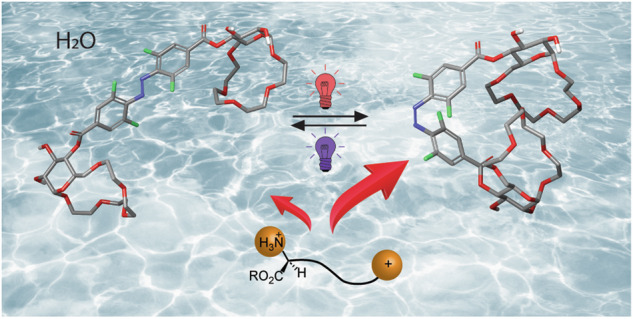

## Abstract (web summary)

Selective amino acid receptors that operate in aqueous media are of interest for potential diagnostic and sensing applications. Here, the authors report glucose-derived photoswitchable receptors based on a tetra-*ortho*-fluoroazobenzene unit for light-controlled binding of amino acid esters in water.

## Introduction

**Fig. 1:**
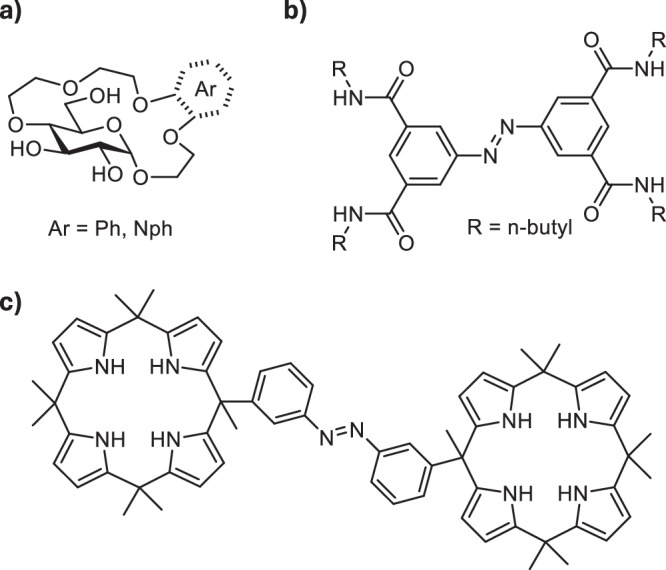
Synthetic ion receptors. **a** Glucose-based aryl crown ethers for enantio- and chemoselective binding of amino acid esters in water (refs. ^20,21^); (**b**) photoswitchable receptor of inorganic anions (ref. ^37^); (**c**) photoswitchable receptor of organic dianions (ref. ^49^).

Amino acids play key roles in numerous biological processes^1^, e.g. tryptophan is involved in electron transport in proteins^2^, aspartic acid functions as a neurotransmitter^3^, lysine is important for histone modifications^4^. Amino acids have also found applications in asymmetric synthesis and catalysis^5,6^, chemical biology^7–9^ and materials science^10–12^. Chemo- and enantioselective binding of amino acids is therefore important for diagnostic, medicinal and technological purposes^13–15^. From biological standpoint, amino acid recognition in water is particularly important^15^. However, it is also challenging since it requires synthetic receptors which combine polar interactions and hydrophobic effect to achieve strong and selective amino acid binding in aqueous media^16–18^. Despite the progress in development of synthetic amino acid receptors, ones that bind chemo- and enantioselectively in water are rare^15,19^. Recently, we have reported glucose-derived crown and benzocrown ethers capable of enantioselective binding of amino acid esters with hydrophobic and aromatic side chains in water (Fig. 1a)^20,21^. We also established a naphthocrown receptor chemoselective towards aromatic amino acids (Fig. 1a)^21^.

Receptors which can alter their affinity towards target analytes in response to external stimulus are highly sought after, as they can find application for controlled binding, transport and release of guest molecules^22,23^. Among the stimuli used to regulate the strength of guest binding, light is particularly advantageous as it can be applied remotely, with spatiotemporal control and in a waste-free manner^24,25^. In order to create light-responsive receptors, photoswitchable motifs are introduced into their structure. Molecular photoswitches^26^ are compounds that serve as molecular light adapters—they undergo reversible photoisomerization, which causes modulation in their geometry, polarity, stiffness, and other parameters. A number of photoswitches can be used in aqueous environments, which is important for applications in biological context^27^. Their biocompatibility can also be enhanced by using systems operational entirely with visible light frequencies^28,29^, as UV light used in majority of photochromic setups may be cytotoxic and poorly penetrates living organisms. The established photoswitches, such as azobenzenes^30^, spiropyrans^31^, or indigoids^32^, have recently been complemented with novel scaffolds^33^, including iminothioindoxyls^34^, or hemipiperazines^35^. The different classes of photoswitches have been applied in light-controlled receptors for inorganic (Fig. 1b)^36–41^, or organic ions^42,43^, and for neutral guests^44^ as well as ligands for photomodulated binding to cyclodextrins^45^ or cucurbiturils^46^. However the majority of photo-responsive host-guest systems utlize azobenzenes^47^. Azobenzenes have also been used to create homoditopic receptors for light-controlled assembly with diammonium cations^48^ and the binding of dicarboxylate anions (Fig. 1c)^49^ in organic solvents.

Herein we report photoswitchable glucose derived receptors for light-controlled binding of amino acid methyl esters in water. We show that we can use light to modulate the binding affinity of the receptors to amino acids with cationic sidechains (Lys, Arg, Orn) and achieve stronger binding using the *Z*-isomers. Additionally, we observe a preference for the binding of *l*- enantiomers of the dicationic amino acid esters by *Z*-configured receptors.

## Results and discussion

**Fig. 2:**
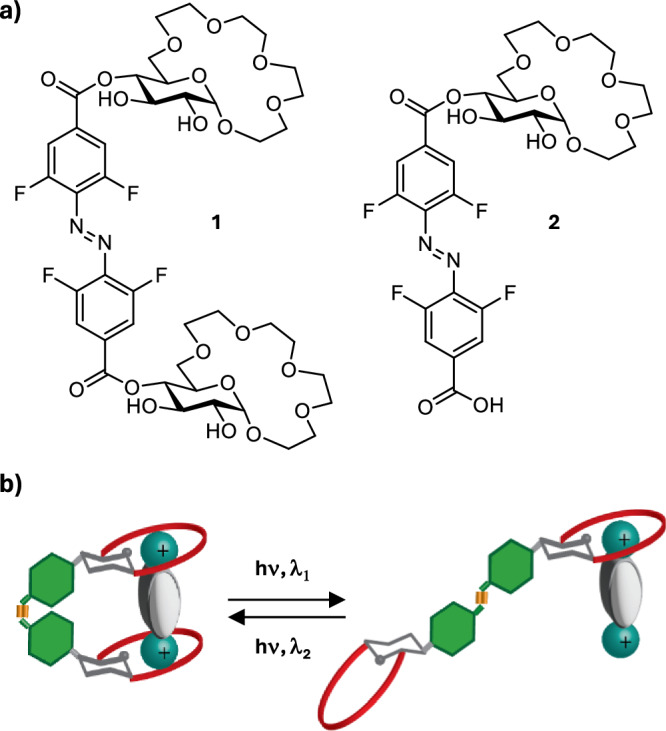
Design of the photoswitchable receptors. **a** Structures of glucose-derived photoswitchable receptors of amino acid esters **1** and **2**; (**b**) schematic representation of the light-controlled binding of dicationic guests by the receptors.

To access photoswitchable amino acid receptors, we decided to combine a glucose-derived crown ether analogous to the one previously reported by Lewandowski et al. with a photoresponsive tetrafluoroazobenzene unit (Fig. 2a). Ortho-fluorinated azobenzenes^50^ display an appealing blend of high thermal and biological stability, good bidirectional photoconversions, and the response to visible light. They’ve recently been applied in photo-switchable transmembrane transporters of halide anions^51,52^. Pianowski et al. used a *ortho*-fluoroazobenzene-derived chromophore to prepare reversibly photodissipated supramolecular hydrogels with potential applicability in light-driven drug release^53,54^. We thus envisioned that the favourable photophysical properties of ortho-fluorinated azobenzenes make them highly suitable for the construction of our target sensors.

We prepared two receptors: **1**- containing two glucose crown ethers conjugated to 4,4’-tetrafluoroazobenzene dicarboxylic acid *via* the hydroxy group in position 4 of the pyranose ring, and **2** - where only one glucose crown ether was attached to the tetrafluoroazobenzene unit (Fig. 2a). We anticipated that having the photoswitch as the *Z* isomer would bring the two binding motifs of the receptor (two glucose crown ethers in the case of **1** or one glucose crown ether and the carboxylic acid/carboxylate in the case of **2**) in closer proximity allowing to bind dicationic guests with higher affinity (Fig. 2b). With the photoswitch in the *E* configuration, the two binding sites of the receptor would be too far apart to simultaneously bind the guest molecule, thus resulting in a reduced binding affinity (Fig. 2b).

**Scheme 1:**
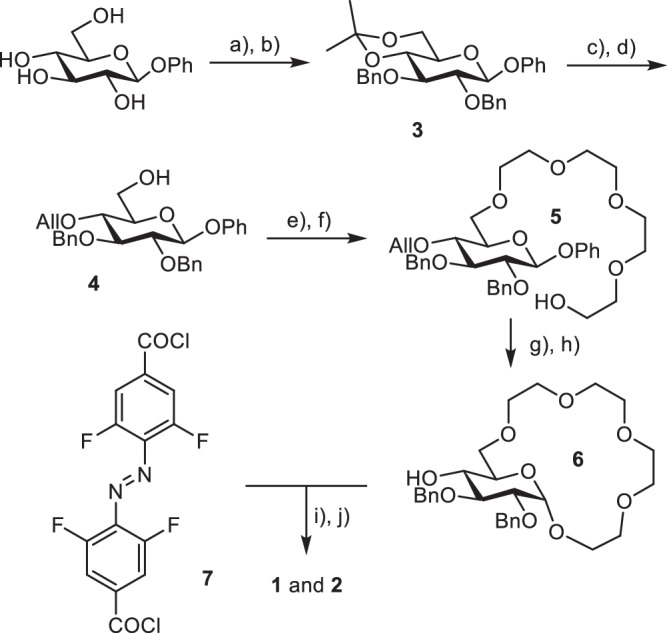
**Synthesis of receptors 1 and 2**. Reagents and conditions: (**a**) DMP, p-TsOH, acetone, r.t., 24 h, 85%; (**b**) BnBr, NaH, DMF, r.t., 6 h, 90%; (**c**) AcOH/H_2_O = 3:2, 50 °C, 2 h, 95%; (**d**) AllBr (1 eqv.), NaH, DMF, r.t., 8 h, 40%; (**e**) Bis-chloro-diethylether, TBAI, 50%aq KOH, THF, 50 °C, 12 h, 90%; (**f**) KOH, diethylene glycol, 80 °C, 8 h, 85%; (**g**) FeCl_3_, NH_4_PF_6_, CH_3_CN, 40 °C, 48 h, 65%; (**h**) Pd/C, p-TsOH, MeOH/H_2_O = 4:1, reflux, 5 h, 90%; (**i**) **7** (0.75 eqv.), NEt_3_, CH_2_Cl_2_, r.t., 24 h; (**j**) NaBrO_3_, Na_2_S_2_O_4_, ethyl acetate/H_2_O, r.t., 72 h, **1**–30%; **2**–40% over two steps.

The receptors were synthesized starting from β-glucopyranoside and 4,4’-tetrafluoroazobenzene dicarboxylic acid (Scheme 1). Selective protection of the hydroxy groups in positions 4 and 6 of the glucopyranoside using dimethoxypropane, followed by alkylation of the 2- and 3-OH with benzyl bromide yielded the fully protected derivative **3**. Removal of the acetal protecting group followed by alkylation using allyl bromide provided **4**. The crown ether fragment was formed by analogous strategy to the one previously developed by us^20^ involving alkylation with bis-chloroethyl ether, elongation of the oligoethylene glycol fragment (yielding **5**) and subsequent macrocyclization using FeCl_3_ to activate the glycoside for reaction. Removal of allyl protecting group provided alcohol **6** which was then coupled to the tetra-*ortho-*fluoroazobenzene dicarboxylic acid^55^ activated as its diacyl chloride **7**. Removal of Bn protecting group in oxidative conditions yielded the target receptors **1** and **2** (for further experimental details of the synthesis see Supplementary Note 1).

**Fig. 3:**
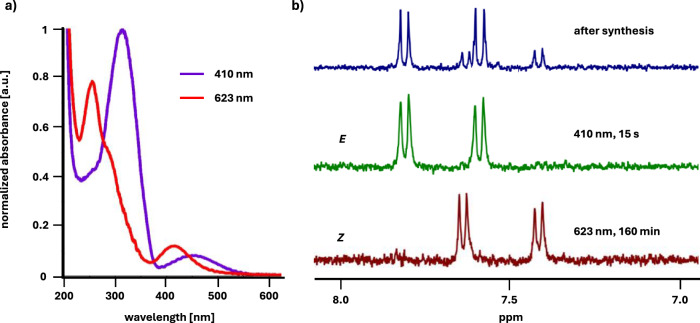
Photochromic characterization of receptors. (**a**) UV-VIS spectra of **2** in H_2_O (c = 100 µM); (**b**) fragments of ^1^H NMR spectra of **2** in D_2_O showing the signals of aromatic protons: directly after the synthesis of the receptor (top), after 15 s irradiation with 410 nm light (middle), after 160 min irradiation with 623 nm light (bottom).

With the receptors in hand, we proceeded to study their photochromic properties. First, we established the time required to reach the photostationary states (PSS) and the ratio of the *E* to *Z* isomers at PSS for both receptors. To achieve the former goal, we irradiated the samples of the receptors in H_2_O using light of two different wavelengths λ = 410 nm for *Z* to *E* isomerization and λ = 623 nm for *E* to *Z* isomerization. We monitored the changes of their UV-Vis absorption spectra over time until no further changes were observed, indicating that the photostationary state was reached (Fig. 3a, Figs. S1, S2). To determine the *E*/*Z* isomer ratio at PSS we used ^1^H NMR spectroscopy. The aromatic proton peaks corresponding to each isomer have distinct chemical shifts due to different geometry of the azobenzene unit. Thus, by irradiating the samples of both receptors in D_2_O for the previously determined period of time required to reach the PSS, subsequent ^1^H NMR analyses (Figs. 3b and S3) and integration of the aromatic proton signals in the spectra, the ratio of *E* to *Z* isomer at PSS was established. Pleasingly, both receptors undergo efficient photoisomerization with red light (623 nm) resulting in almost exclusive (90% for **1** and 95% for **2**, Table S1) formation of the *Z* isomer after 160 minutes. At that frequency, the ε of both isomers are low, but the favourable ε_E_/ε_Z_ ratio enables almost quantitative *E* to *Z* photoconversion. Irradiation with violet light (410 nm) over 15 seconds, on the other hand, allows to switch the receptor completely to the *E* isomer (> 95% for both **1** and **2**, Table S1). Additionally, we determined the quantum yields of the photoisomerization of receptor **1** in both directions: *Φ*_*E*→*Z*_ = 0.09 ± 0.004 and *Φ*_Z→E_ = 0.6 ± 0.031 (Figs. S5, S6)^56^. These values are very similar to those previously obtained for the diethyl ester of 4,4’-tetrafluoroazobenzene dicarboxylic acid^50^. Fatigue resistance measurements revealed that the receptor is stable over at least 20 photoswitching cycles (Fig. S7)^56^.

Having demonstrated that we can access the two photoisomers of receptors **1** and **2** with high selectivity, we next studied their binding affinities for amino acid methyl esters in water. In particular, we wanted to investigate whether changing the geometry of the azobenzene unit in the receptors (*Z* vs *E* isomer) affects their binding strength and selectivity. We primarily chose to study amino acids with cationic side chains—Lys, Orn and Arg, as we anticipated that the presence of two binding sites in **1** and **2** should be beneficial for the binding of dicationic guests. We also studied Phe and Val, with aromatic and hydrophobic side chains respectively, which were among the guests bound with the highest affinities by our previously reported glucose crown and aryl crown ethers^20,21^. Finally, we used Ala methyl ester to verify the importance of the side chain functionality in the amino acid for its binding by **1** and **2**. Aside from amino acids we also studied three examples of biologically relevant di- and oligoamines (cadaverine, spermidine and spermine) as guests. Guest binding was studied using Isothermal Titration Calorimetry (ITC) in H_2_O. The results are summarized in Table 1 (for details of the ITC experiments see Supplementary Note 7).

**Table 1:** Binding affinities of receptors **1** and **2** to amino-acid methyl ester hydrochlorides, di- and oligoamines determined by ITC experiments in H_2_O

Guest^a^	Receptor 1			Receptor 2		
	K_a_*Z*^b^	K_a_*E*	Inc.^c^	K_a_*Z*	K_a_*E*	Inc.
*l*-Lys	**1408** ± **377**	**463** ± **124**	**3.0**	**1251 ± 438**	**523 ± 129**	**2.4**
*l*-Arg	**1369 ± 225**	**551 ± 124**	**2.5**	**1176 ± 522**	**488 ± 165**	**2.4**
*l*-Orn	**1562** ± **171**	**549** ± **75**	**2.8**	**1409** ± **483**	**529** ± **45**	**2.7**
*l*-Phe	1064 ± 294	833 ± 250	1.3	794 ± 283	826 ± 280	1.0
*l*-Val	775 ± 312	675 ± 223	1.1	617 ± 209	662 ± 258	0.9
*l*-Ala	546 ± 187	490 ± 1771	1.1	543 ± 242	476 ± 166	1.1
*d*-Lys	729 ± 266	367 ± 155	2.0	746 ± 250	418 ± 115	1.8
*d*-Arg	709 ± 191	492 ± 198	1.4	781 ± 299	392 ± 125	2.0
*cadaverine*	**1655** ± **449**	**606** ± **189**	**2.7**	**1172** ± **312**	**500** ± **133**	**2.3**
*spermidine*	1430 ± 117	1794 ± 223	0.8	1490 ± 154	1825 ± 209	0.8
*spermine*	1752 ± 244	2211 ± 309	0.8	1818 ± 175	2163 ± 202	0.8

^a^H-AA-OMe x HCl were used as guests; ^b^K_a_ values are reported in M^–1^; ^c^K_a_ increase for *Z*- vs *E*-receptor molecule; Bold font indicates the examples where highest binding selectivity in favour of the *Z*-isomer was observed.

The *Z* isomer of receptor **1**, with two glucose crown ethers, displayed the highest binding affinities towards amino acids with cationic side chains (Lys, Orn, Arg). These affinities are also among the highest observed for glucose-derived crown ether type receptors to date. The *E* isomer of **1** bound these three amino acids considerably (2.5-3x) weaker (Table 1, entries 1–3, columns 2–4). The binding affinities for Val, Phe and Ala were similar for both isomers of receptor **1** and close to those displayed by the previously reported glucose benzo crown ether (Table 1, entries 4–6, columns 2–4)^21^. In the case of receptor **2**, with one glucose crown ether and a carboxylic acid, the affinity of the *Z*-isomer for Lys, Orn and Arg was slightly lower than that displayed by **1** which also resulted in inferior selectivity of binding (2.4-2.7x) between the *Z* and *E* isomer of the receptor (Table 1, entries 1–3, columns 5–7). The affinities of **2** for Val, Phe and Ala were almost identical to those displayed by **1** (Table 1, entries 4–6, columns 5–7). To verify whether the chiral glucose unit(s) in the receptors allow to achieve enantioselective guest binding we also determined the affinities of **1** and **2** to H-*d*-Lys-OMe and H-*d*-Arg-OMe. The *Z* isomers of both receptors bound *l*-enantiomers of Lys and Arg around 2x stronger than *d*-enantiomers (Table 1, entries 7–8, columns 2 and 5), whereas the *E* isomer showed almost no enantioselectivity (Table 1, entries 7–8, columns 3 and 6). Both receptors displayed similar binding affinities and selectivities for the binding of cadaverine as those observed for *l*-Lys (Table 1, entry 9, columns 2–7). Interestingly, in the case of spermidine and spermine the *E* isomers of **1** and **2** displayed slightly higher binding affinities than the *Z* isomers for which the binding affinities were comparable, albeit marginally larger than those observed for Lys and Orn binding (Table 1, entries 10 and 11, columns 2–7). The fitting of the data obtained in the ITC experiments indicated a 1:1 stoichiometry of the complexes for both isomers of the receptors with all studied guests (applications of other models, e.g. 1:2 did not provide a fit).

Having obtained promising results in the ITC experiments carried out in water we next tested whether the two receptors can also be used for the light-controlled binding of dicationic guests in a more complex and challenging environment of an aqueous buffer. The binding of **1** and **2** to Lys, Arg and Orn methyl esters in PBS buffer (pH 7.4) was also studied by ITC and the results are summarized in Table 2.

**Table 2:** Binding affinities of receptors **1** and **2** to Lys, Arg and Orn methyl ester hydrochlorides determined by ITC experiments in PBS buffer (pH 7.4)

Guest^a^	Receptor 1			Receptor 2		
	K_a_*Z*^b^	K_a_*E*	Inc.^c^	K_a_*Z*	K_a_*E*	Inc.
*l*-Lys	**758** ± **313**	**188** ± **98**	**4.0**	792 ± 305	252 ± 114	3.1
*l*-Arg	**782 ± 207**	**221 ± 115**	**3.5**	704 ± 274	257 ± 108	2.7
*l*-Orn	**621** ± **213**	**176** ± **69**	**3.5**	**839** ± **266**	**260** ± **126**	**3.2**

^a^H-AA-OMe x HCl were used as guests; ^b^K_a_ values are reported in M^-1^; ^c^K_a_ increase for *Z*- vs *E*-receptor molecule; Bold font indicates the examples where highest binding selectivity in favour of the *Z* isomer was observed.

The *Z* isomers of the receptors bound the amino acid guests in PBS buffer approximately two times weaker compared to water (Table 2, column 2 and 5). On the other hand, the *E* isomers of **1** and **2** displayed more than two-fold decrease in the binding affinity (Table 2, column 3 and 6). As a consequence, the selectivity of guest binding by *Z*-receptors increased in PBS with respect to water. This was particularly pronounced for ***Z*-1** which bound *l*-Lys 4x stronger and *l*-Arg and *l*-Orn 3.5x stronger than ***E*-1**.

**Fig. 4:**
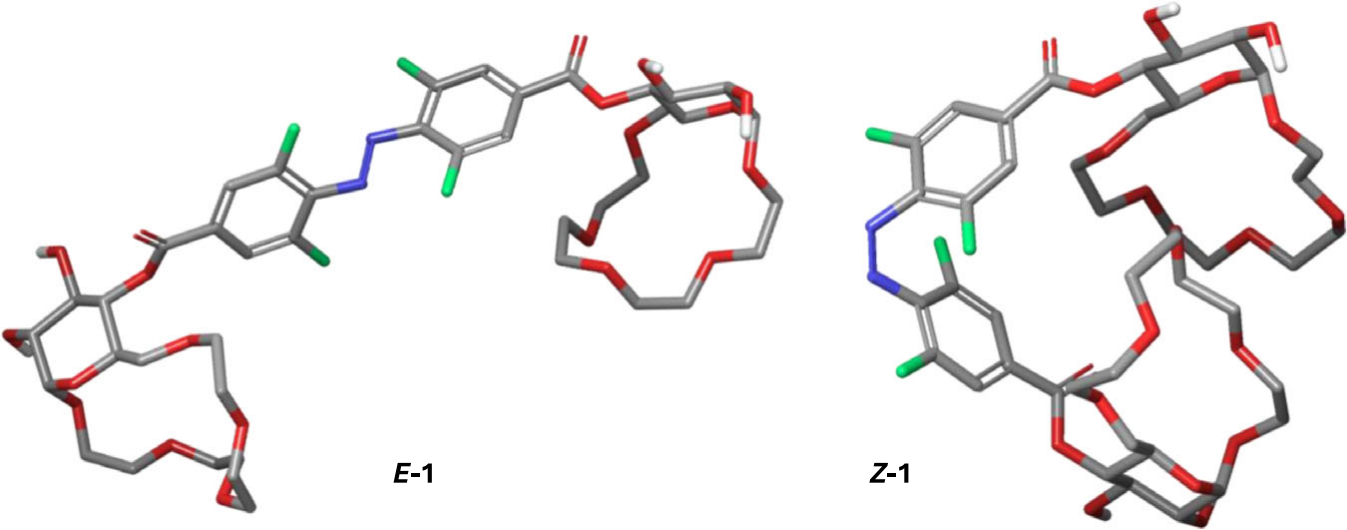
Computational modelling of receptor 1. Lowest energy conformers of *Z*- and *E*-isomers of receptor **1** at the r^2^SCAN-3c(CPCM(water))+GFN2-xTB(ALPB(water)) level.

The ITC experiments demonstrated that changing the configuration of the azobenzene unit in receptors **1** and **2** allows to control their affinity to amino acid esters with cationic side chains as well as to aliphatic diamines in water and PBS buffer. The *Z* isomer binds these guests significantly stronger likely due to proximity of the two binding sites in the receptor which can both engage in guest coordination. The distance between the binding sites in the *E* isomer is too high to allow both to bind the guest molecule. To support this hypothesis, we performed computational analysis on both the *Z*- and the *E*-configured receptors. Conformational searches using CREST^57,58^ followed by reranking using CENSO^59^ provided conformer-rotamer ensembles at the r^2^SCAN-3c(CPCM(water))+GFN2-xTB(ALPB(water)) level. Analysis of the ensembles revealed that in the *E* isomer of receptor **1**, the two glucose crown ether units are in a 15 Å distance from one other (Fig. 4, left). On the other hand, in *Z*-**1**, the two glucose crown ethers form a highly defined cavity around the azobenzene unit with a much shorter distance of 6 Å between the two macrocycles (Fig. 4, right).

**Fig. 5:**
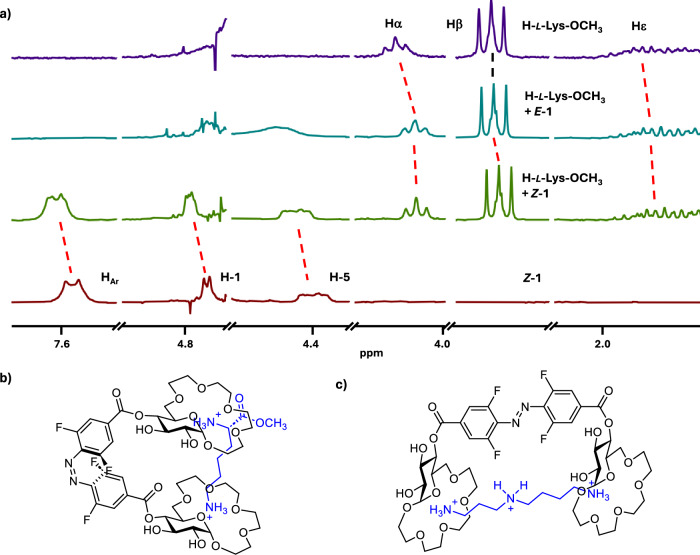
Binding mode analysis. (**a**) Stacked plot of ^1^H NMR spectra (D_2_O, 400 MHz, c = 2 mM, only selected regions are shown)—from the top: H-*l*-Lys-OCH_3_, H-*l*-Lys-OCH_3_ + *E*-**1** (1 equiv.), H-*l*-Lys-OCH_3_ + *Z*-**1** (1 equiv.), *Z*-**1**; (**b**) proposed binding mode of H-*l*-Lys-OCH_3_ by *Z*-**1**; (**c**) proposed binding mode of spermidine by *E*-**1**.

To gain insight into the binding mode of the dicationic guests by receptors **1** and **2** we performed NMR analyses of the host/guest complexes in D_2_O. We analysed the complexes of **1** with *l*-Lys and **2** with *l*-Orn – which appeared to be more stable complexes than the ones with Arg as guest. Upon binding of *l*-Lys by *E*-**1**, a considerable upfield shift of the α protons and a slightly smaller shift of the β protons of the guest was observed (Fig. 5a). The signals corresponding to other side-chain protons as well as those of the methyl ester only shifted marginally or not at all. At the same time a noticeable downfield shift of H-1, H-5 and of some of the oligoethylene glycol protons as well as the aromatic, azobenzene protons in the receptor was observed (Fig. S8)^56^. These observations suggest that the *E*-**1** binds H-*l*-Lys-OMe primarily through Coulombic interactions of one of the crown ether units with the α ammonium cation in Lys. Additionally, cation-π interactions between the ε ammonium and the aromatic azobenzene could also be involved. In the case of the H-*l*-Lys-OMe@*Z*-**1** complex significant upfield shift of the Lys H_ε_ compared to the complex with *E*-**1** was observed. H_α_ in Lys also shifted upfield compared to the complex with *E*-**1** (Fig. 5a). Also, further downfield shifts of the H-1 glucopyranose protons and those of the oligoethylene glycol units in the receptor, were observed in the H-*l*-Lys-OMe@*Z*-**1** complex. This indicates that the *Z*-isomer binds *l*-Lys-OMe through interactions of the two crown ether units with the two ammonium groups in the guest (Fig. 5b) resulting in a more stable complex with dicationic H-*l*-Lys-OMe than that formed by *E*-**1**. The proposed binding mode was also supported by computational analyses of the complexes (Fig. S13)^56^. NMR analysis of the complexes formed by *E*- and *Z*-**2** with *l*-Orn-OMe suggested a similar binding mode (Fig. S9)^56^. Here, however, the *Z* isomer of **2** binds the second ammonium group of the guest through an electrostatic interaction with the carboxylate of the receptor. A similar binding mode is also plausible for the complexes of both receptors with cadaverine and consequently similar binding affinities and selectivities are observed. In the case of spermidine and spermine the presence of additional amino groups at increased distances from one another compared to cadaverine results in higher stability of the complexes of these amines with the *E* isomers of **1** and **2**, which have their binding sites further apart than the *Z* isomers (Fig. 5c). Of note, the binding of dicationic guests does not alter the photoswitching of the receptors. UV-Vis measurements performed on a 1:1 complex of receptor **1** and H-*l*-Lys-OMe revealed that back-switching performance is not altered, indicated by virtually complete reversion to the initial spectrum of the dark state and an unaffected reaction quantum yield (Figs. S5, S6)^56^. NMR analyses of the complexes of **1** with H-*l*-Lys-OMe as well as **2** with H-*l*-Orn-OMe showed that the *E*/*Z* isomer ratio at PSS for both receptors is the same as in the uncomplexed form (Figs. S8, S9)^56^.

With regards to the other amino acid guests used in this study—Phe, Val, Ala - the binding by receptors **1** and **2** likely involves a combination of Coulombic interactions between the crown ether unit in the host and ammonium in the guest and π-π or CH-π interactions between the azobenzene group in the receptors and the amino acid side chains. This binding mode is also supported by NMR analyses of the complexes between *E*- and *Z*-**2** and H-*l*-Phe-OMe (Fig. S10)^56^. Since all these interactions can be formed by both isomers of the receptor, they show similar affinities to these three guests.

## Conclusions

In conclusion we have designed and synthesized two photoswitchable receptors for the binding of amino acid esters in water and buffer. The receptors contain one or two glucose crown ethers connected to a tetra-*ortho*-fluoroazobenzene photoresponsive unit. Both receptors show attractive photochromic properties as the *Z* and *E* isomers of each receptor are thermally stable, and thus accessible almost quantitatively by irradiation with red or violet light, respectively. We have shown that the binding affinity of the receptors to amino acid methyl esters with cationic side chains can be modulated by light-triggered isomerization of the azobenzene units. The *Z* isomers of both receptors display enhanced binding affinities to dicationic amino acid guests compared to the *E* isomers. Furthermore, *Z*-configured receptors preferably bind *l*- enantiomers of lysine and arginine esters. DFT calculations and NMR spectroscopic analyses provided insights into the binding mode of amino acid esters by the receptors. These studies indicate that the *Z* isomers of the receptors utilize two binding sites for coordination of dicationic guests, whereas the distance between these two sites in *E* configured receptors is too large for both of them to participate in the binding of guests. In the future, we aim to develop photoswitchable receptors with further improved binding affinities and selectivities. This could enable applications in bioanalyte sensing and for controlled binding and delivery of biomolecules.

## Methods

### Materials

Solvents and reagents were of the highest commercially available grade and used without further purification. They were purchased from Sigma Aldrich, Fischer Scientific, Fluka, Bachem, BioMatrix, Biotage, IRIS Biotech, Protein Technologies and Acros Organics. Solvents used for MPLC were HPLC-grade quality.

### Preparative medium pressure liquid chromatography (MPLC)

Purifications of the building blocks were carried out on a CombiFlash EZ Prep flash chromatography system (Teledyne ISCO). Two different solvent sets were used: 1. Solvent A was HPLC-grade DCM without stabilizer and solvent B was HPLC-grade methanol. 2. Solvent A was HPLC-grade hexane and solvent B HPLC-grade EtOAc.

### Thin-layer chromatography (TLC)

TLC was conducted on aluminium sheets coated with silica gel 60 F_254_ (Merck) using UV fluorescence (254 and 366 nm). Analytical grade solvents were used.

### Liquid chromatography—mass spectrometry (LC—MS)

Analytical reverse phase HPLC (RP-HPLC) was performed on a Dionex UHPLC, Ultimate 3000. Reprosil gold 120 C_18_ (150 × 4 mm, 5 µm) with a flow of 0.5 mL/min was used as the analytical column. Two different solvents were used. Solvent A was assigned to be pure acetonitrile and solvent B was a mixture of 1% acetonitrile and 0.1% TFA in Milli-Q pure water. The mass analysis was performed on an amaZone speed ion trap mass analyzer (Bruker, USA).

### Nuclear magnetic resonance (NMR) spectroscopy

1D and 2D NMR spectra were recorded on 400, 500 and 600 MHz Ultrashield spectrometers (Bruker, USA). ^1^H-NMR chemical shifts (δ_H_) are quoted in parts per million (ppm) and coupling constants (J) are quoted in Hertz (Hz). Abbreviations for NMR data are s (singlet), d (doublet), t (triplet), q (quartet), m (multiplet).

### High-resolution mass spectrometry (HR-MS)

High-resolution electrospray ionization (HR-ESI) spectra were measured on a Bruker maXis spectrometer.

### UV-Vis spectroscopy

UV-Vis measurements were performed in 1 mL quartz cuvettes (pathway length = 1 cm) in Milli-Q pure H_2_O at rt using an Agilent Cary 300 spectrometer. The concentration of all solutions was 100 μM. The absorbance was measured from 200 to 800 nm.

### Isothermal titration calorimetry (ITC)

ITC experiments were carried out in a MicroCal PEAQ-ITC (Malvern), at 25 °C, 750 rpm, high feedback and 10 µcal/s as reference power, using MilliQ water as a solvent. The concentrations of the host and the guest solutions were 0.2 mM and 6 mM respectively. The guest solution was added in 1 µL portions at 2 min intervals. The data was analysed by fitting to a one set of sites model using the MicroCal PEAQ-ITC Control Software.

### Sample irradiation

All experiments with photochromic molecules were performed in absence of sunlight (brown glassware, or colourless glassware wrapped with aluminium foil, working in a room with dimmed light).

Sample irradiation was performed using sets of two 10 W LED diodes 410 nm, and four 10 W LED diodes 623 nm, supplied by LED Engin or a 523 nm LED diode supplied by Mouser (LZ4-40G108). For the time of irradiation, samples were maintained at constant temperature (22 ± 2 °C) using a metal cooling block. Irradiation intensities of the respective LEDs were determined using the PowerMax USB (type PS19Q) sensor device (Coherent®) in five independent measurements. The detector (diameter 19 mm) was located at a distance of 55 mm from the light source, identical to the position of irradiated samples.

The emission parameters of LEDs applied to irradiate the photoswitchable receptor were characterized using the PowerMax USB (type PS19Q) sensor device (Coherent®):

**Table:** 

λ_max_ of the LED diode	Light intensity (mW cm^−2^)	Average power (W)	Variance (W)	SD (W)
410 nm	9.07	2.57*10^−2^	2.72*10^−7^	5.52*10^−4^
523 nm	7.08	2.01*10^−2^	2.32*10^−7^	5.01*10^−4^
623 nm	27.2	7.71*10^−2^	2.05*10^−5^	4.53*10^−3^

For details on specific experiments please see Supplementary Notes 2–4 in the Supplementary Information.

### Computational modelling

3D structures of the *Z*- and *E*-configured receptor were preoptimized using GFN2-xTB in the gas-phase employing xTB version 6.6.0^60,61^. Conformer-rotamer ensemble sampling at the GFN2-xTB(ALPB(water)) level was performed using CREST version 2.12 through the interface of CREST with xTB^57,58,60–62^. Refinement of the conformer-rotamer ensembles was performed by re-ranking to the r^2^SCAN-3c(CPCM(water)) level with calculation of thermostatistical contributions using GFN2-xTB(ALPB(water))^60–65^. For the re-ranking, CENSO version 1.2.0 (Part0-2) was used through the interface of CENSO with xTB and ORCA version 5.0.3^59,66^. For further details see Supplementary note 6. The conformer-rotamer ensemble sorting settings employed with CENSO, cartesian coordinates of and information on the ensembles are provided as a separate file (Supplementary Data 2).

## Supplementary information

Supplementary Information

Description of Additional Supplementary Files

Supplementary Data 1

Supplementary Data 2

## Data Availability

Authors can confirm that all relevant data are included in the manuscript and its supplementary information files: Supplementary Information—Supplementary Figs. for the manuscript, synthetic protocols and compound characterization, UV-Vis, NMR and ITC data. Supplementary Data 1 – NMR spectra of all synthesized compounds. Supplementary Data 2 – Additional computational modelling data: conformer-rotamer ensemble sorting settings, cartesian coordinates of and information on the ensembles.
