# Supplementary material for: Glucose-derived receptors for photo-controlled binding of amino acid esters in water
Source: Commun Chem. 2025 Feb 19;8:50. doi: 10.1038/s42004-025-01445-x (PMC11840139; doi:10.1038/s42004-025-01445-x)
Supplement: Supplementary file 1 — Supplementary Information [file 42004_2025_1445_MOESM1_ESM.pdf]

Supporting Information

Index

1. **Synthesis of the receptors.** **3**
2. **Photostationary states determination.** **12**
   2.1 UV-Vis measurements. **12**
   2.2 NMR measurements. **14**
3. **Photoisomerization quantum yield measurements.** **15**
4. **Fatigue resistance measurements.** **17**
5. **Binding mode analysis – NMR experiments.** **18**
6. **Computational modelling.** **21**
7. **ITC measurements.** **23**
8. **Supplementary references** **39**

Supplementary Note 1.

Synthesis of the receptors.

4,6-*O*-isopropylidene-phenyl-β-*D*-glucopyranoside:

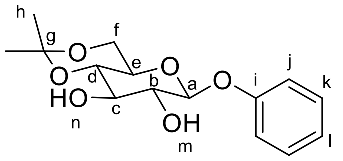

Phenyl β-D-glucopyranoside (5 g, 19.51 mmol), 2,2-dimethoxypropane (3.75 mL, 30.61 mmol) and *p*-toluenesulphonic acid monohydrate (200 mg, 1.05 mmol) were suspended in a mixture of acetone (60 mL) and DMF (3 mL). The reaction was stirred at room temperature for 72 hours. Et_3N (1.5 mL) was then added and the reaction mixture was filtered through celite. The filtrate was concentrated under reduced pressure and purified by MPLC (hexane : ethyl acetate = 100 : 0 (2 min) - 30 : 70 (over 17 min) - 0 : 100 (over 1 min)) to yield the product as a colourless oil (4.9 g, 16.5 mmol, 85% yield).

^{1}H NMR (400 MHz, DMSO-d$_6$) δ 7.34 – 7.24 (m, 2H, H_k), 7.06 – 6.95 (m, 3H, H_j + H_l), 5.56 (d, *J* = 5.3 Hz, 1H, H_n), 5.30 (d, *J* = 5.1 Hz, 1H, H_m), 5.04 (d, *J* = 7.5 Hz, 1H, H_a), 3.80 (dd, *J* = 10.5, 4.8 Hz, 1H, H_f), 3.67 (t, *J* = 10.1 Hz, 1H, H_f), 3.51 – 3.28 (m, 4H, H_b + H_c + H_d + H_e), 1.44 (s, 3H, H_h), 1.33 (s, 3H, $H_{h'}$).

^{13}C NMR (101 MHz, DMSO-d$_6$) δ 157.1 (C_i), 129.5 (C_k), 122.0 (C_l), 116.3 (C_j), 100.5 (C_a), 98.8 (C_g), 74.2 (C_b), 73.2, 73.1 (2 x C, C_c + C_d), 66.7 (C_e), 61.5 (C_f), 29.1 (C_h), 19.1 ($C_{h'}$).

HR-ESI-MS: m/z: 319.1150 $[M+Na]^+$, calculated for $C_{15}H_{20}NaO_6^+$: 319.1152

4,6-*O*-isopropylidine-2,3-di-*O*-benzyl-phenyl-β-*D*-glucopyranoside (3):

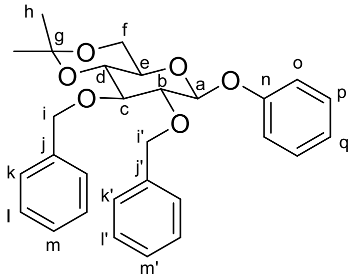

4,6-*O*-isopropylidene-phenyl-β-*D*-glucopyranoside (4.2 g, 14 mmol) was dissolved in dry DMF (50 mL) under a N_2 atmosphere. The solution was cooled to 0 °C and stirred vigorously. NaH (50% weight in mineral oil, 1.68 g, 35 mmol) was carefully added to the solution and stirred at 0 °C for 20 min. Benzyl bromide (3.5 mL, 29 mmol) was added dropwise to the reaction mixture. The suspension was stirred at 0 °C for additional 5 min and then allowed to warm to room temperature and stirred overnight. The reaction was quenched with a few drops of water. The suspension was extracted with ethyl acetate (120 mL) and water (50 mL). The aqueous layer was re-extracted with ethyl acetate (3 x 25 mL) and the combined organic layers were washed with 5% aqueous LiCl solution (2 x 25 mL) and brine (25 mL). The organic solution was then dried over $MgSO_4$, filtered and concentrated under reduced pressure. The crude mixture was separated by MPLC (hexane : ethyl acetate = 100 : 0 (4 min) -60 : 40 (over 15 min) - 0 : 100 (over 1 min)) to yield **3** as a colourless oil (6 g, 12.5 mmol, 90% yield).

^{1}H NMR (400 MHz, $CDCl_3$) δ 7.40 – 7.25 (m, 12H, $H_{k+k'}$ + $H_{l+l'}$ + $H_{m+m'}$ + H_p), 7.12 – 6.99 (m, 3H, H_o + H_q), 5.10 (d, *J* = 7.3 Hz, 1H, H_a), 4.96 (d, *J* = 10.9 Hz, 1H, H_i), 4.92 – 4.77 (m, 3H, $H_{i+i'}$), 3.98 (dd, *J* = 10.9, 5.4 51 Hz, 1H, H_f), 3.88 – 3.75 (m, 2H, H_d + $H_{f'}$), 3.75 – 3.64 (m, 2H, H_b + H_c), 3.39 (td, *J* = 10.0, 5.4 Hz, 1H, H_e), 1.53 (s, 3H, H_h), 1.46 (s, 3H, $H_{h'}$).

^{13}C NMR (101 MHz, $CDCl_3$) δ 157.2 (C_n), 138.8, 138.3 (2 x C, $C_{j+j'}$), 129.7, 128.5, 128.4, 128.3, 128.1, 127.9, 127.7 (7 x C, $C_{k+k'}$ + $C_{l+l'}$ + $C_{m+m'}$ + C_p), 123.1 (C_q), 117.0 (C_o), 102.1 (C_a), 99.5 (C_g), 81.9, 81.5 (2 x C, C_b + C_c), 75.7, 75.1 (2 x C, $C_{i+i'}$), 74.2 (C_d), 67.2 (C_e), 62.4 (C_f), 29.3, 19.3 (2 x C, $C_{h+h'}$).

HR-ESI-MS: m/z: 499.2091 $[M+Na]^+$, calculated for $C_{29}H_{32}NaO_6^+$: 499.2091

2,3-di-*O*-benzyl-phenyl-β-*D*-glucopyranoside:

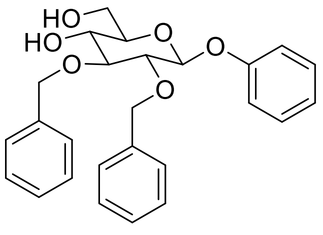

Compound **3** (1.8 g, 3.8 mmol) was suspended in a solution of acetic acid/water (20 mL, v:v = 3:2). The suspension was stirred at 50 °C for 90 mins. After cooling to room temperature, toluene (150 mL) was added to the mixture and the solvents were removed under reduced pressure to give the product as a white solid which was used in the next step without purification (1.58 g, 3.6 mmol, 95 % yield).

4-*O*-allyl-2,3-di-*O*-benzyl-phenyl-β-*D*-glucopyranoside (4):

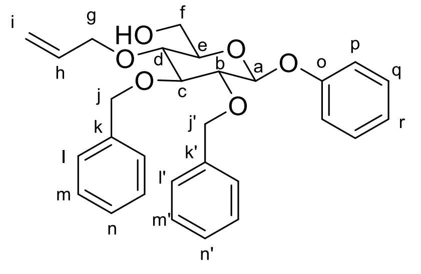

Crude compound 2,3-di-*O*-benzyl-phenyl-β-*D*-glucopyranoside (1.7 g, 3.9 mmol) was dissolved in dry DMF (10 mL) under N_2 atmosphere. NaH (60% weight in mineral oil, 343 mg, 8.6 mmol) was added and the reaction was stirred for 15 minutes at room temperature. Allyl bromide (337 µL, 3.9 mmol) was added dropwise to the solution and then the mixture was stirred overnight. The reaction was quenched with careful addition of water (30 mL). Ethyl acetate (30 mL) was added and the layers separated. The aqueous layer was extracted with ethyl acetate (3 x 30 mL). The combined organic layers were washed with 5% aqueous LiCl solution (2 x 20 mL), brine (20 mL) and dried with $MgSO_4$. The solution was then filtered and concentrated under reduced pressure. The crude product was then purified by MPLC (hexane : ethyl acetate = 100 : 0 (4 min) - 50 : 50 (over 15 min) - 0 : 100 (over 1 min)) to yield **4** in the form of a white solid (750 mg, 1.56 mmol, 40% yield).

^{1}H NMR (400 MHz, $CDCl_3$) δ 7.39 – 7.23 (m, 12H, $H_{l+l'}$ + $H_{m+m'}$ + $H_{n+n'}$ + H_q), 7.11 – 6.99 (m, 3H, H_p + H_r), 5.98 – 5.81 (m, 1H, H_h), 5.26 (dq, *J* = 17.2, 1.6 Hz, 1H, H_i), 5.17 (ddd, *J* = 10.4, 3.1, 1.9 Hz, 1H, $H_{i'}$), 5.12 – 4.88 (m, 3H, H_a + H_j), 4.86 – 4.73 (m, 2H, $H_{j'}$), 4.38 – 4.12 (m, 1H, H_g), 4.04 (dt, *J* = 5.7, 1.6 Hz, 1H, H_e), 3.93 (dd, *J* = 12.1, 2.2 Hz, 1H, $H_{g'}$), 3.82 – 3.62 (m, 3H, H_c + H_f), 3.62 – 3.44 (m, 2H, H_b + H_d).

^{13}C NMR (101 MHz, $CDCl_3$) δ 157.2 (C_o), 138.6, 138.3 ($C_{k+k'}$), 134.7, 134.5 ($C_{h+h'}$), 129.7, 128.7, 128.6, 128.4, 128.2, 128.0, 127.9, (7 x C, $C_{l+l'}$ + $C_{m+m'}$ + $C_{n+n'}$ + C_q), 123.0, (C_r), 117.6 (C_i) 117.0 (C_p), 101.9 (C_a), 84.2, 81.6, (C_b + C_c), 75.9, 75.5, 75.3, ($C_{j+j'}$ + C_g), 74.4 (C_d), 72.8 (C_e), 70.2 (C_h), 62.2 (C_f).

HR-ESI-MS: m/z: 494.2533 $[M+NH_4]^+$ calculated for $C_{29}H_{36}NO_6^+$: 494.2537.

4-*O*-allyl-2,3-di-*O*-benzyl-4-*O*-(2-(2-chloroethoxy)ethoxy)-phenyl-β-*D*-glucopyranoside:

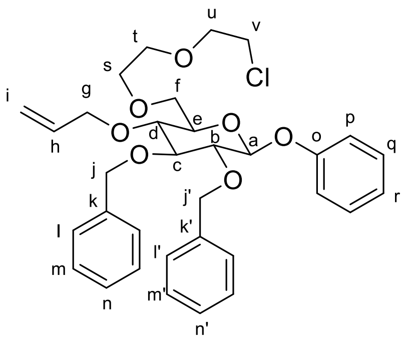

2,2'-Dichlorodiethyl ether (3.9 mL, 33.6 mmol) and Bu_4NHSO_4 (45.6 mg, 134 µmol) were dissolved in a 50% aqueous KOH solution (3 mL). A solution of **4** (750 mg, 1.56 mmol) in THF (1.0 mL) was added dropwise to the reaction mixture under vigorous stirring. The suspension was stirred at 50 °C overnight. After cooling to room temperature, DCM (20 mL) and water (25 mL) were added and the layers separated. The aqueous layer was re-extracted with DCM (3 x 25 mL) and the combined organic layers were dried with $MgSO_4$. The solution was filtered and concentrated under reduced pressure. The crude mixture was separated by MPLC (hexane : ethyl acetate = 100 : 0 (5 min) - 70 : 30 (over 14 min) - 0 : 100 (over 1 min)) to yield the product as a pale yellow oil (895 mg, 1.4 mmol, 90% yield).

^{1}H NMR (400 MHz, $CDCl_3$) δ 7.36 – 7.24 (m, 12H, $H_{l+l'}$ + $H_{m+m'}$ + $H_{n+n'}$ + H_q), 7.09 – 7.01 (m, 3H, H_p + H_r), 5.90 (ddt, *J* = 17.1, 10.4, 5.6 Hz, 1H, H_h), 5.26 (ddd, *J* = 17.2, 3.3, 1.7 Hz, 1H, H_i), 5.18 – 5.12 (m, 1H, $H_{i'}$), 5.05 – 4.88 (m, 3H, H_j + H_a), 4.86 – 4.78 (m, 2H, $H_{j'}$), 4.37 – 4.13 (m, 1H, H_g), 4.11 – 3.95 (m, 2H, H_e + $H_{g'}$), 3.85 – 3.47 (m, 13H, H_b + H_c + H_d + H_f + H_s + H_t + H_u + H_v).

^{13}C NMR (101 MHz, $CDCl_3$) δ 157.6 (C_s), 138.8, 138.4 ($C_{o+o'}$), 134.9 ($C_{l+l'}$), 129.6, 128.5, 128.4, 128.1, 127.9, 127.8, 127.5, 122.8 (7C, $C_{p+p'}$, $C_{q+q'}$, C_t, C_r, C_u, C_v), 117.2 (C_m), 117.0 (C_i), 101.8 (C_a), 84.6, 82.0, (C_b + C_c) 78.4 ($C_{e,}$) 75.9, 75.8, 75.4 ($C_{n+n'}$ + C_g), 75.2, 74.0, 72.6, 71.5 (C_d + C_s + C_t + C_u), 70.2 (C_h), 68.8 (C_f), 42.9 (Cv).

HR-ESI-MS: m/z: 600.2718 $[M+NH_4]^+$ calculated for $C_{33}H_{43}ClNO_7^+$: 600.2723.

4-*O*-allyl-2,3-di-*O*-benzyl-4-*O*-(tetraethylene glycolyl)-phenyl-β-*D*-glucopyranoside (5):

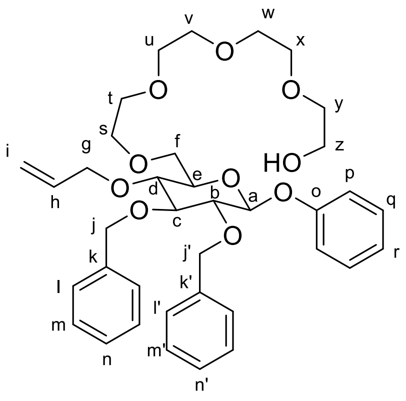

To a solution of KOH (3.15 g, 56.2 mmol) in diethylene glycol (20 mL, 150 mmol) at 50 °C, 4-*O*-allyl-2,3-di-*O*-benzyl-4-*O*-(2-(2-chloroethoxy)ethoxy)-phenyl-β-*D*-glucopyranoside (750 mg, 1.2 mmol) dissolved in THF (1 mL) was added. The solution was stirred at 80 °C overnight. After cooling to room temperature, water (20 mL) and DCM (20 mL) were added. The layers were separated and the aqueous layer was acidified with 1 N HCl until the pH of the solution was below 3. The aqueous layer was subsequently re-extracted with DCM (3 x 20 mL) and the combined organic layers were washed with brine and dried with $MgSO_4$. The solution was filtered and concentrated under reduced pressure. The product was then purified by MPLC (hexane : ethyl acetate = 100 : 0 (2 min) - 60 : 40 (over 3 min) - 0 : 100 (over 20 min) to yield **5** in the form of a pale yellow oil (670 mg, 1 mmol µmol, 85% yield).

^{1}H NMR (400 MHz, $CDCl_3$) δ 7.38 – 7.25 (m, 12H, $H_{l+l'}$ + $H_{m+m'}$ + $H_{n+n'}$ + H_q), 7.08 – 7.01 (m, 3H, H_p + H_r), 5.90 (ddt, *J* = 17.2, 10.4, 5.6 Hz, 1H, H_h), 5.29 – 5.23 (m, 1H, H_i), 5.15 (ddd, *J* = 10.4, 4.4, 1.3 Hz, 1H, $H_{i'}$), 5.05 – 4.77 (m, 5H, H_a +$H_{j+j'}$), 4.37 – 4.13 (m, 1H, H_g), 4.10 – 3.95 (m, 2H, H_e + $H_{g'}$), 3.85 – 3.75 (m, 2H), 3.72 – 3.46 (m, 20H) (H_b + H_c + H_d + H_f + H_s + H_t + H_u + H_v + H_w + H_x + H_y + H_z).

HR-ESI-MS: m/z: 670.3582 $[M+NH_4]^+$, calculated for $C_{37}H_{52}NO_{10}^+$: 670.3586

4-*O*-allyl-2,3-di-*O*-benzyl-glucose crown ether:

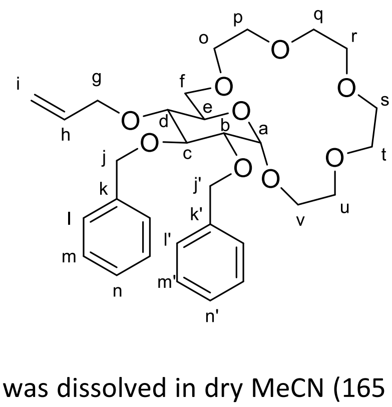

Compound **5** (560 mg, 850 µmol) was dissolved in dry MeCN (165 mL) in a N_2 atmosphere. KBF_4 (212 mg, 1.69 mmol) was added and the solution was stirred at room temperature for 1 hour. $FeCl_3$ (301 mg, 1.85 mmol) was then added and the solution was stirred at 40 ^{o}C for 48 hours. The reaction was then cooled to room temperature and concentrated to 10% volume under reduced pressure. DCM (30 mL) and 1 M aqueous $NaHCO_3$ (30 mL) were added, and the layers separated. The aqueous layer was extracted with DCM (3 x 30 mL). The combined organic layers were washed with water (30 mL), brine (30 mL) and dried with $MgSO_4$. The solution was filtered, concentrated under reduced pressure, and

the crude mixture was purified by MPLC (hexane : ethyl acetate = 100 : 0 (3 min) - 40 : 60 (over 16 min) - 0 : 100 (over 1 min)) to yield the product as a pale yellow oil (305 mg, 550 µmol, 65% yield).

^{1}H NMR (400 MHz, $CDCl_3$) δ 7.44 – 7.25 (m, 10H, $H_{l+l'}$ + $H_{m+m'}$ + $H_{n+n'}$), 5.95 (ddt, *J* = 17.3, 10.4, 5.6 Hz, 1H, H_h), 5.28 (ddd, *J* = 17.2, 1.7, 1.4 Hz, 1H, H_i), 5.16 (ddd, *J* = 10.4, 1.7, 1.4 Hz, 1H, $H_{i'}$), 4.97 – 4.80 (m, 3H, $H_{j+j'}$), 4.72 (d, *J* = 3.4 Hz, 1H, H_a), 4.68 (d, *J* = 12.4 Hz, 1H, $H_{j'}$), 4.36 (ddd, *J* = 10.0, 3.1, 1.9 Hz, 1H, H_e), 4.12 – 3.97 (m, 4H, H_c + H_e + H_g), 3.91 – 3.44 (m, 20H, H_b + H_d+ H_f+ H_o+ H_p+ H_q+ H_r+ H_s+ H_t+ H_u+ H_v).

^{13}C NMR (101 MHz, $CDCl_3$) δ 139. 6, 138.7 ($C_{k+k'}$), 135.1 (C_h), 128.5, 128.4, 128.2, 128.0, 127.9, 127.5 ($C_{l+l'}$ + $C_{m+m'}$ + $C_{n+n'}$), 116.8 (C_i), 96.8 (C_a), 81.1 (C_c), 79.5 (C_b), 77.3 (C_d), 75.5, 73.4 (2 x C, $C_{j+j'}$), 72.3 (C_g), 72.2, 71.8, 71.0, 70.9, 70.8, 70.6, 69.7 (C_o + C_p + C_q + C_r + C_s + C_t + C_u), 69.3 (C_e), 68.9 (C_v), 66.7 (C_f).

HR-ESI-MS: m/z: 581.2709 $[M+Na]^+$, calculated for $C_{31}H_{42}NaO_9^+$: 581.2721

2,3-di-*O*-benzyl-glucose crown ether (6):

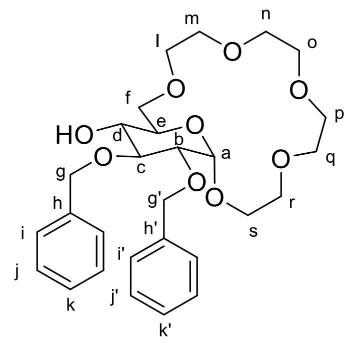

4-*O*-allyl-2,3-di-*O*-benzyl-glucose crown ether (280 mg, 500 µmol) and *p*-toluenesulphonic acid monohydrate (70 mg, 360 µmol) were dissolved in a solution of MeOH/water (36 mL, v:v = 5:1). Pd/C (120 mg) was then added and the suspension was heated to reflux for 2 hours. The reaction was cooled to room temperature and Et3N (1 mL) was added. The suspension was filtered through celite, concentrated under reduced pressure and separated by MPLC (DCM:MeOH = 100 : 0 (3 min) - 90 : 10 (over 11 min) - 0 : 100 (over 1 min)) to yield the product as a colourless oil (230 mg, 450 µmol, 90% yield).

^{1}H NMR (400 MHz, $CDCl_3$) δ 7.43 – 7.24 (m, 10H, $H_{i+i'}$ + $H_{j+j'}$ + $H_{k+k'}$), 4.92 (d, *J* = 11.2 Hz, 1H, H_a), 4.86 – 4.78 (m, 2H, Hg), 4.69 – 4.61 (m, 2H, Hg'), 4.37 (ddd, *J* = 10.2, 5.5, 2.5 Hz, 1H, H_e), 4.09 – 4.00 (m, 2H, H_c + H_f), 3.88 – 3.35 (m, 19H, H_b + H_d + $H_{f'}$ + H_l + H_m + H_n + H_o + H_p + H_q + H_r + H_s).

^{13}C NMR (101 MHz, $CDCl_3$) δ 139.4, 138.6 ($C_{h+h'}$), 128.6, 128.4, 128.2, 128.0, 127.6 ($C_{i+i'}$ + $C_{j+j'}$ + $C_{k+k'}$), 96.9 (C_a), 80.1 (C_c), 79.9 (C_b), 78.5 (C_d), 75.4, 73.5 ($C_{g+g'}$), 71.5, 70.9, 70.8, 70. 7, 70.6, 70.5 (C_m + C_n + C_o + C_p + C_q + C_r), 70.4 (C_e), 69.0 (C_l), 67.2 (C_s), 63.1 (C_f).

HR-ESI-MS: m/z: 541.2402 $[M+Na]^+$, calculated for $C_{28}H_{38}NaO_9^+$: 541.2408

(E)-4,4'-(diazene-1,2-diyl)bis(3,5-difluorobenzoic acid) :

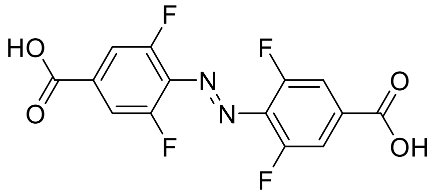

4,4'-(Diazene-1,2-diyl)bis(3,5-difluorobenzoic acid) was prepared following a previously reported literature procedure.[1]

(E)-4,4'-(diazene-1,2-diyl)bis(3,5-difluorobenzoyl chloride) (7):

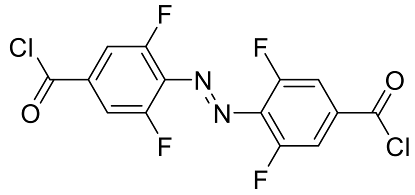

4,4'-(Diazene-1,2-diyl)bis(3,5-difluorobenzoic acid) (15 mg, 45 µmol, 1.00 equiv.) was suspended in thionyl chloride (6 mL) and then refluxed for 5 h (upon heating, dissolution of the acid was observed). After cooling of the solution to room temperature, the excess of thionyl chloride was removed under reduced pressure. The red residue was then redissolved in dry toluene (5 mL) and the solvent was again removed under reduced pressure to yield the crude product 7 (16 mg, 43 µmol, 95 %) as a red solid which was used in the following reaction without purification.

Bn-protected receptor 1:

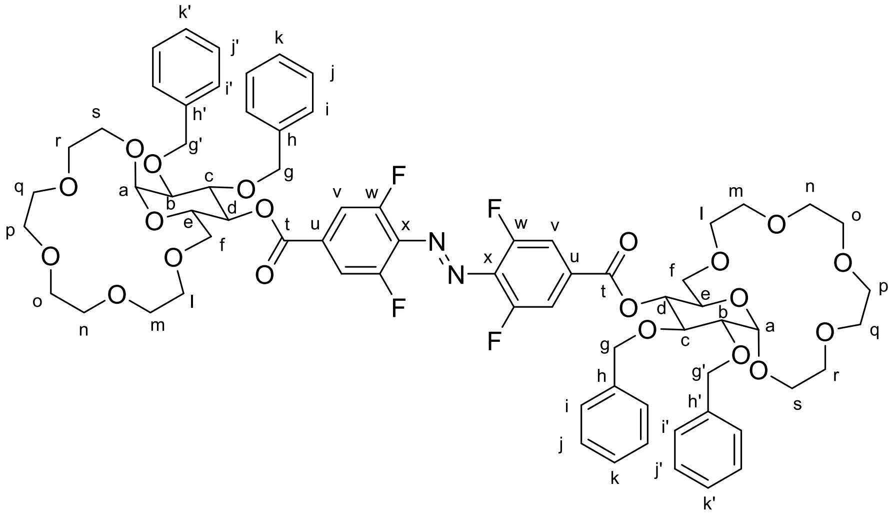

Compound **7** (16 mg, 43 µmol, 1 equiv.) was dissolved in dry THF (1 mL) under N_2 atmosphere. Compound **6** (60 mg, 117 µmol, 2.7 equiv.) was also dissolved in dry THF (1.5 mL) and added to the solution. Subsequently, triethylamine (0.2 mL) was added, and the reaction mixture was stirred overnight at room temperature. The volatiles were then removed under reduced pressure and the crude product was purified by preparative thin-layer chromatography (eluent: 2% MeOH in CH_2Cl_2) to yield the product as a red solid (29 mg, 21.5 µmol, 50%).

^{1}H NMR (400 MHz, $CDCl_3$) δ 7.79 (d, *J* = 8.8 Hz, 3.6 H, H_v *E* isomer), 7.58 (d, *J* = 7.3 Hz, 0.4 H, H_v *Z* isomer), 7.48 – 7.21 (m, 20H, $H_{i+i'}$ + $H_{j+j'}$ + $H_{k+k'}$), 5.00 – 4.92 (m, 4H, $H_g/H_{g'}$), 4.92 – 4.83 (m, 4H, $H_g/H_{g'}$), 4.77 (d, *J* = 12.1 Hz, 2H, H_a), 4.74 – 4.66 (m, 4H, H_d + H_e), 4.10 (dd, *J* = 9.6, 8.7 Hz, 2H, H_c), 3.95 – 3.46 (m, 38H, H_b + H_f + H_l + H_m + H_n + H_o + H_p + H_q + H_r + H_s).

^{13}C NMR (101 MHz, $CDCl_3$) δ 163.6 (C_t), 156.3, 153.7 (C_w), 139.3, 139.0, 138.5, 138.4 ($C_{h+h'}$), 134.2 (C_x), 133.7 (C_u), 128.6, 128. 5, 128.4, 128.4, 128.3, 128.1, 128.1, 128.0, 127.9, 127.8, 127.62, 127.4 ($C_{i+i'}$ + $C_{j+j'}$ + $C_{k+k'}$), 114.2, 114.0 (C_v), 96.7, 96.4 (C_a), 80.6 (C_c), 79.9, 79.60 (C_b), 78.4, 78.2 (C_d), 75.8, 75.2, 73.4, 73.3 ($C_{g+g'}$), 72.0, 71.4, 70.9, 70.8, 70.7, 70.7, 70.6, 70.5, 70.5, 70.4, 70.3, 70.0, 68.8 (C_l + C_m + C_n + C_o + C_p + C_q + C_r), 67.8 (C_e), 67.1, 66.8 (C_s), 62.9 (C_f). *– several peaks are 'doubled' as they originate from the E- and Z- isomer of the compound*

HR-ESI-MS: m/z: 1365.4971 $[M+Na]^+$, calculated for $C_{70}H_{78}F_4N_2NaO_{20}^+$: 1365.4976

Bn-protected receptor 2:

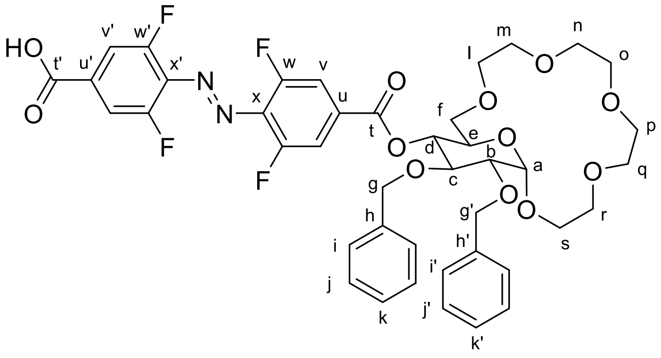

The product was obtained following the same synthetic protocol as used for the preparation of Bn-protected **1** but with a smaller excess of building block **6** in the reaction (1.5 equiv. instead of 2.7 equiv). The product was obtained as a red solid in 60% yield.

^{1}H NMR (400 MHz, CD_3OD) δ 7.85 (d, *J* = 9.1 Hz, 1.4H, $H_{v'}$ – E isomer), 7.78 (d, *J* = 10.4 Hz, 1.4H, H_v – E isomer), 7.67 (d, *J* = 8.5 Hz, 0.6H, $H_{v'}$ – Z isomer), 7.59 (d, *J* = 9.3 Hz, 0.6H, H_v – Z isomer), 7.49 – 7.23 (m, 10H, $H_{i+i'}$ + $H_{j+j'}$ + $H_{k+k'}$), 4.80 – 4.66 (m, 7H, H_a + H_d + H_e + $H_{g+g'}$), 4.06 – 3.99 (m, 1H, H_c), 3.94 – 3.85 (m, 2H, H_f), 3.84 – 3.41 (m, 17H, H_b + H_l + H_m + H_n + H_o + H_p + H_q + H_r + H_s).

^{13}C NMR (101 MHz, CD_3OD) δ 172.4 ($C_{t'}$), 165.1 (C_t), 153.4, 151.0 (C_w + $C_{w'}$), 139.6, 138.3 ($C_{h+h'}$), 133.7 ($C_{x/x'}$), 133.2 ($C_{u/u'}$), 128.0, 127.9, 127.8, 127.7, 127.6, 127.5, 127.2 ($C_{i+i'}$ + $C_{j+j'}$ + $C_{k+k'}$), 113.2 ($C_{v/v'}$), 96.0 (C_a), 81.3 (C_c), 80.2 (C_b), 77.7 (C_d), 75.0, 72.7, 71.7, 70.5, 70.2 69.3, 67.7, 66.7 (C_b + C_m + C_n + C_o + C_p + C_q + C_r + C_s), 65.1 (C_f). *– several peaks are 'doubled' as they originate from the E- and Z- isomer of the compound*

HR-ESI-MS: m/z: 865.2559 $[M+Na]^+$, calculated for $C_{42}H_{42}F_4N_2NaO_{12}^+$: 865.2566

Receptor 1:

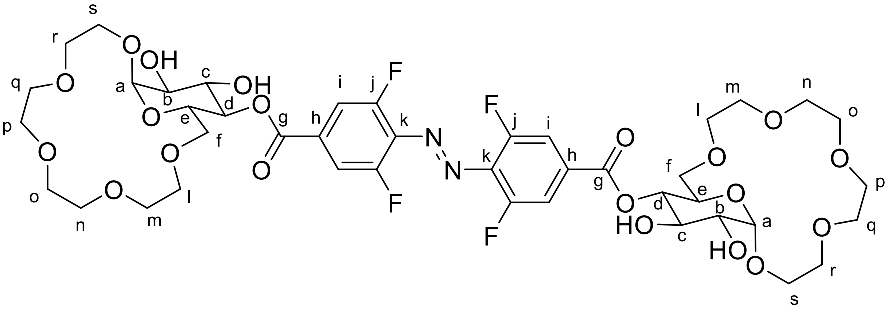

The Bn-protected **1** (25 mg, 18.5 µmol, 1.00 equiv.) was dissolved in EtOAc (0.5 mL) and an aqueous $NaBrO_3$ solution (0.3 mL, c = 185 mg/mL) was added. Subsequently, an aqueous NaS_2O_4 solution (0.4 mL, c = 154 mg/mL) was added dropwise to the reaction mixture and the biphasic system was stirred vigorously for 72 h at room temperature. Subsequently it was diluted with EtOAc (5 mL), washed with water (4 mL) and 1% aqueous sodium thiosulfate solution (2 mL). The layers were separated, and the aqueous layer was extracted with EtOAc (3 x 5 mL). All organic layers were combined, washed with water (5 mL), dried over Na_2SO_4, filtered and concentrated under reduced pressure. The crude product was purified by preparative thin-layer chromatography (eluent: 5% MeOH in CH_2Cl_2) to yield receptor **1** as a red solid (11 mg, 11 µmol, 60%).

^{1}H NMR (400 MHz, CD_3OD) δ 7.88 (d, *J* = 9.0 Hz, 3H, H_i – E isomer), 7.69 (d, *J* = 7.0 Hz, 1H, H_i – Z isomer), 4.79 (d, *J* = 3.5 Hz, 2H, H_a), 4.77 – 4.71 (m, 2H, H_d), 4.68 (ddd, *J* = 10.2, 4.5, 2.1 Hz, 2H, H_e), 4.01 – 3.89 (m, 6H, H_c + H_f), 3.80 – 3.52 (m, 32H, H_l + H_m + H_n + H_o + H_p + H_q + H_r + H_s), 3.51 – 3.43 (m, 2H, H_b).

^{13}C NMR (101 MHz, CD_3OD) δ 163.4 (C_g), 156.2, 153.6 (C_j), 137.7 (C_k), 134.1 (C_h), 113.7, 113.5 (C_i), 98.3 (C_a), 77.5 (C_b), 72.4 (C_d), 72.4 (C_d), 71.6 (C_c), 71.5, 71.3, 70.5, 70.3, 70.2, 70.1, 68.1, 68.0 (C_l + C_m + C_n + C_o + C_p + C_q + C_r), 67.5 (C_e), 66.82 (C_s), 65.3 (C_f). – *several peaks are 'doubled' as they originate from the E- and Z- isomer of the compound*

^{19}F NMR (377 MHz, CD_3OD) δ -120.4 (Z isomer), -121.6 (E isomer).

HR-ESI-MS: m/z: 514.1497 $[M+2Na]^{2+}$ calculated for $C_{42}H_{54}F_4N_2Na_2O_{20}^{2+}$ = 514.1495.

Receptor 2:

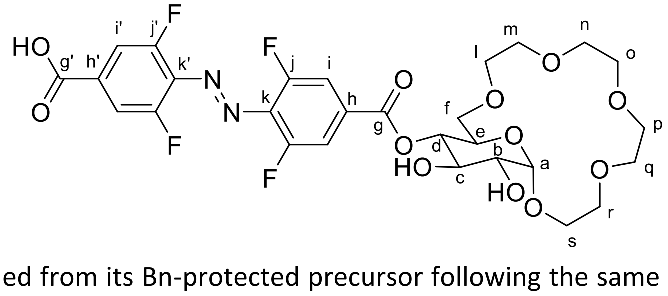

Receptor **2** was obtained from its Bn-protected precursor following the same synthetic procedure as used to prepare compound **1**. **2** was obtained as a red solid in 65% yield.

^{1}H NMR (400 MHz, CD_3OD) δ 7.86 (d, *J* = 8.9 Hz, 1.4H, H_i – E isomer), 7.74 (d, *J* = 10.6 Hz, 1.4H, $H_{i'}$ – E isomer), 7.67 (d, *J* = 8.4 Hz, 0.6H, H_i – Z isomer), 7.53 (d, *J* = 9.7 Hz, 0.6H, $H_{i'}$ – Z isomer), 4.80 (d, *J* = 3.8 Hz, 1H, H_a), 4.77 – 4.71 (m, 1H, H_d), 4.71 – 4.65 (m, 1H, H_e), 4.02 – 3.89 (m, 3H, H_c + H_f), 3.84 – 3.50 (m, 16H, H_l + H_m + H_n + H_o + H_p + H_q + H_r + H_s), 3.50 – 3.43 (m, 1H, H_b).

^{13}**C NMR** (101 MHz, CD_3OD) δ 173.1 ($C_{g'}$), 165.2 (C_g), 155.8, 153.7 ($C_{j+j'}$), 133.9 ($C_{k+k'}$), 131.9($C_{h+h'}$), 113.6, 113.3 ($C_{i+i'}$), 98.2 (C_a), 77.5 (C_b), 72.4 (C_d), 71.6 (C_c), 71.3, 70.5, 70.5, 70.3, 70.20, 70.1, 68.1 (C_l + C_m + C_n + C_o + C_p + C_q + C_r), 67.5 (C_e), 66.8 (C_s), 65.3 (C_f).

^{19}**F NMR** (377 MHz, CD_3OD) δ -120.5, -122.0 (Z isomer), -122.2, -122.6 (E isomer).

HR-ESI-MS: m/z: 685.1623 $[M+Na]^+$ calculated for $C_{28}H_{30}F_4N_2NaO_{12}^+$ = 685.1627.

Supplementary Note 2.

Photostationary states determination.

2.1. UV-Vis measurements.

The measurements were performed in 1 mL quartz cuvettes (pathway length = 1 cm) in Milli-Q pure H_2O. The concentration of the solutions was 100 µM. The samples were irradiated with light at wavelengths of 410 nm and 623 nm for a given period of time and the spectra were subsequently measured. The irradiations were continued until no further changes in the UV-Vis spectra of the receptors was observed, indicating that the photostationary state (PSS) was reached (Table S1).

λ	Receptor **1**			Receptor **2**		
	Irradiation time	% E	% Z	Irradiation time	% E	% Z
410 nm	15 s	>95	<5	15 s	>95	<5
623 nm	160 min	<5	>95	160 min	10	90

Table S1. *Times to reach PSS and the E/Z ratio at PSS for both receptors.*

UV-Vis spectra - receptor 1:

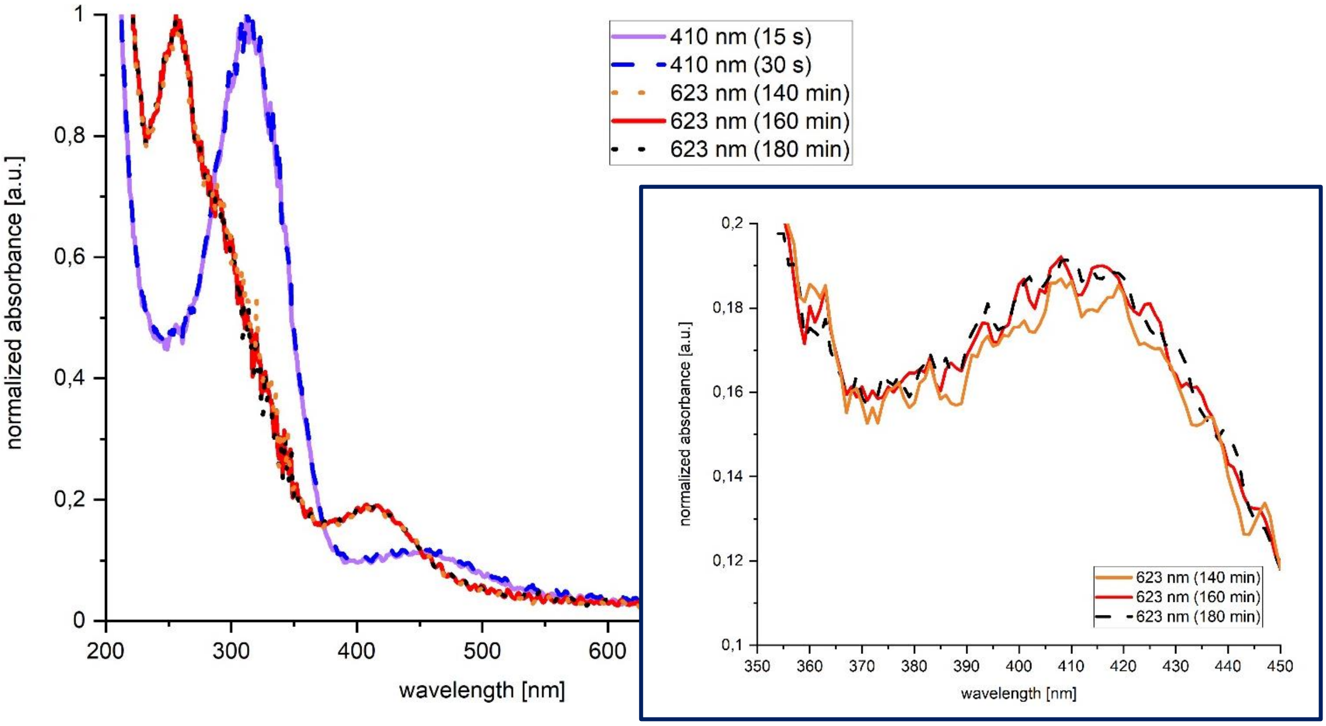

Fig. S1 *UV-Vis spectra of receptor **1** in Mili-Q H_2O recorded after irradiation of the solution with light of 410 nm and 623 nm wavelength for the indicated time (inset – enlarged 350 – 450 nm region of the spectrum to indicate minor changes in the intensity of the absorption maxima upon 160 min irradiation compared to 140 min).*

UV-Vis spectra - receptor 2:

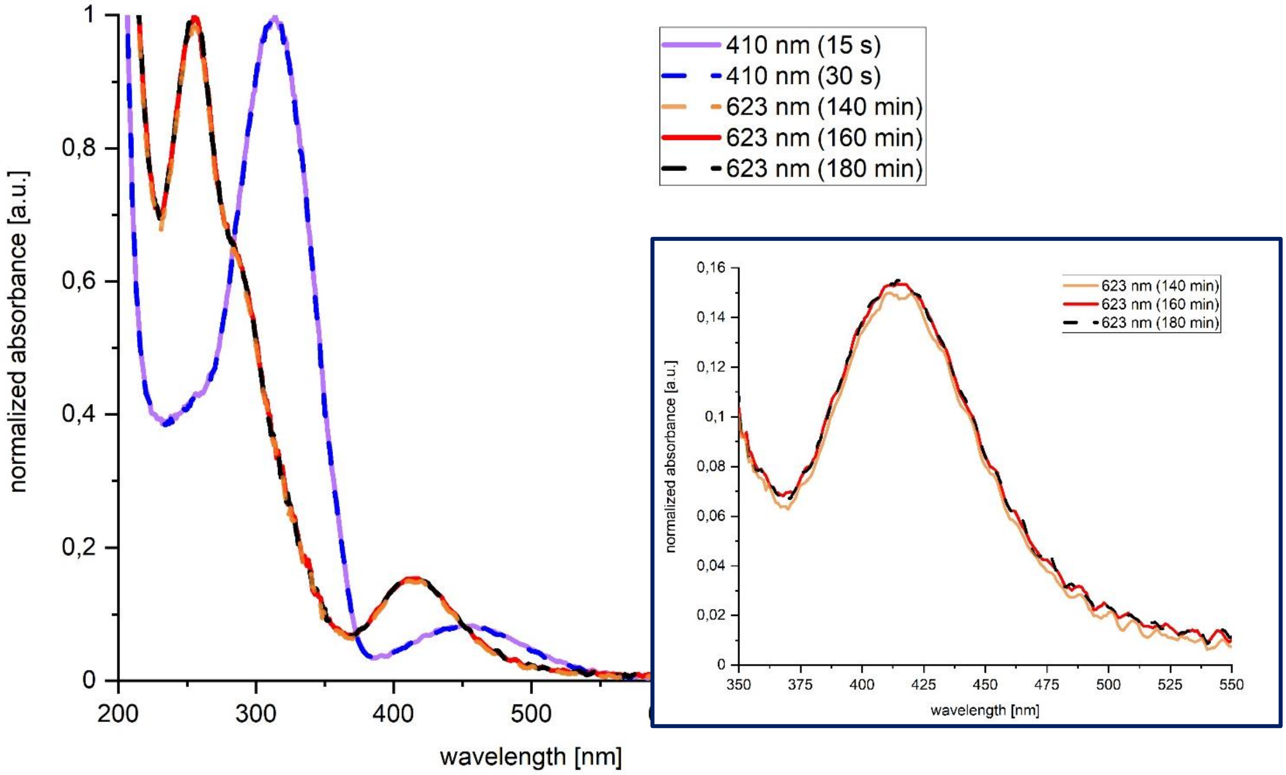

Fig. S2 *UV-Vis spectra of receptor* ***2*** *in Milli-Q* H_2O *recorded after irradiation of the solution with light of 410 nm and 623 nm wavelength for the indicated time (inset – enlarged 350 – 450 nm region of the spectrum to indicate minor changes in the intensity of the absorption maxima upon 160 min irradiation compared to 140 min).*

2.2. NMR measurements.

The measurements were performed in D_2O at a sample concentration of 1 mM. The samples were irradiated for the times required to reach the PSS, established in the UV-Vis measurements. ^{1}H NMR spectra were subsequently recorded to determine the E/Z isomer ratio of each receptor at PSS.

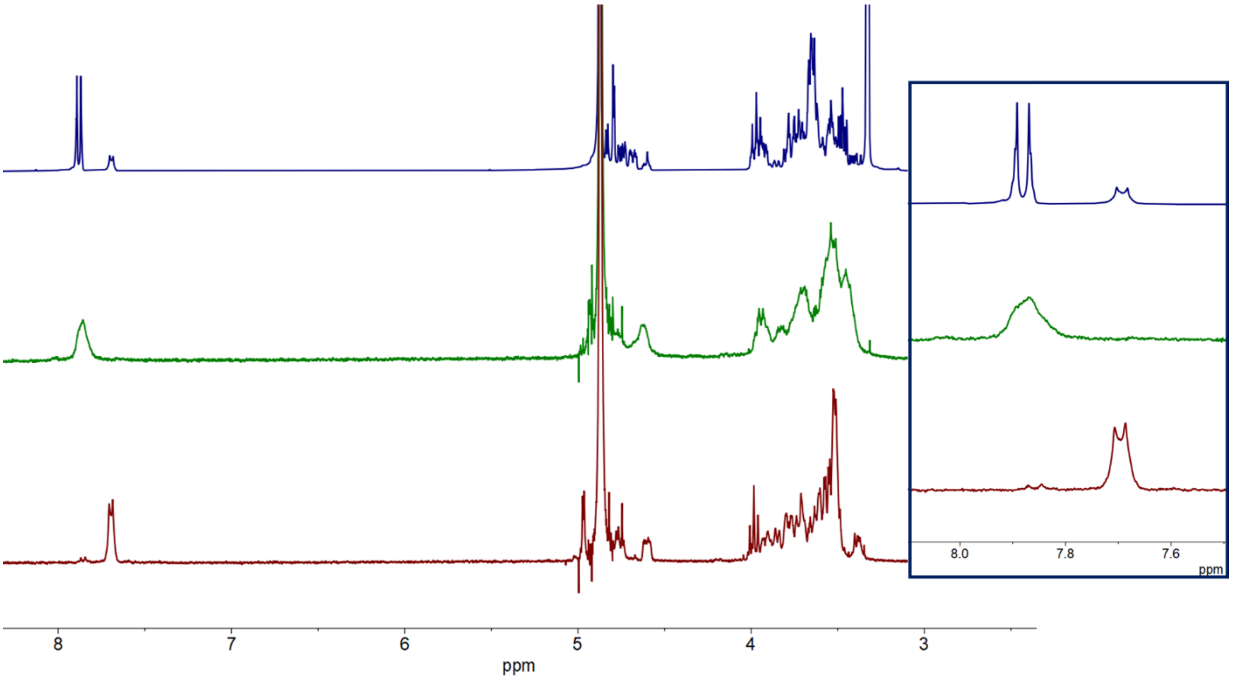

Fig. S3 *Stacked plot of ^{1}H NMR spectra of a D_2O solution of receptor* ***1****: top – after synthesis; middle – after irradiation with light at 410 nm wavelength for 15 s; bottom – after irradiation with light at 623 nm wavelength for 160 min. Inset – enhanced region of the spectra where signals of aromatic protons are found.*

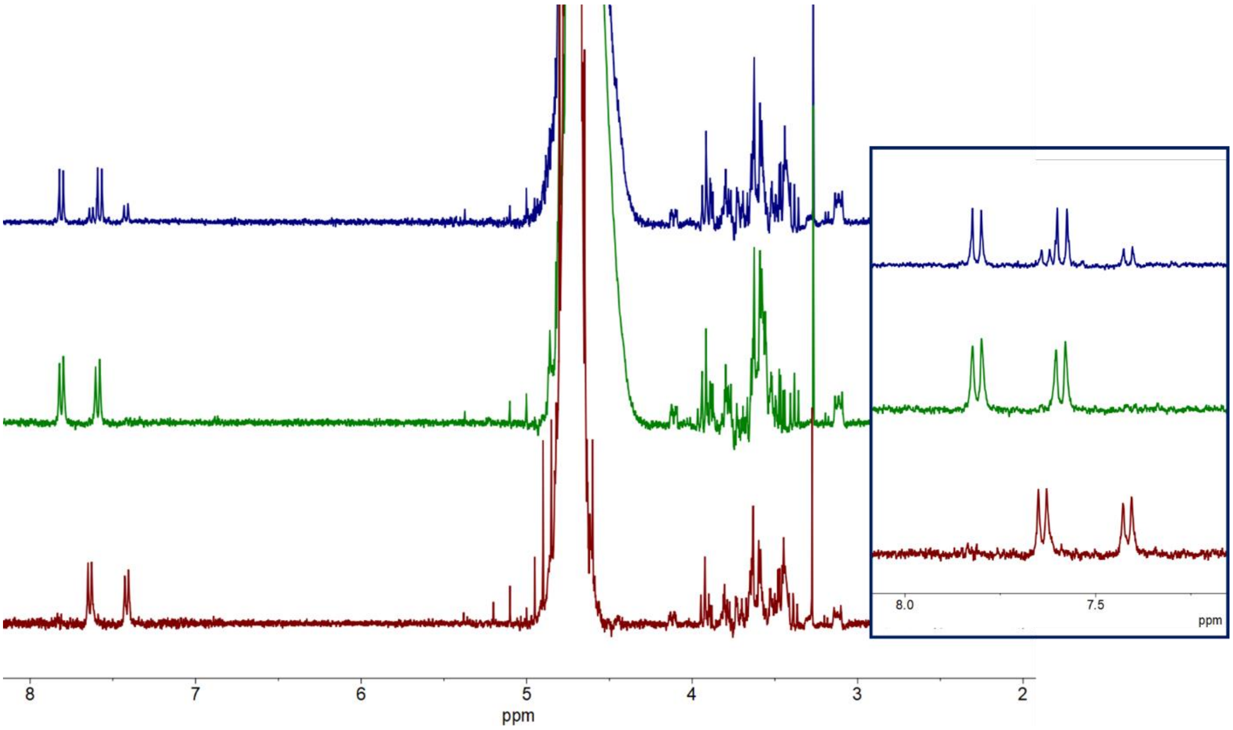

Fig. S4 *Stacked plot of ^{1}H NMR spectra of a D_2O solution of receptor* ***2****: top – after synthesis; middle – after irradiation with light at 410 nm wavelength for 15 s; bottom – after irradiation with light at 623 nm wavelength for 160 min. Inset – enhanced region of the spectra where signals of aromatic protons are found.*

Supplementary Note 3.

Photoisomerization quantum yield measurements.

The maximum isomerization quantum yield was determined, using the experimental setup developed by Riedle and coworkers:[2] for the E → Z direction, by illumination with a LED with 528 nm nominal emission wavelength (Osram OSLON SSL80 LTCP7P-KXKZ); for the reverse Z → E direction with a LED with 400 nm nominal emission maximum (Luxeon LHUV-0400-0450).

First, receptor ***E*-1** was dissolved in H_2O and diluted to a final concentration of 50 µM and illuminated with 528 nm for defined time intervals, after which a UV-Vis absorption spectrum was recorded (Fig.S5 – left). The illumination was continued until the PSS was reached.

To determine the *E*/*Z*-ratio after each illumination interval, the molar absorptivity of the *Z*-isomer had to be determined first. To achieve this, the sample was lastly illuminated with 620 nm, in order to correlate the previously determined *E*-/*Z*-ratio from the NMR-studies (see SI, chapter 3) with the obtained absorption spectrum. Thus ε_Z can be determined (equation 1), with which the *E*-/*Z*-ratio can be calculated for each illumination step during measurement, using equation 2:

$$\varepsilon_Z(\lambda) = \frac{1}{a} \cdot (\varepsilon_{PSS}(\lambda) - b \cdot \varepsilon_E(\lambda)) \quad (1)$$

$$c_E = \frac{A_{tot} - \varepsilon_Z c_{tot}}{\varepsilon_E - \varepsilon_Z} \quad (2)$$

Where ε denotes the extinction coefficient of the respective isomer or at the PSS, c is the concentration of the respective species, A_{tot} is the total absorption of the isomeric mixture, *a* is defined as the percentage of the product at the PSS and *b* as the percentage of the substrate at the same specified PSS.

To determine the reaction quantum yield in the Z→E direction, a sample was first prepared, containing only the receptor ***E*-1** (50 µM in H_2O), and illuminated with 620 nm, to establish the maximum content of Z-isomer (>95%). Subsequently, 1 equivalent of H-*L*-Lys-OMe x 2HCl was added to the solution and the sample was immediately illuminated with the 400 nm emission wavelength LED mentioned above (Fig.S5 – right).

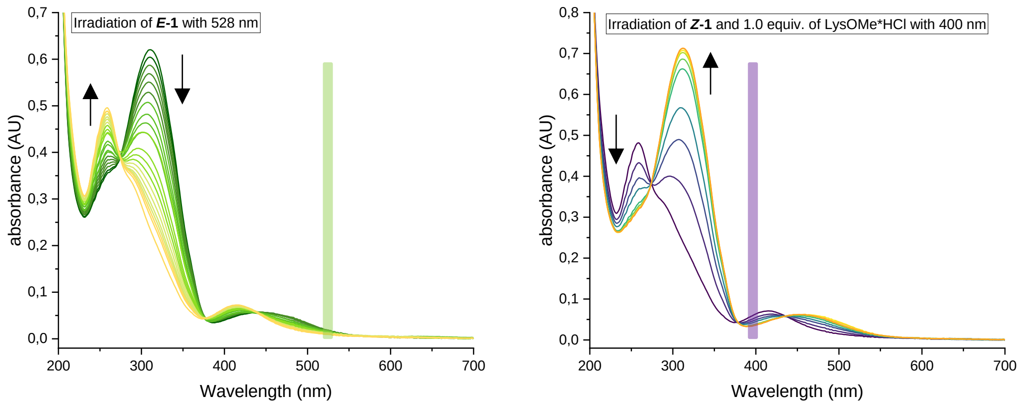

***Fig. S5** (left) UV-Vis absorption spectra of **E-1** in H_2O at a concentration of 50 µM and under continuous illumination with 528 nm, as indicated by the green bar; (right) UV-Vis absorption spectra of **Z-1** with an*

additional 1.0 equiv. of H-L-LysOMe x 2HCl at 50 µM concentration in H_2O under continuous illumination with 400 nm, as indicated by the violet bar.

Since continuous irradiation well after the PSS is reached will eventually lead to a measured reaction quantum yield of $\phi_{t\rightarrow\infty} = 0$, only samples were taken into account, which still lie in the linear regime of the reaction progress, which ensures that ϕ_{max} can be determined accurately.

Having obtained the product concentrations for the defined illumination intervals, they were plugged into equation 3.

$$\Phi = \frac{N_{prod}}{N_{ph,abs}} = N_A hc \frac{c_{prod}V}{P_{abs}\Delta t \lambda_{LED}} \quad (3)$$

Where Φ signifies the reaction quantum yield, N_A is Avogadro's constant, h is Planck's constant, c denotes the speed of light, c_{prod} stands for the product concentration formed by reaction progress, V is the sample volume, P_{abs} is the absorbed light power, Δt denotes the respective time interval and λ_{LED} stands for the nominal emission wavelength of the respective LED used for the experiment.

The values obtained were finally averaged and their standard deviation was calculated (Fig.S6).

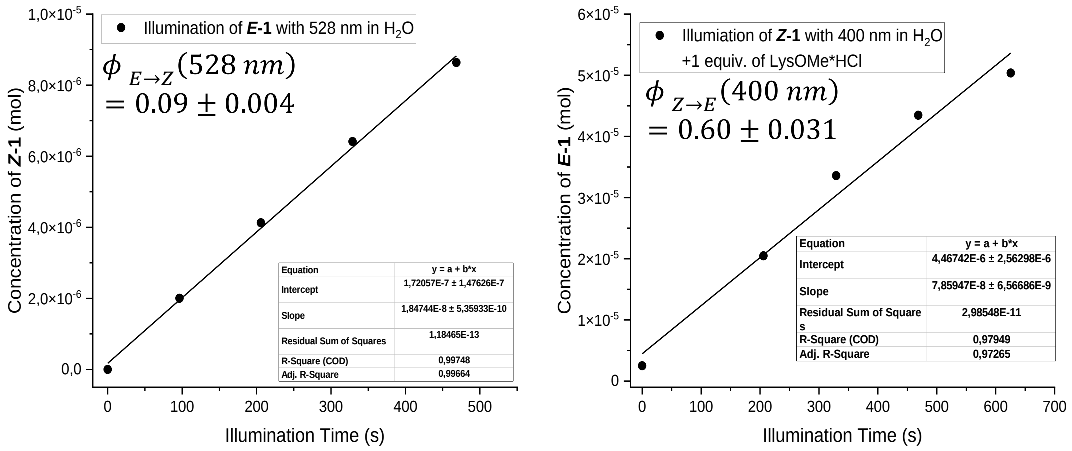

***Fig. S6** Concentration of the respective isomer plotted against the illumination time for a 50 µM solution of **E-1** (left) and **Z-1** with 1.0 equiv. of H-L-LysOMe x 2HCl (right), in H_2O. The quantum yields are stated for the respective illumination wavelengths and provided with their standard deviations.*

Supplementary Note 4.

Fatigue resistance measurements.

A 100 µM solution of ***E*-1** in deionized water was irradiated in an alternating fashion with light of 523 nm wavelength for 60 s, and then of 410 nm wavelength for 10 s. A total of 20 switching cycles were performed, after each irradiation step an absorption spectrum was recorded and the absorbance at 312 nm was plotted against the illumination step to depict the fatigue resistance of receptor **1**. The lower absorbance for the non-illuminated solution is the result of an *E*-/*Z*-mixture being present in the original sample; the resulting PSS at 410 nm illumination is higher in the *E*-isomer, thus absorbance is higher at the indicated wavelength.

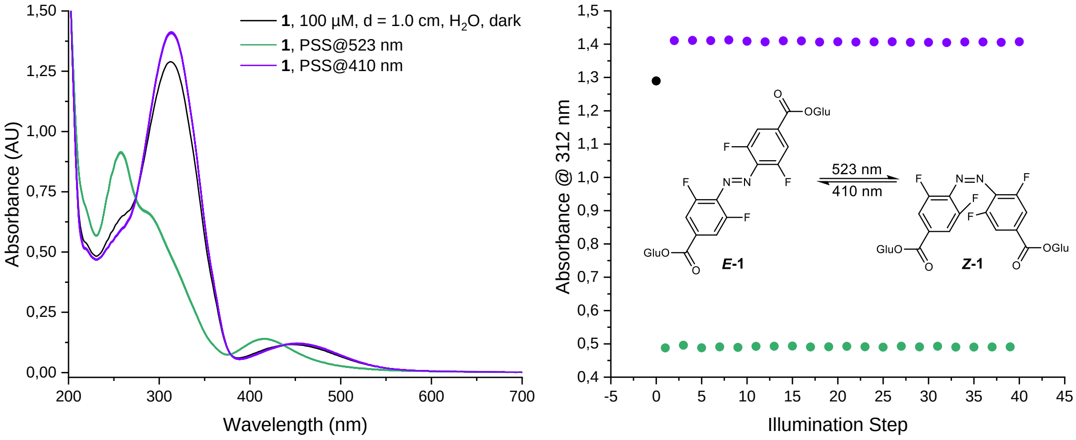

Fig. S7 *(left) UV-Vis absorption spectra of* ***1*** *in* H_2O *at a concentration of 100 µM prior to illumination (black curve) and after successive illuminations with 523 nm light (green curves) and 410 nm light (violet curves), for a total of 20 switching cycles; (right) Absorbance of the sample at 312 nm, plotted against each individual illumination step.*

Supplementary Note 5.

Binding mode analysis – NMR experiments

H-*L*-Lys-OMe x 2HCl @ Receptor 1

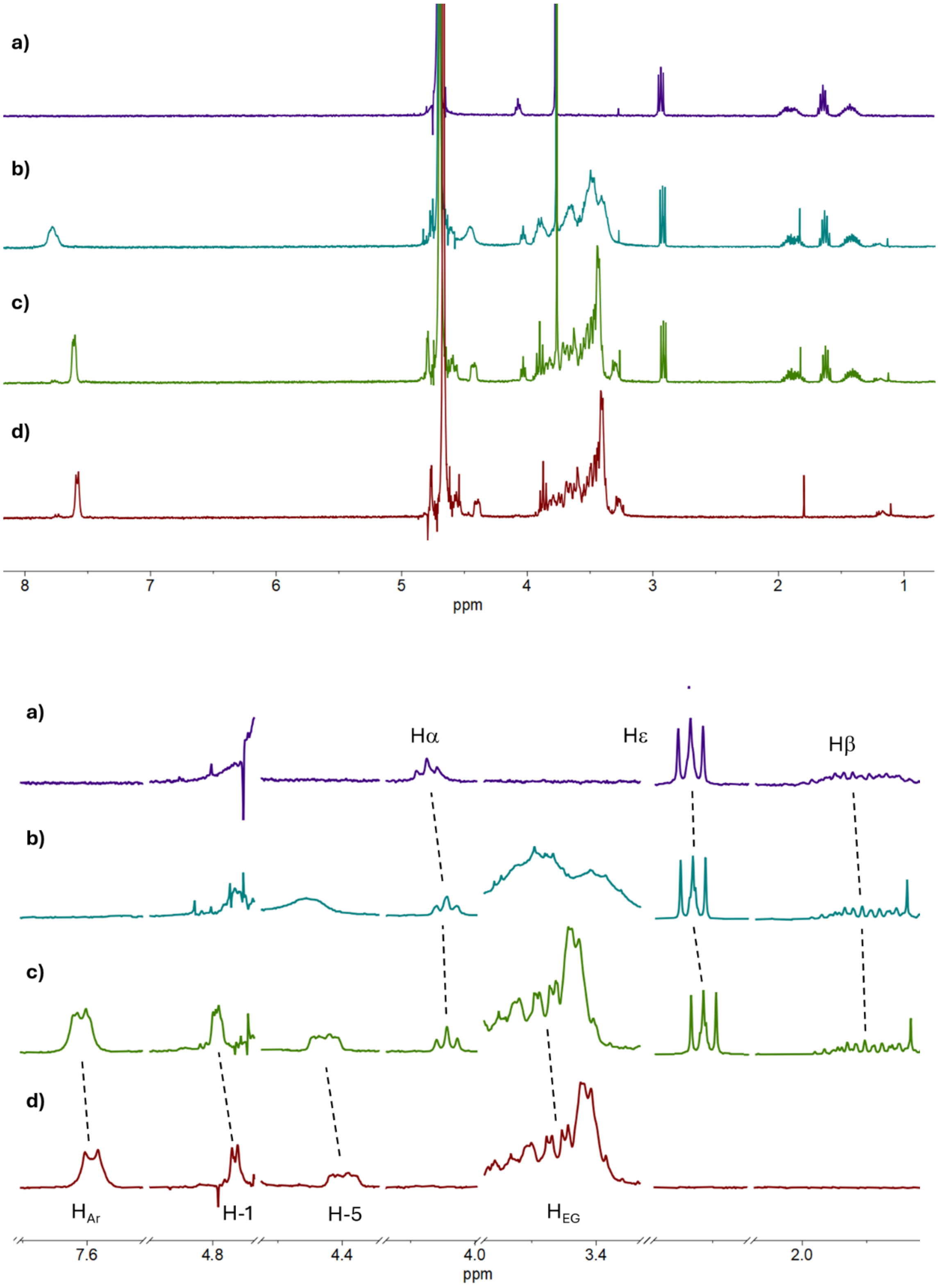

Fig. S8 *Stacked plot of* [1]*H NMR spectra in* D_2O*: a) H-L-Lys-OCH*$_3$ *x 2HCl; b)* ***E-1*** *+ H-L-Lys-OCH*$_3$ *x 2HCl = 1:1; c)* ***Z-1*** *+ H-L-Lys-OCH*$_3$ *x 2HCl = 1:1; d)* ***Z-1****. Top – full spectra, bottom – enhanced regions were significant changes of signal shifts were observed (the shifts are indicated with dashed arrows).*

H-*L*-Orn-OMe x 2HCl @ Receptor 2

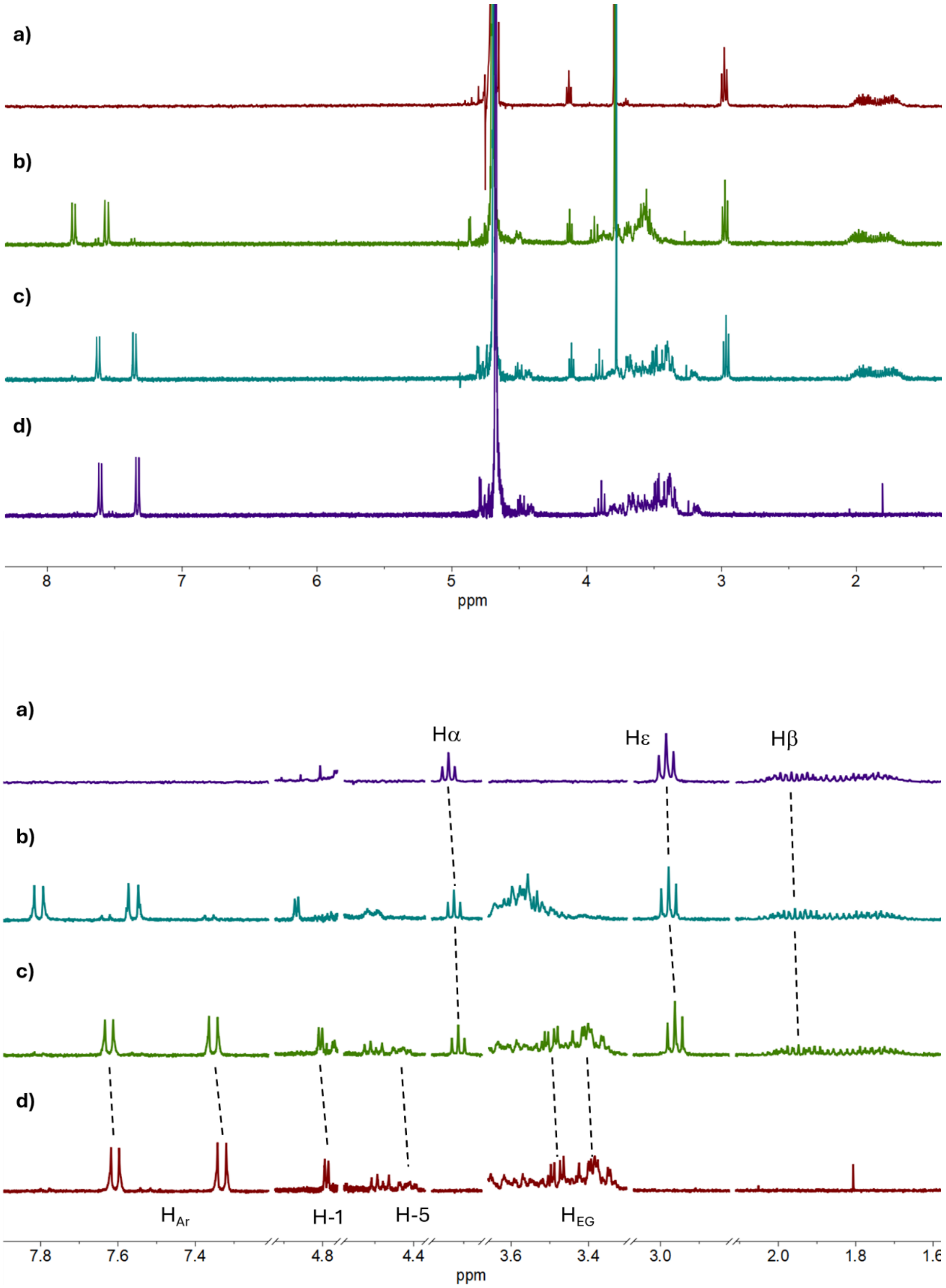

Fig. S9 *Stacked plot of* [1]*H NMR spectra in* D_2O*: a) H-L-Orn-*OCH_3 *x 2HCl; b)* ***E-2*** *+ H-L-Orn-*OCH_3 *x 2HCl = 1:1; c)* ***Z-2*** *+ H-L-Orn-*OCH_3 *x 2HCl = 1:1; d)* ***Z-2****. Top – full spectra, bottom – enhanced regions were significant changes of signal shifts were observed (the shifts are indicated with dashed arrows).*

H-*L*-Phe-OMe x HCl @ Receptor 2

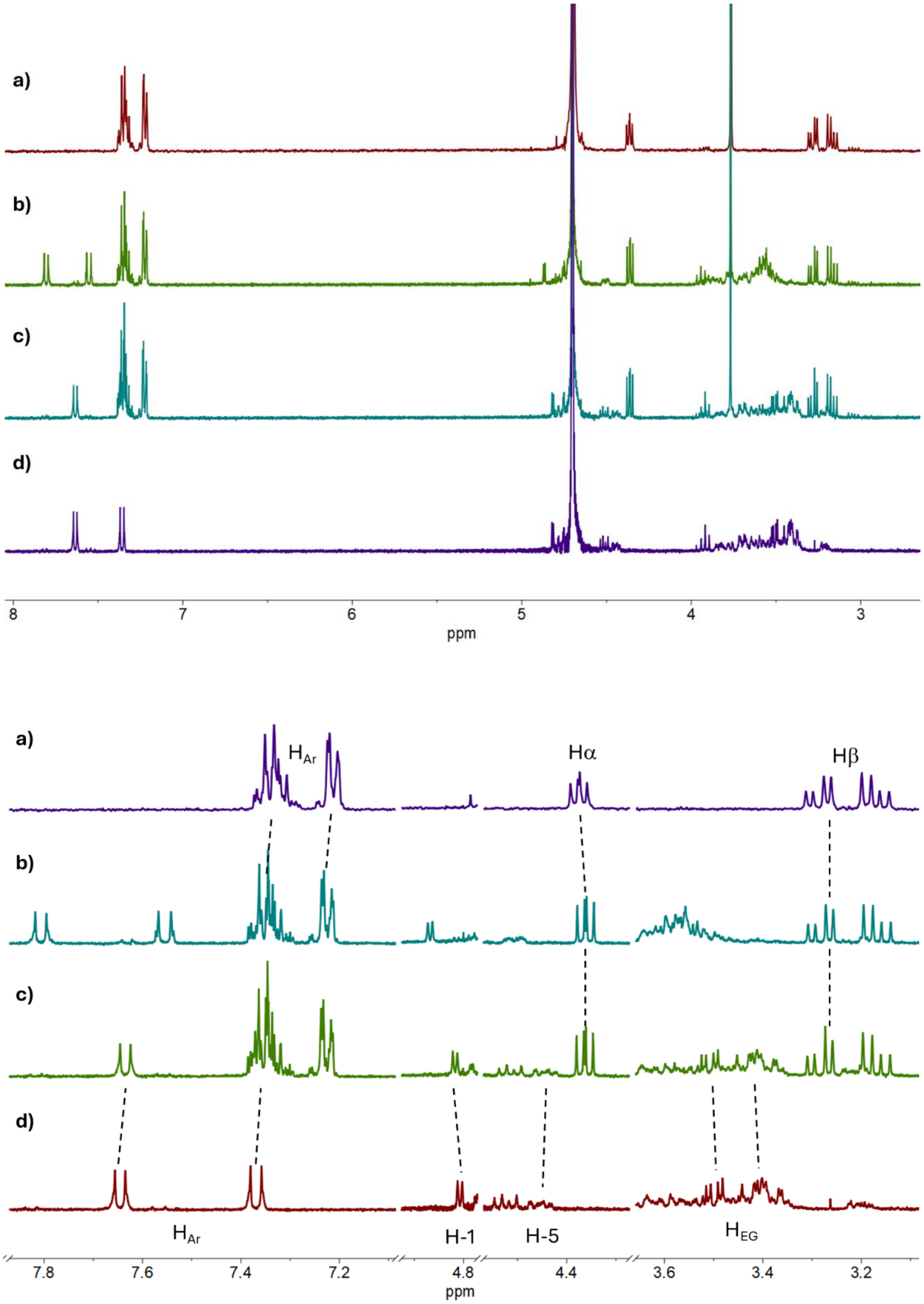

Fig. S10 *Stacked plot of ^{1}H NMR spectra in D_2O: a) H-L-Phe-OCH_3 x 2HCl; b)* ***E-2*** *+ H-L-Phe-OCH_3 x 2HCl = 1:1; c)* ***Z-2*** *+ H-L-Phe-OCH_3 x 2HCl = 1:1; d)* ***Z-2****. Top – full spectra, bottom – enhanced regions were significant changes of signal shifts were observed (the shifts are indicated with dashed arrows).*

Supplementary Note 6.

Computational modelling.

6.1. Modelling of receptor 1.

3D structures of the *Z*- and *E*-configured receptor were preoptimized using GFN2-xTB in the gas-phase employing xTB version 6.6.0.[3,4] Conformer-rotamer ensemble sampling at the GFN2-xTB(ALPB(water)) level was performed using CREST version 2.12 through the interface of CREST with xTB.[3–7] Refinement of the conformer-rotamer ensembles was performed by re-ranking to the r^{2}SCAN-3c(CPCM(water)) level with calculation of thermostatistical contributions using GFN2-xTB(ALPB(water)).[3,4,7–10] For the re-ranking, CENSO version 1.2.0 (*Part*0-2) was used through the interface of CENSO with xTB and ORCA version 5.0.3.[11,12] The conformer-rotamer ensemble sorting settings employed with CENSO, cartesian coordinates of and information on the ensembles are provided as separate files.

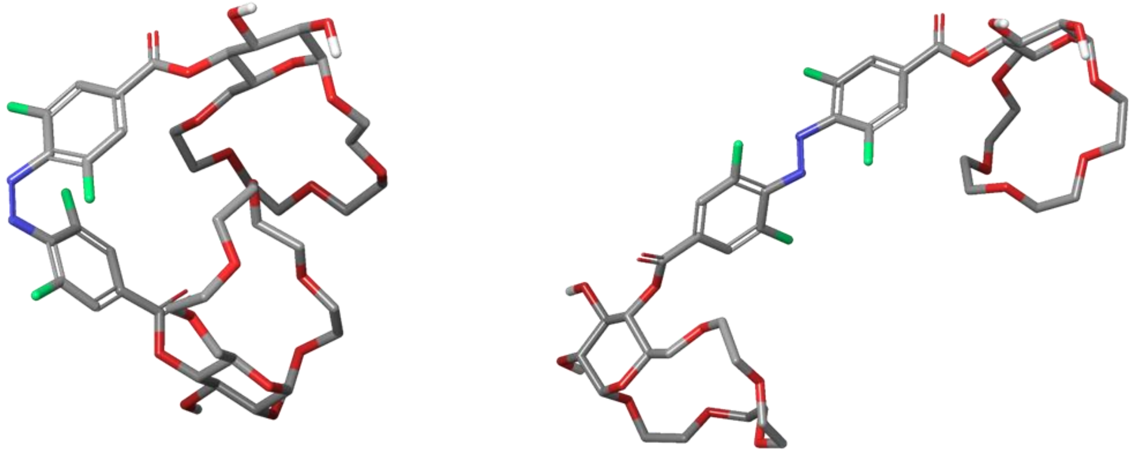

Fig. S11 *Lowest energy conformers of Z- (left) and E- (right) isomers of receptor* ***1*** *at the r^{2}SCAN-3c(CPCM(water))+GFN2-xTB(ALPB(water)) level.*

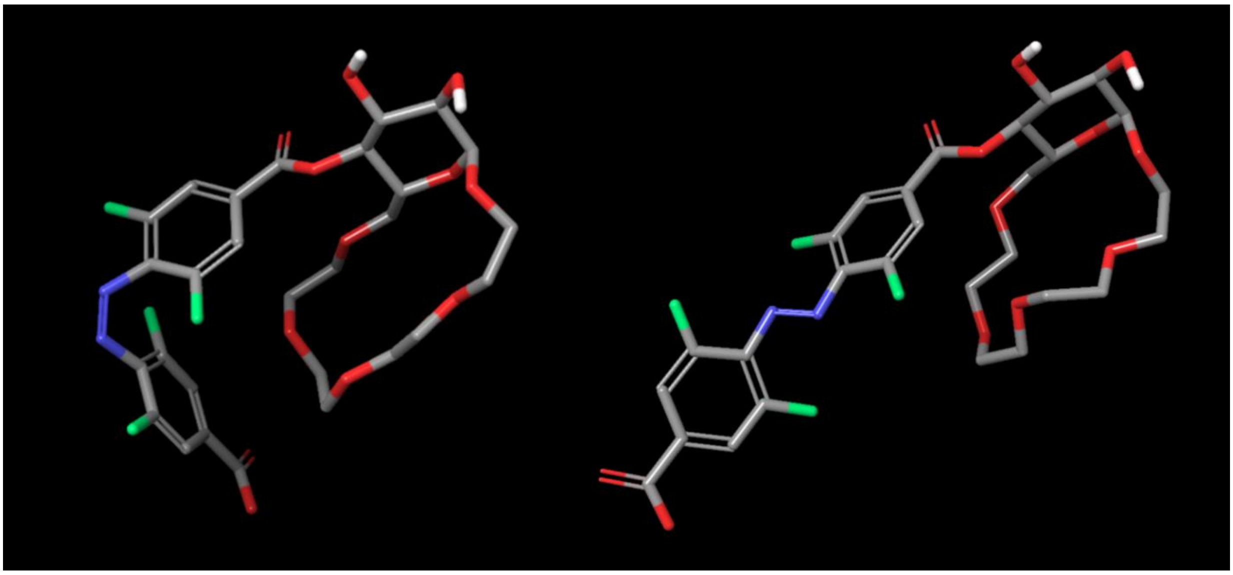

Fig. S12 *Lowest energy conformers of Z- (left) and E- (right) isomers of receptor* ***2*** *at the r^{2}SCAN-3c(CPCM(water))+GFN2-xTB(ALPB(water)) level.*

6.2. Modelling H-*L*-Lys-OMe bound to the receptor.

Initial 3D structures of H-*L*-Lys-OMe bound to the *Z*- or *E*-configured receptor were modelled using the lowest-energy conformers of each receptor configuration obtained using the workflow outlined above. These structures were preoptimized using GFN2-xTB(ALPB(water)) employing xTB version 6.6.0.[1,2] Conformer-rotamer ensemble sampling of the non-covalent complexes was performed using the NCI mode of CREST version 2.12 through the interface of CREST with xTB.[1–5] Conformer-rotamer ensembles were subsequently refined as described above. The conformer-rotamer ensemble sorting settings employed with CENSO and cartesian coordinates of and information on the ensembles are provided as separate files.

The lowest-energy conformers of the non-covalent complexes between the *Z*- or *E*-configured receptor and H-*L*-Lys-OMe are shown below. The smaller distance between the crown ethers in the *Z*-configured receptor might allow various conformers of the guest to interact with both crown ethers. The larger distance between the crown ethers in the *E*-configured receptor, contrary, appears to necessitate side chain dihedrals close to 180° for interaction with both crown ethers.

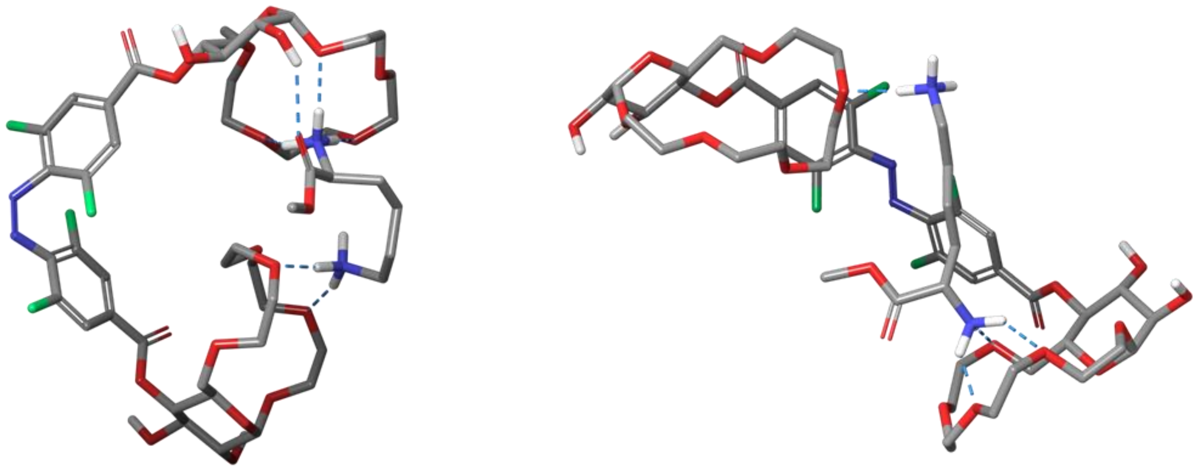

Fig. S13 *Lowest energy conformers of the complexes between H-L-Lys-OMe and the Z- (left) and E- (right) isomer of receptor **1** at the r²SCAN-3c(CPCM(water))+GFN2-xTB(ALPB(water)) level.*

Supplementary Note 7.

ITC measurements.

ITC experiments were carried out in a MicroCal PEAQ-ITC (Malvern), at 25°C, 750 rpm, high feedback and 10 µcal/s as reference power, using Milli-Q pure water or aqueous PBS buffer (pH 7.4) as a solvent.

The concentrations of the host and the guest solutions were 0.2 mM and 6 mM respectively. The guest solution was added in 1µL portions at 2 min intervals.

The data was analysed by fitting to a one set of sites model using the MicroCal PEAQ-ITC Control Software. Application of a two-binding-site model did not allow to obtain a fit for the titration data.

Summary of ITC results in H_2O:

guest	Z-1				E-1			
	K_D[a]	ΔG[b]	ΔH	-TΔS	K_D	ΔG	ΔH	-TΔS
L-Lys	0.71±0.19	-4.29	0.28	-4.57	2.16±0.58	-3.64	0.62	-4.28
L-Arg	0.73±0.12	-4.27	0.24	-4.51	1.81±0.41	-3.73	0.58	-4.31
L-Orn	0.64±0.07	-4.35	0.41	-4.76	1.82±0.25	-3.73	0.81	-4.54
L-Phe	0.94±0.26	-4.14	0.21	-4.35	1.20±0.36	-3.99	0.44	-4.43
L-Val	1.29±0.52	-3.94	0.13	-4.07	1.48±0.49	-3.86	0.23	-4.09
L-Ala	1.83±0.72	-3.73	0.05	-3.78	2.04±0.79	-3.66	0.11	-3.77
D-Lys	1.37±0.36	-3.90	0.57	-4.47	2.72±1.15	-3.50	0.44	-3.94
D-Arg	1.41±0.38	-3.88	0.39	-4.27	2.03±0.82	-3.66	0.66	-4.32
cadaverine	0.60±0.16	-4.39	-0.80	-3.59	1.65±0.51	-3.79	-0.47	-3.32
spermidine	0.70±0.06	-4.30	-0.67	-3.63	0.56±0.07	-4.43	-0.77	-3.67
spermine	0.57±0.08	-4.42	-0.46	-3.96	0.45±0.06	-4.56	-0.98	-3.58

a – K_D values are listed in mM; b – ΔG, ΔH and TΔS values are listed in kcal/mol

guest	Z-2				E-2			
	K_D[a]	ΔG[b]	ΔH	-TΔS	K_D	ΔG	ΔH	-TΔS
L-Lys	0.80±0.28	-4.22	0.18	-4.40	1.91±0.47	-3.71	0.42	-4.13
L-Arg	0.85±0.37	-4.18	0.42	-4.60	2.05±0.69	-3.66	0.66	-4.32
L-Orn	0.71±0.24	-4.29	0.41	-4.70	1.89±0.16	-3.70	0.88	-4.58
L-Phe	1.26±0.45	-3.95	0.35	-4.30	1.21±0.41	-3.98	0.42	-4.40
L-Val	1.62±0.55	-3.80	0.42	-4.22	1.51±0.59	-3.85	0.27	-4.12
L-Ala	1.84±0.82	-3.73	0.26	-3.99	2.10±0.52	-3.65	0.17	-3.82
D-Lys	1.34±0.34	-3.91	0.55	-4.41	2.39±0.65	-3.55	0.38	-3.93
D-Arg	1.28±0.49	-3.94	0.71	-4.65	2.55±0.81	-3.53	0.96	-4.47
cadaverine	0.85±0.23	-4.18	-0.85	-3.33	2.00±0.53	-3.68	-0.11	-3.57
spermidine	0.67±0.07	-4.32	-0.65	-3.67	0.55±0.06	-4.44	-0.78	-3.66
spermine	0.55±0.05	-4.44	-0.96	-3.48	0.46±0.05	-4.55	-1.13	-3.42

a – K_D values are listed in mM; b – ΔG, ΔH and TΔS values are listed in kcal/mol

Summary of ITC results in PBS:

guest	Z-1				E-1			
	K_D[a]	ΔG[b]	ΔH	-TΔS	K_D	ΔG	ΔH	-TΔS
L-Lys	1.32±0.55	-3.91	0.2	-4.11	5.32±2.77	-3.10	0.45	-3.55
L-Arg	1.28±0.34	-3.94	0.11	-4.05	4.52±2.35	-3.19	0.55	-3.74
L-Orn	1.63±0.56	-3.80	0.21	-4.01	5.68±2.23	-3.06	0.44	-3.50

a – K_D values are listed in mM; b – ΔG, ΔH and TΔS values are listed in kcal/mol

guest	Z-2				E-2			
	K_D[a]	ΔG[b]	ΔH	-TΔS	K_D	ΔG	ΔH	-TΔS
L-Lys	1.26±0.48	-3.95	0.18	-4.13	3.97±1.79	-3.27	0.31	-3.58
L-Arg	1.42±0.55	-3.89	0.25	-4.14	3.89±1.63	-3.28	0.43	-3.71
L-Orn	1.19±0.38	-3.98	0.32	-4.30	3.85±1.86	-3.29	0.61	-3.90

a – K_D values are listed in mM; b – ΔG, ΔH and TΔS values are listed in kcal/mol

Raw calorimetry data and fitted curves:

H-L-Lys-OMe x 2HCl to Z-1:

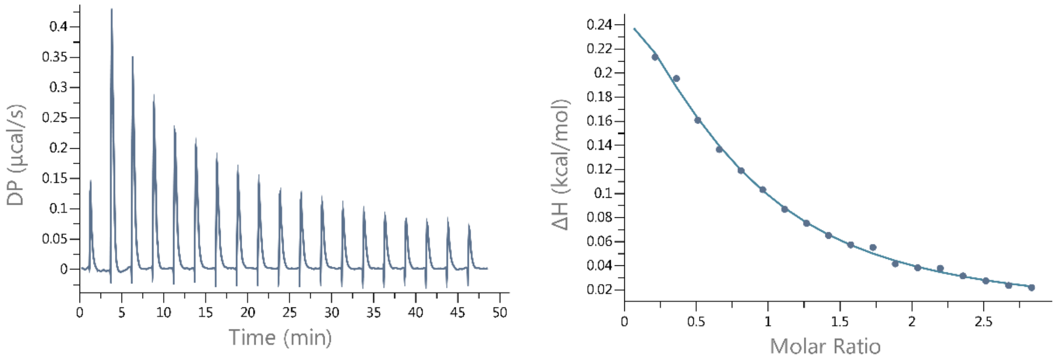

H-L-Lys-OMe x 2HCl to E-1:

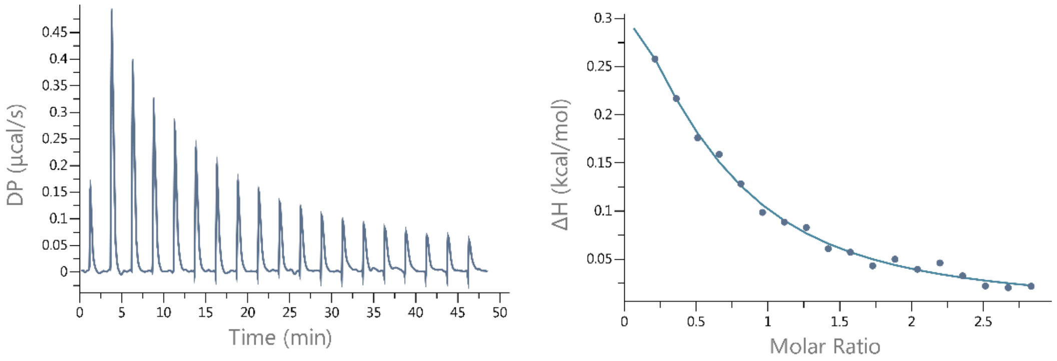

H-L-Lys-OMe x 2HCl to Z-2:

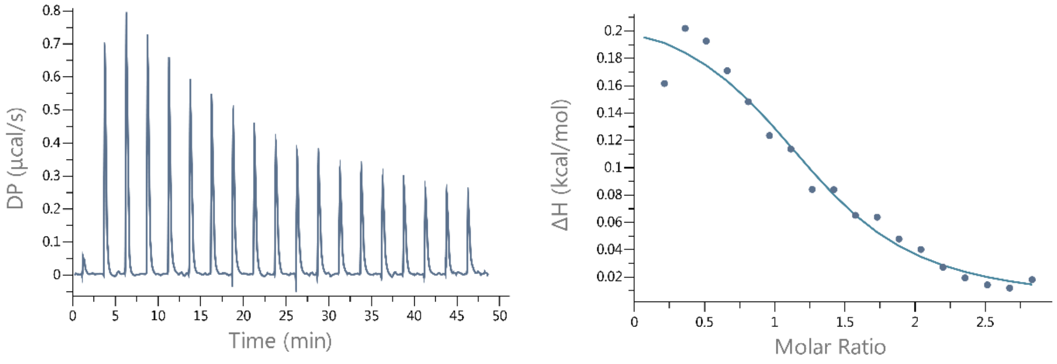

H-L-Lys-OMe x 2HCl to E-2:

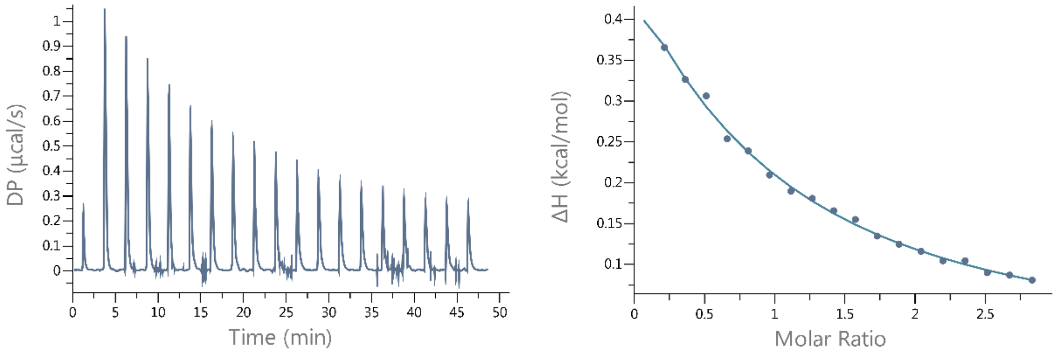

H-L-Arg-OMe x 2HCl to Z-1:

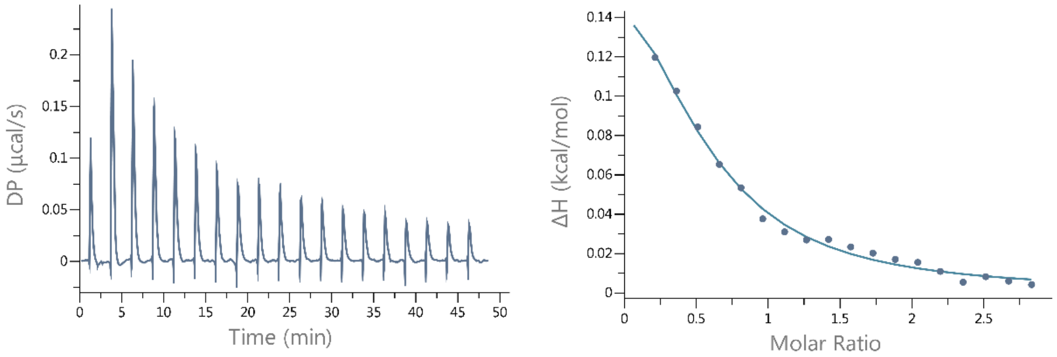

H-L-Arg-OMe x 2HCl to E-1:

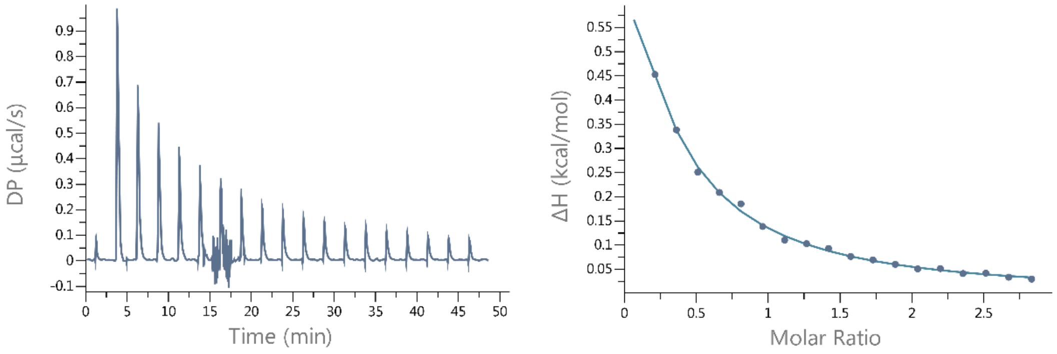

H-L-Arg-OMe x 2HCl to Z-2:

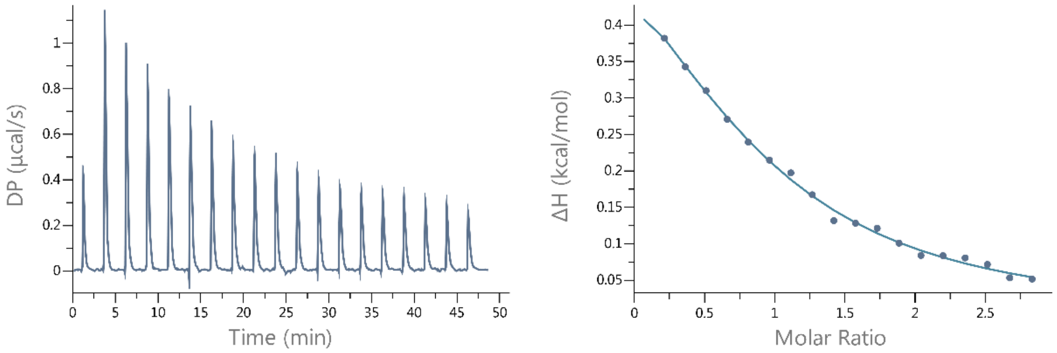

H-L-Arg-OMe x 2HCl to E-2:

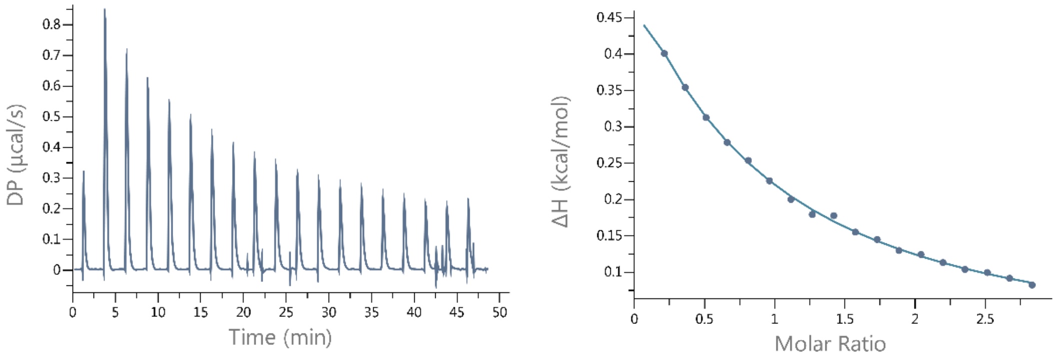

H-L-Orn-OMe x 2HCl to Z-1:

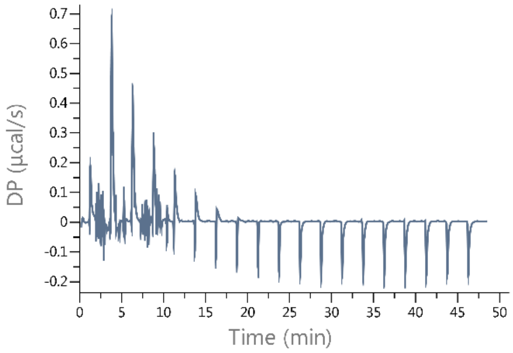

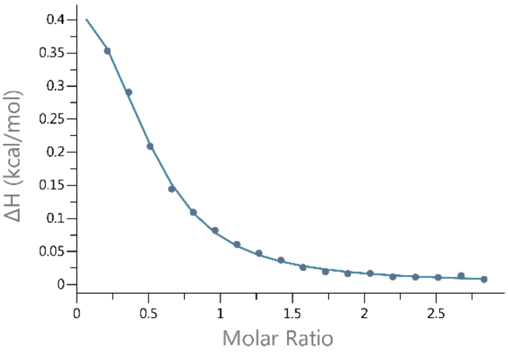

H-L-Orn-OMe x 2HCl to E-1:

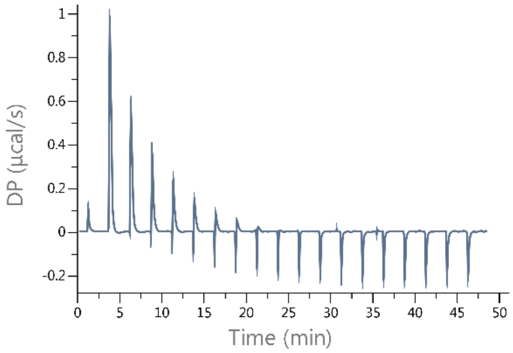

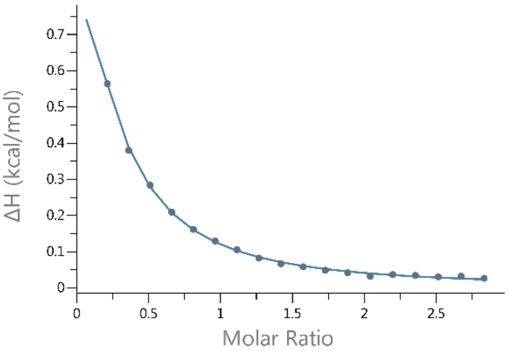

H-L-Orn-OMe x 2HCl to Z-2:

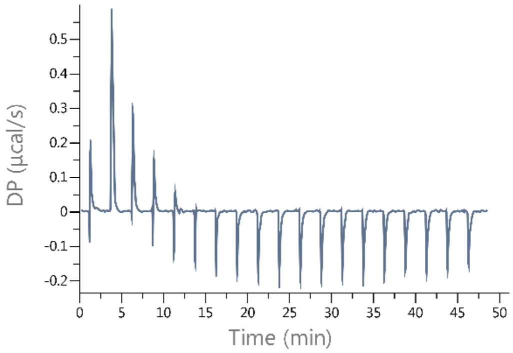

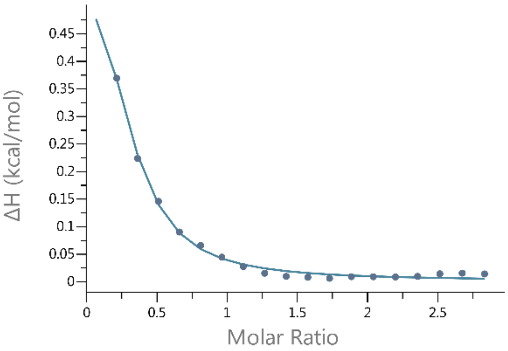

H-L-Orn-OMe x 2HCl to E-2:

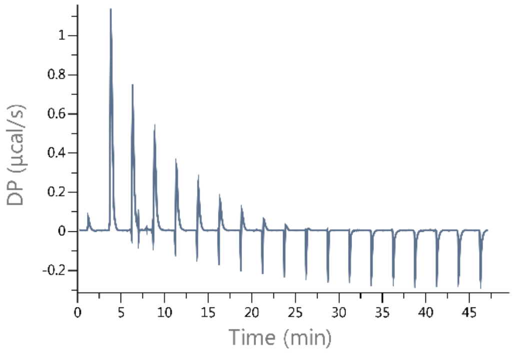

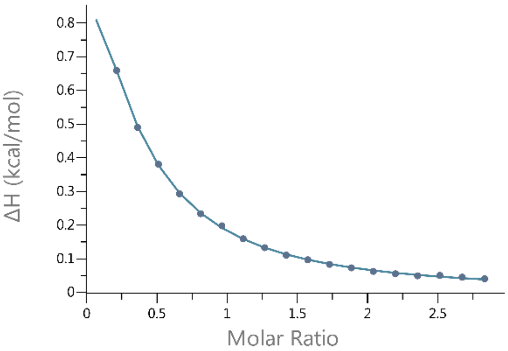

H-L-Phe-OMe x HCl to Z-1:

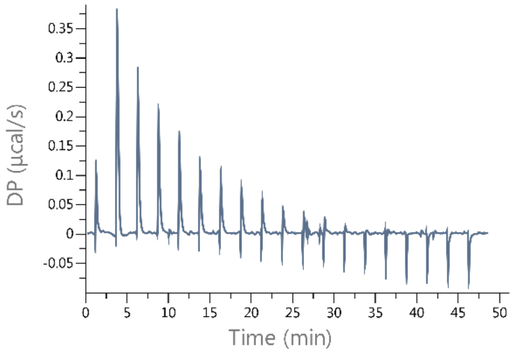

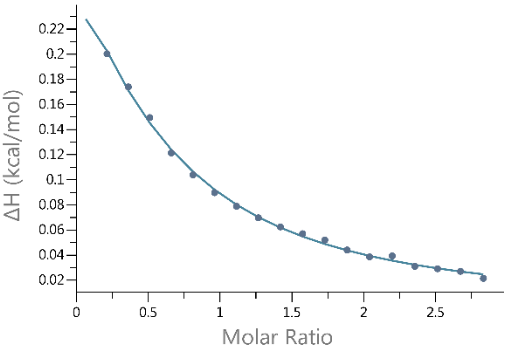

H-L-Phe-OMe x HCl to E-1:

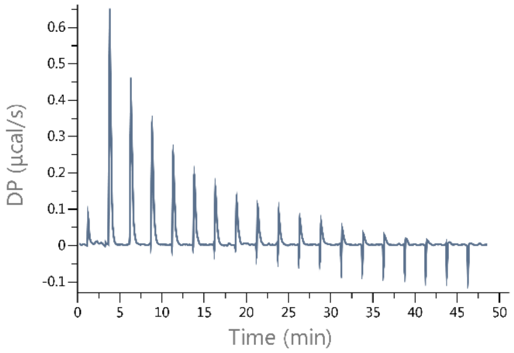

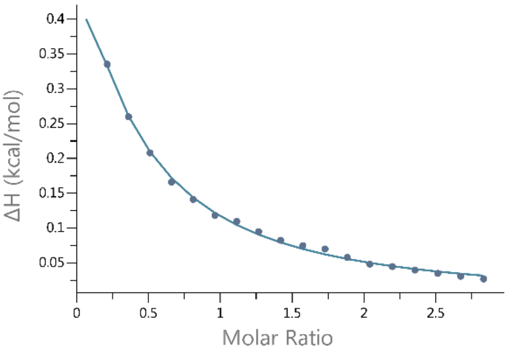

H-L-Phe-OMe x HCl to Z-2:

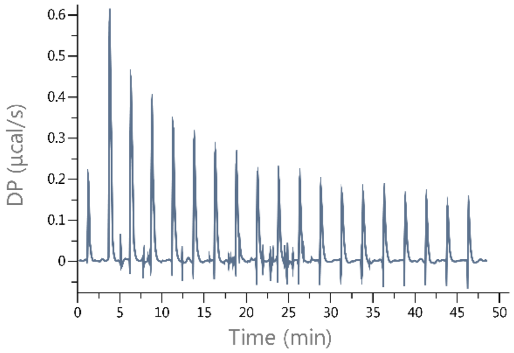

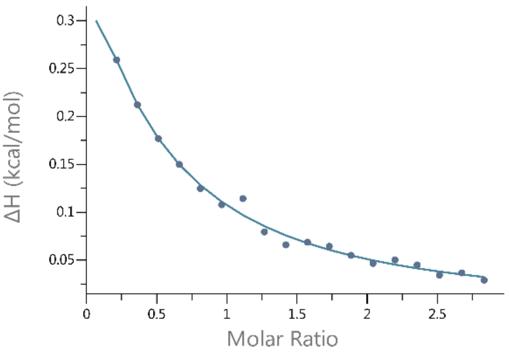

H-L-Phe-OMe x HCl to E-2:

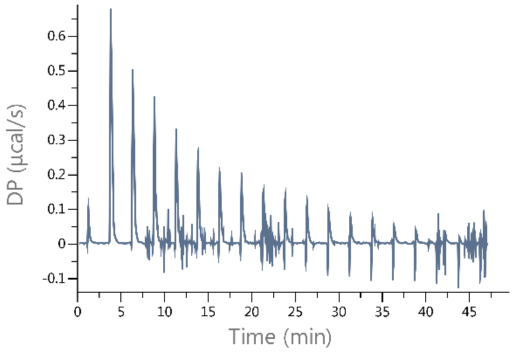

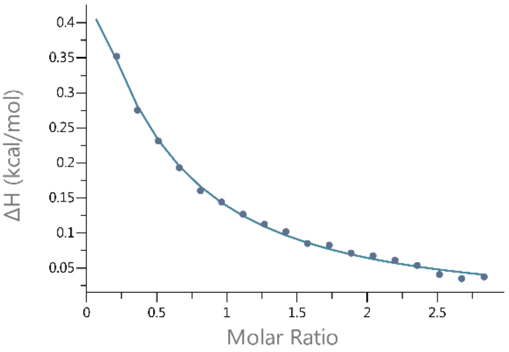

H-L-Val-OMe x HCl to Z-1:

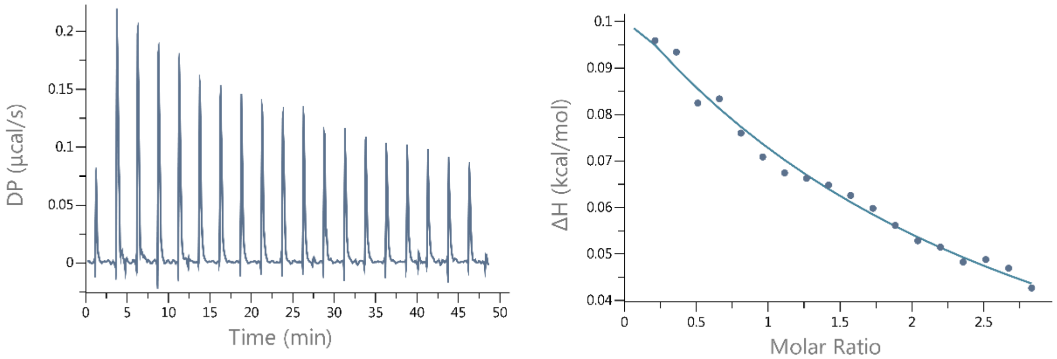

H-L-Val-OMe x HCl to E-1:

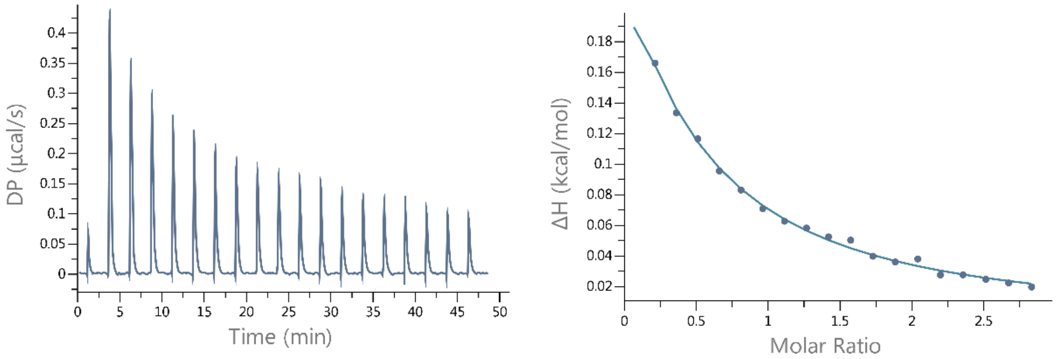

H-L-Val-OMe x HCl to Z-2:

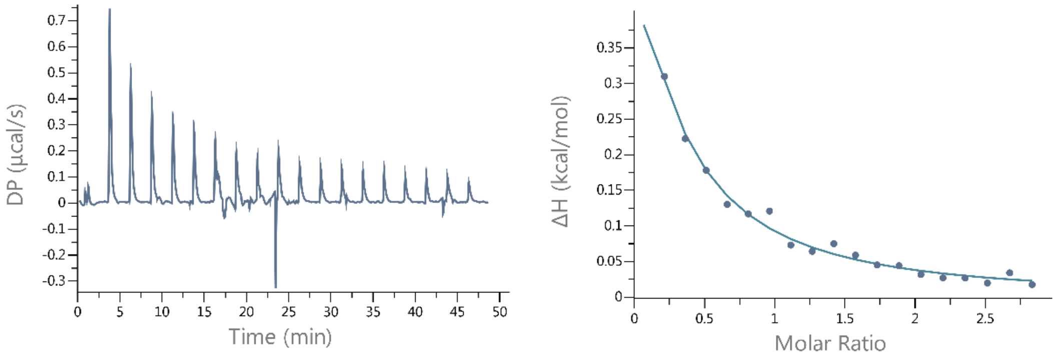

H-L-Val-OMe x HCl to E-2:

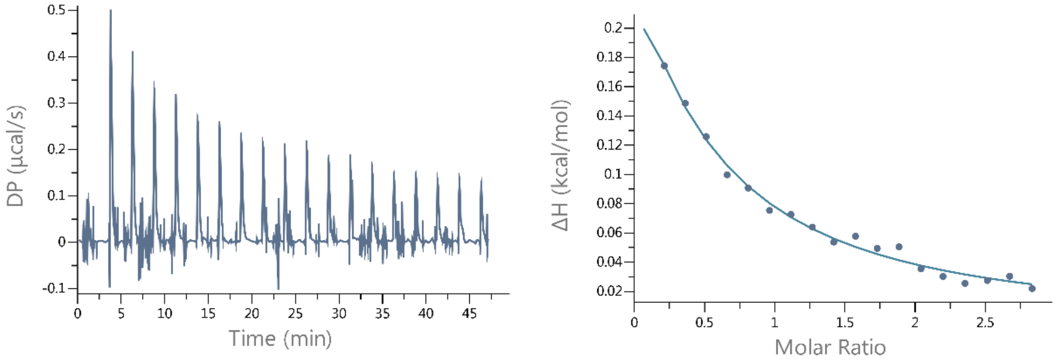

H-L-Ala-OMe x HCl to Z-1:

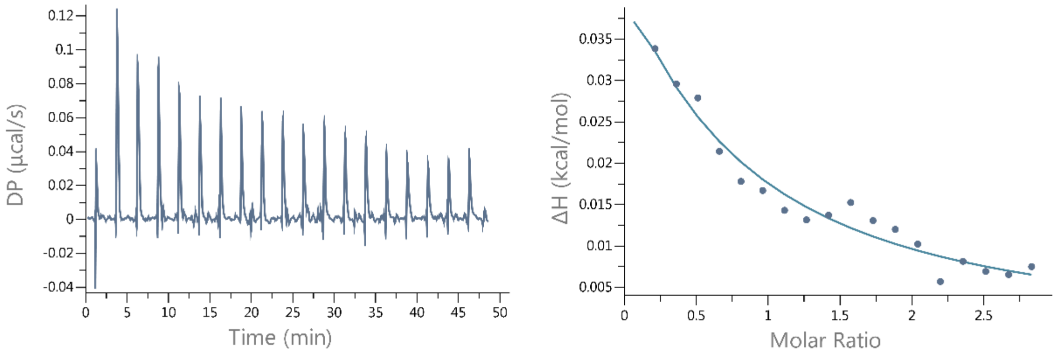

H-L-Ala-OMe x HCl to E-1:

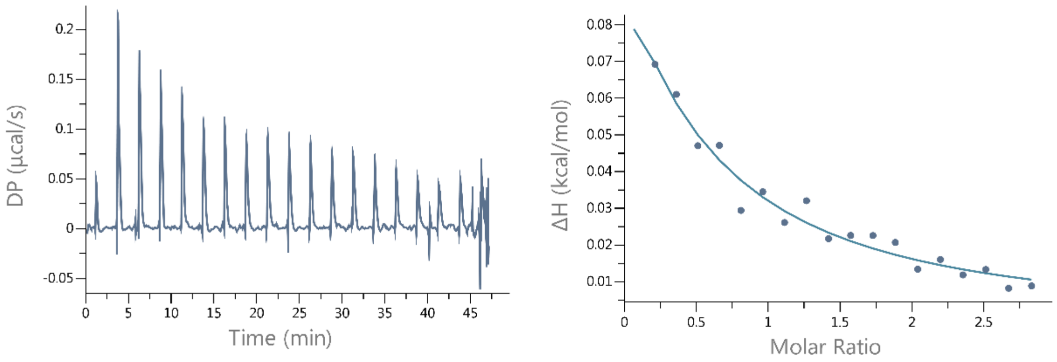

H-L-Ala-OMe x HCl to Z-2:

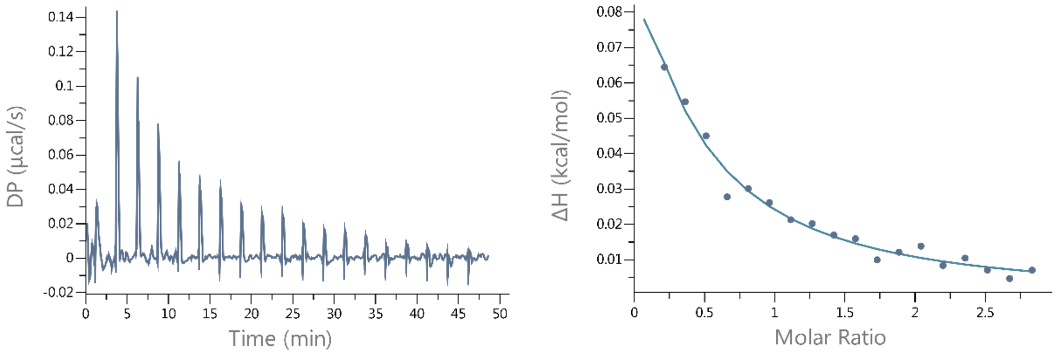

H-L-Ala-OMe x HCl to E-2:

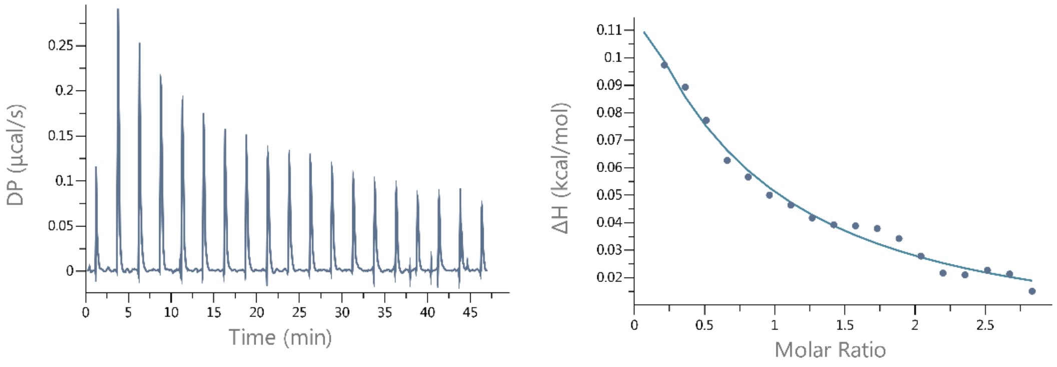

H-D-Lys-OMe x 2HCl to Z-1:

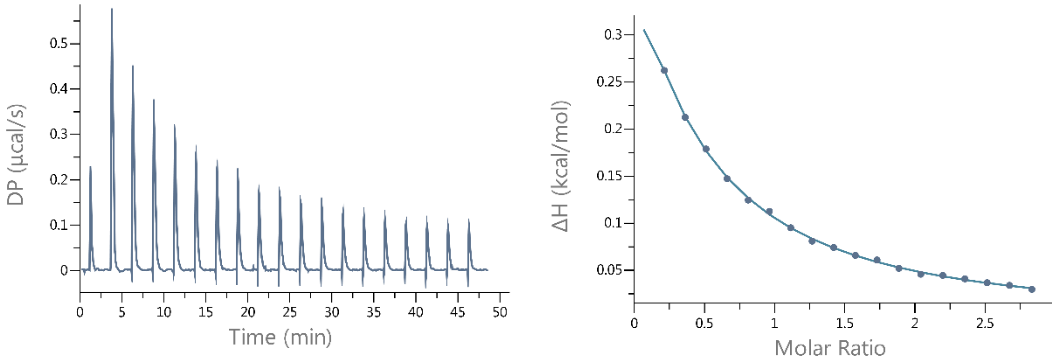

H-D-Lys-OMe x 2HCl to E-1:

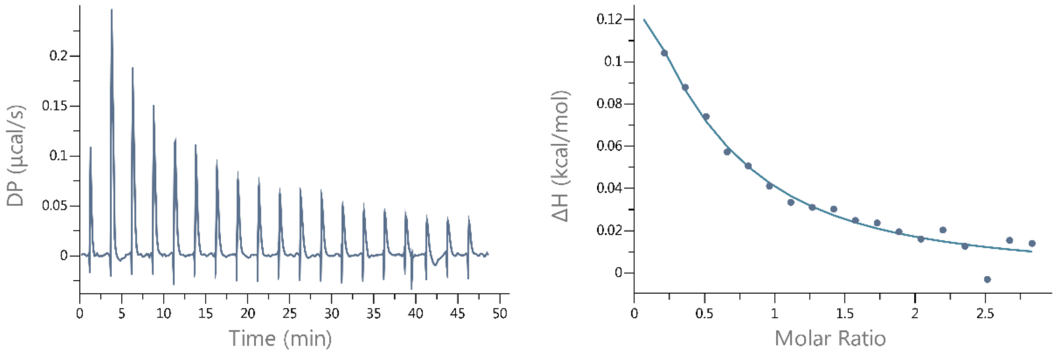

H-D-Lys-OMe x 2HCl to Z-2:

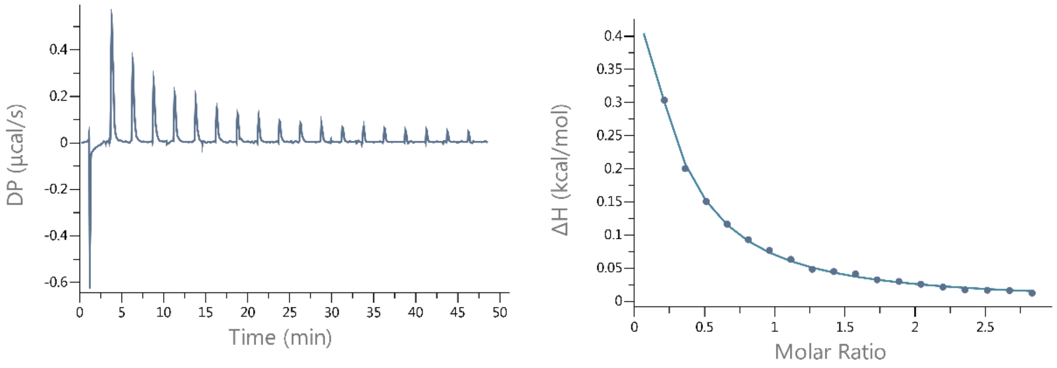

H-D-Lys-OMe x 2HCl to E-2:

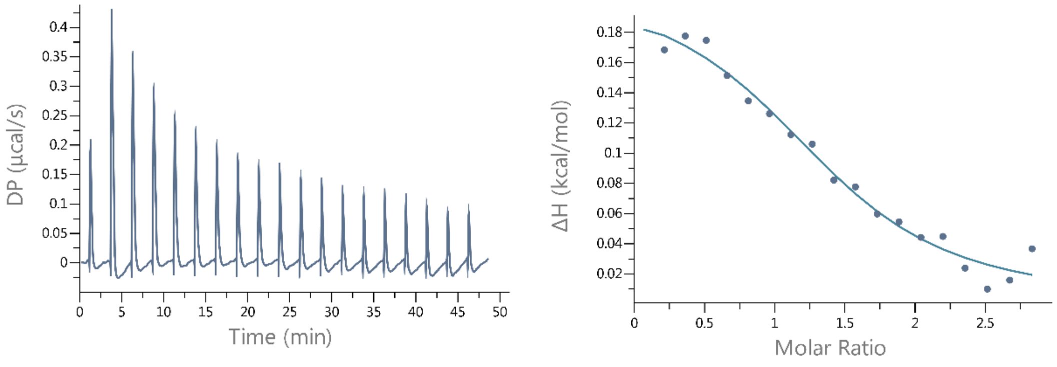

H-D-Arg-OMe x 2HCl to Z-1:

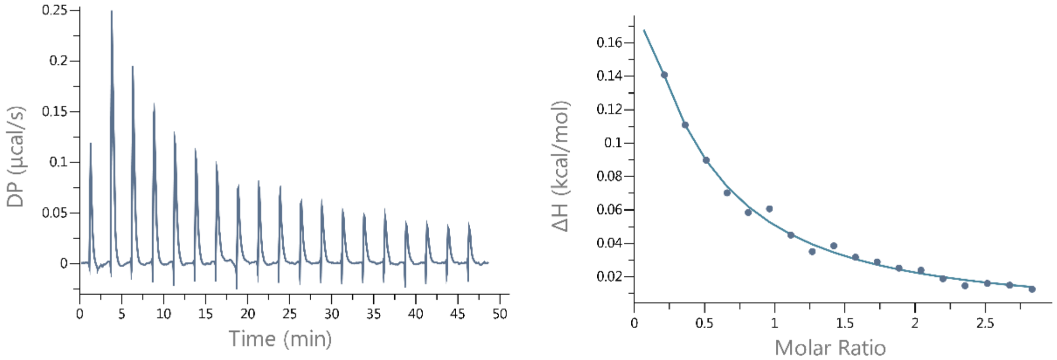

H-D-Arg-OMe x 2HCl to E-1:

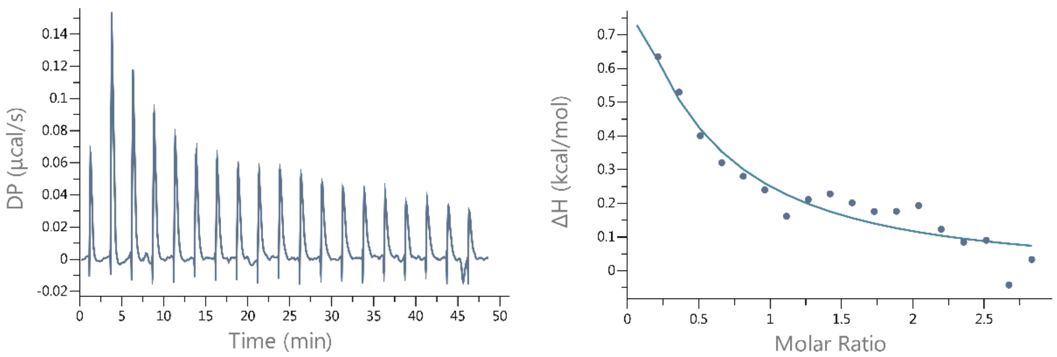

H-D-Arg-OMe x 2HCl to Z-2:

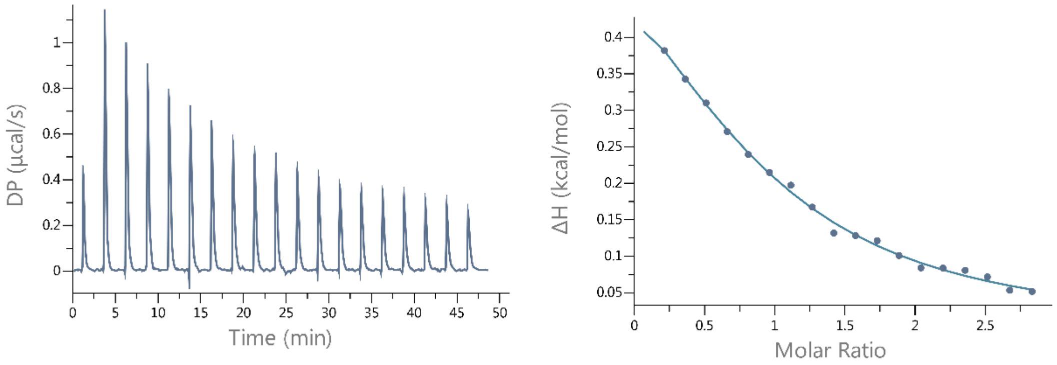

H-D-Arg-OMe x 2HCl to E-2:

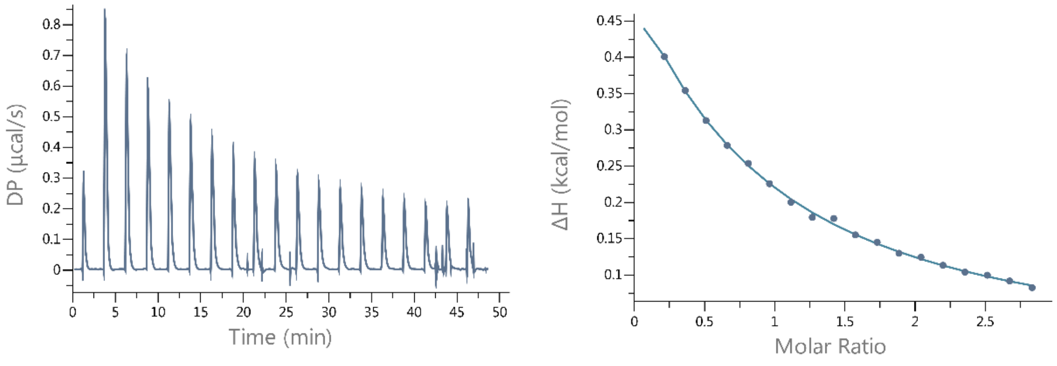

cadaverine to Z-1:

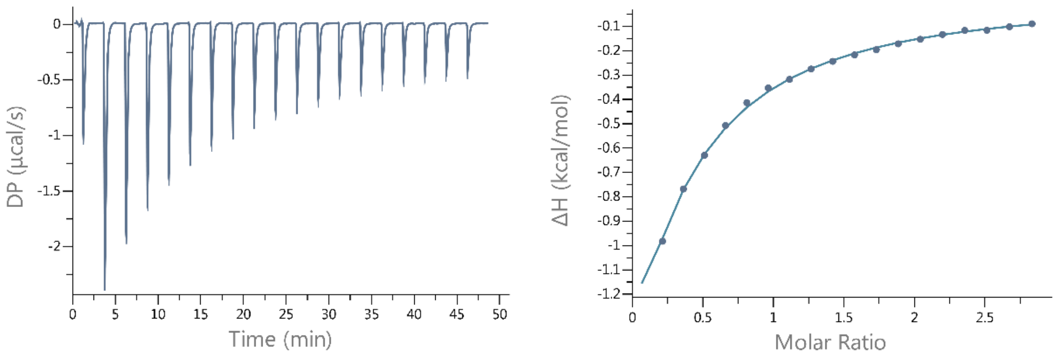

cadaverine to E-1:

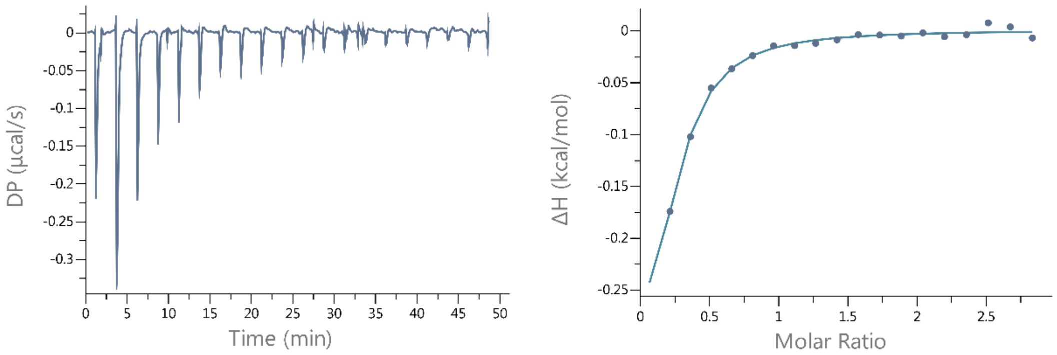

cadaverine to Z-2:

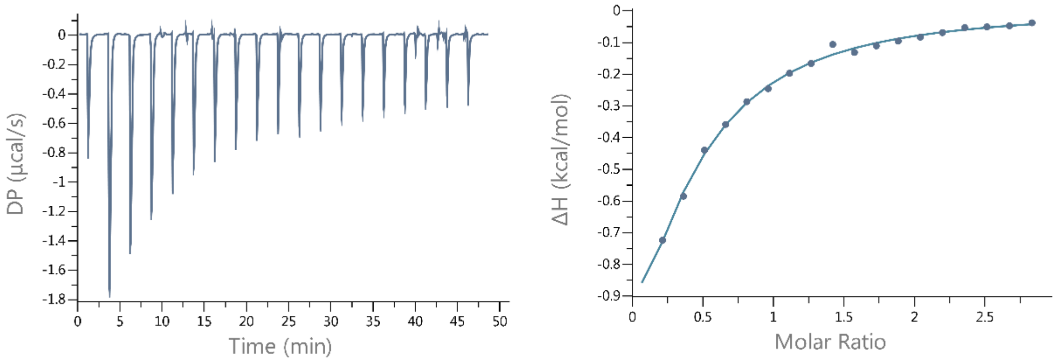

cadaverine to E-2:

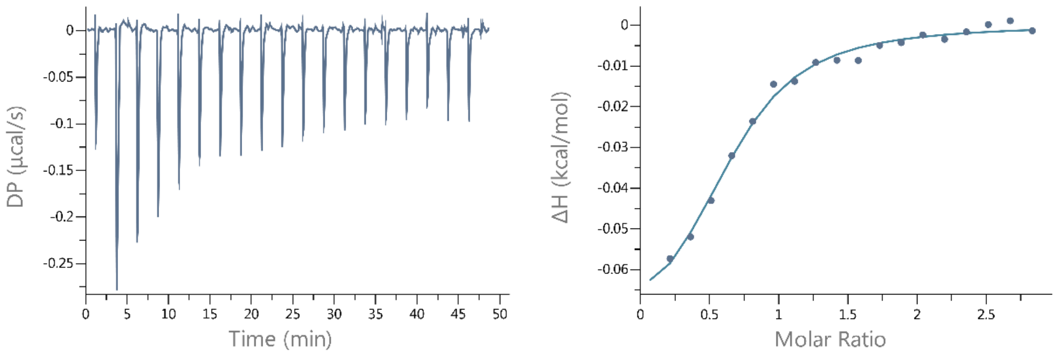

spermidine to Z-1:

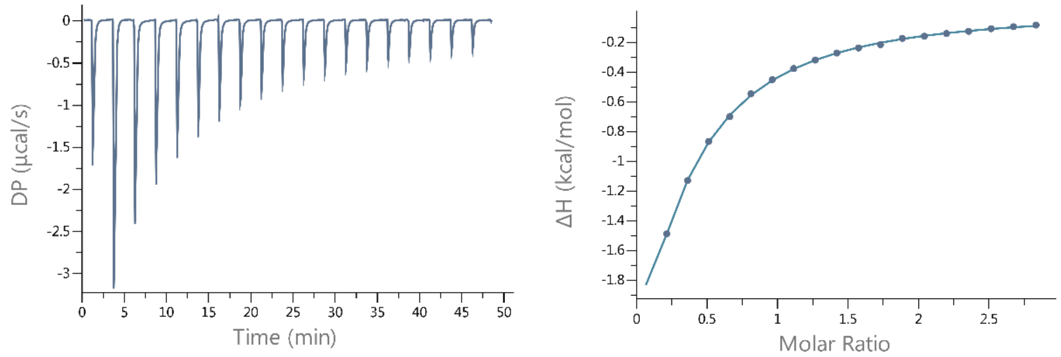

spermidine to E-1:

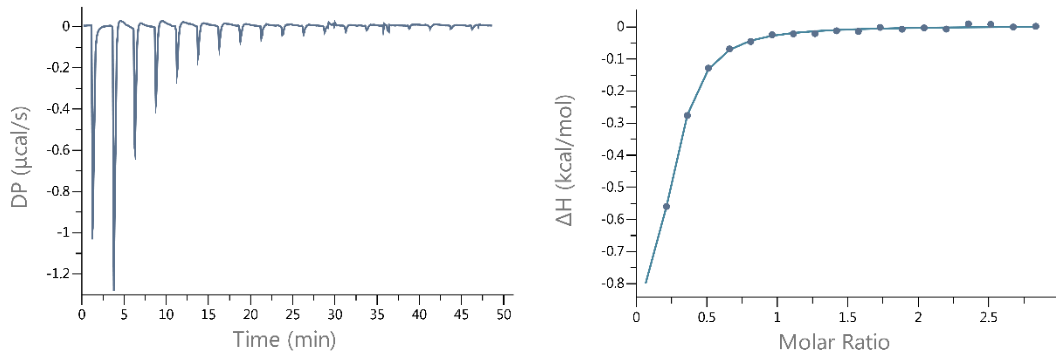

spermidine to Z-2:

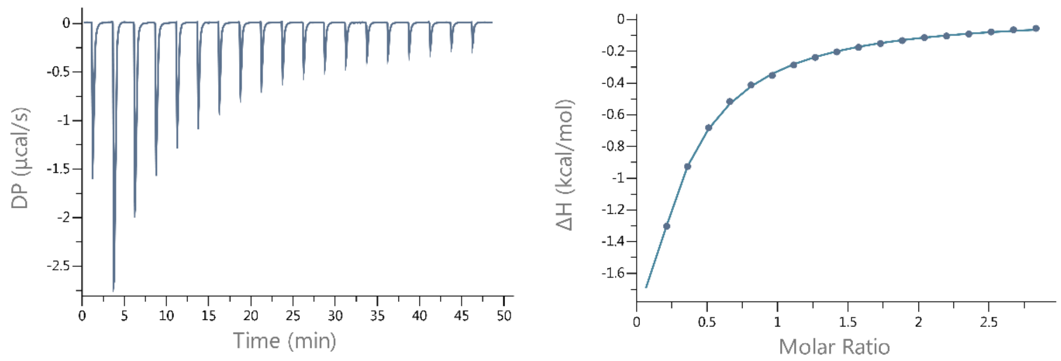

spermidine to E-2:

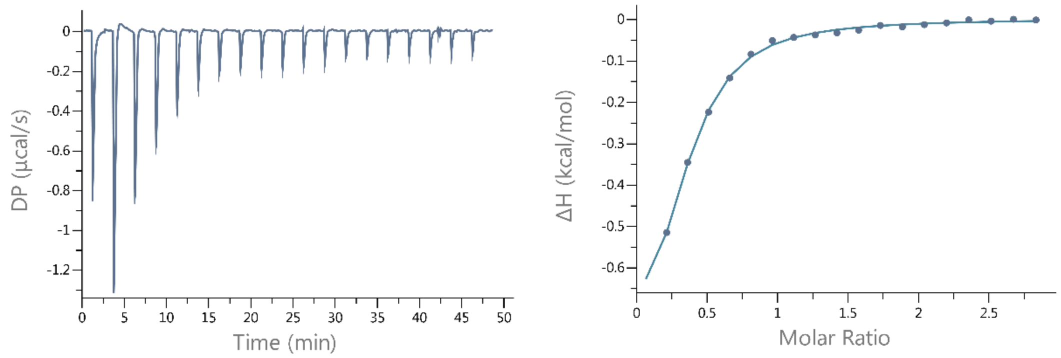

spermine to Z-1:

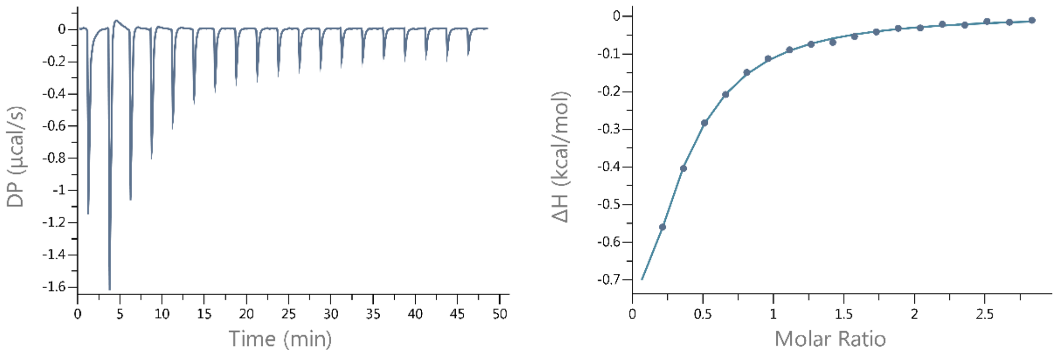

spermine to E-1:

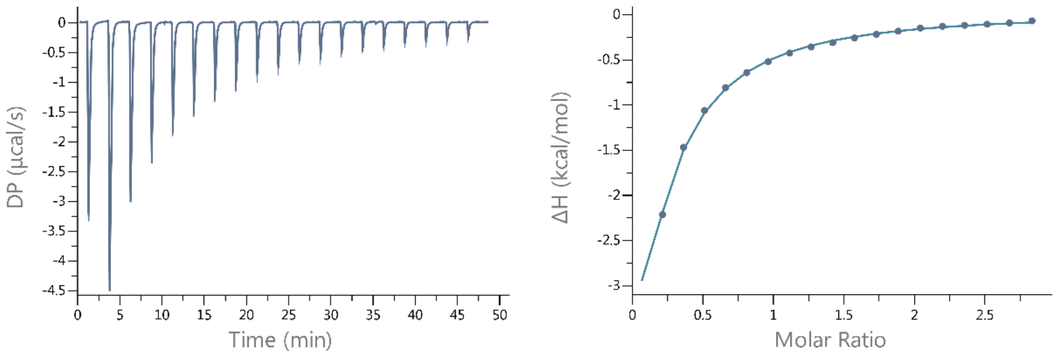

spermine to Z-2:

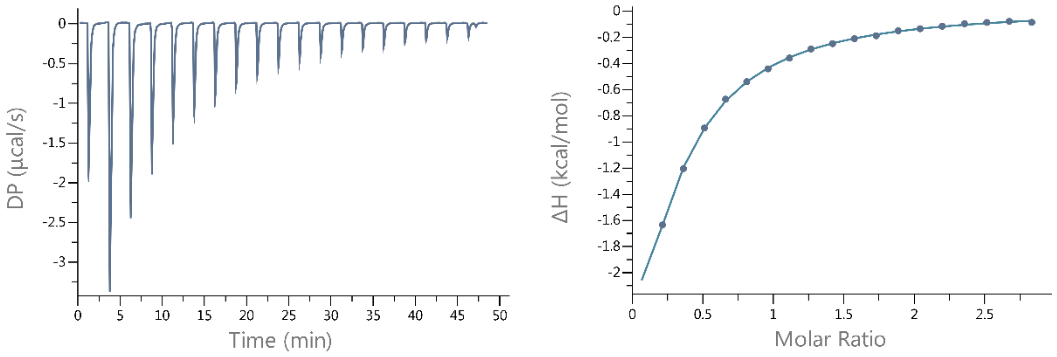

spermine to E-2:

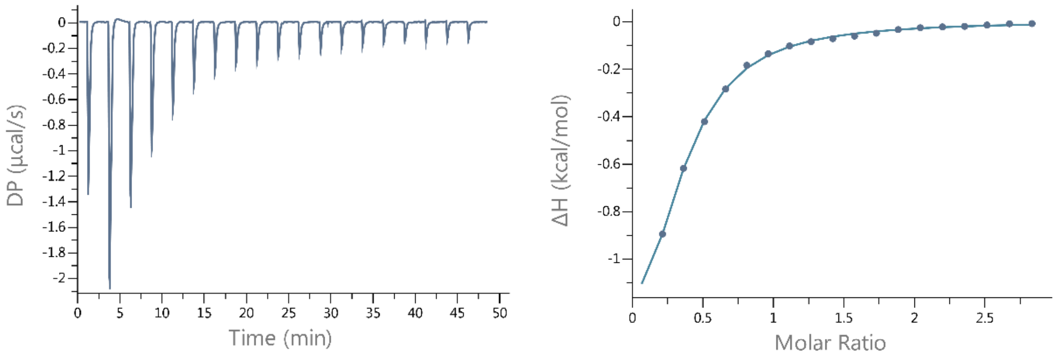

H-L-Lys-OMe x 2HCl to Z-1 (PBS):

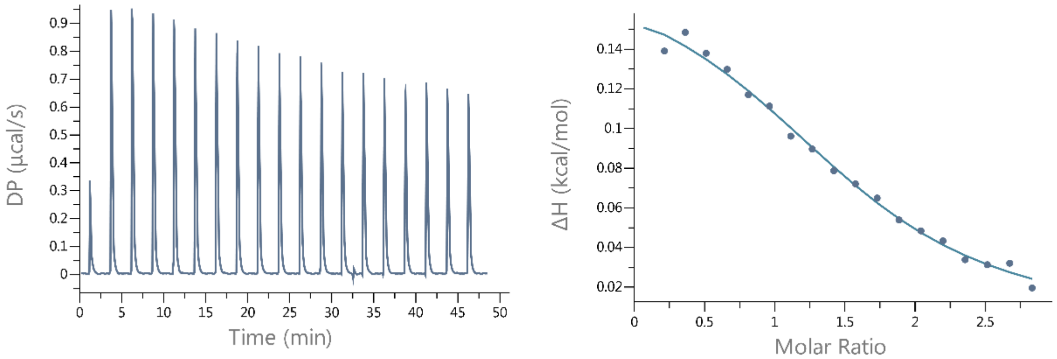

H-L-Lys-OMe x 2HCl to E-1 (PBS):

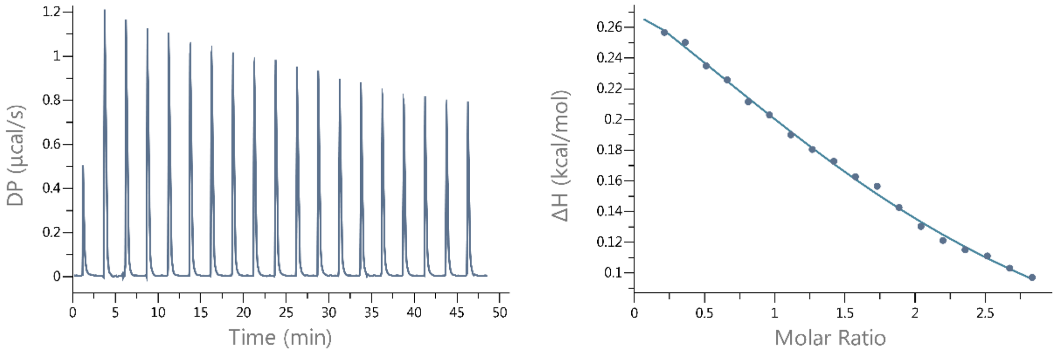

H-L-Lys-OMe x 2HCl to Z-2 (PBS):

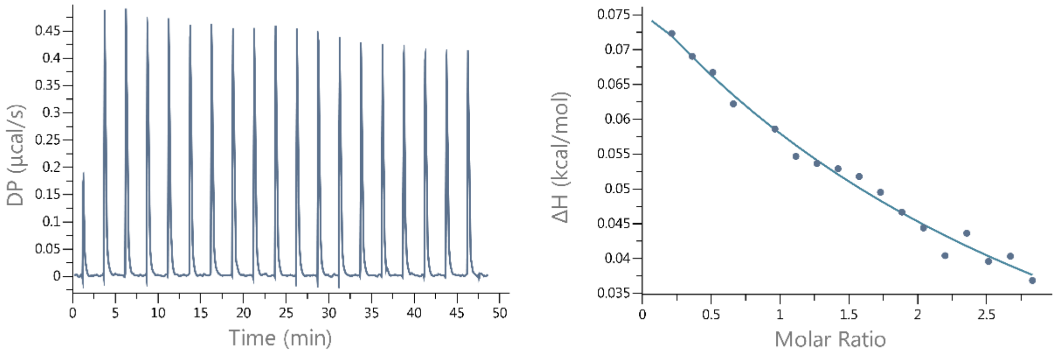

H-L-Lys-OMe x 2HCl to E-2 (PBS):

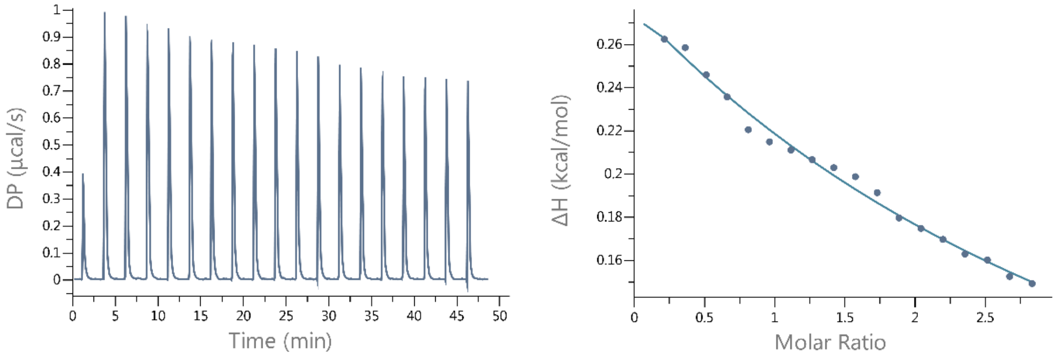

H-L-Arg-OMe x 2HCl to Z-1 (PBS):

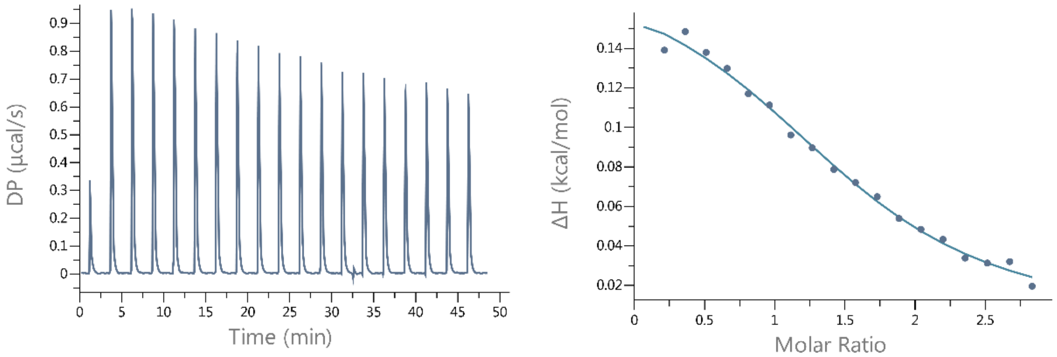

H-L-Arg-OMe x 2HCl to E-1 (PBS):

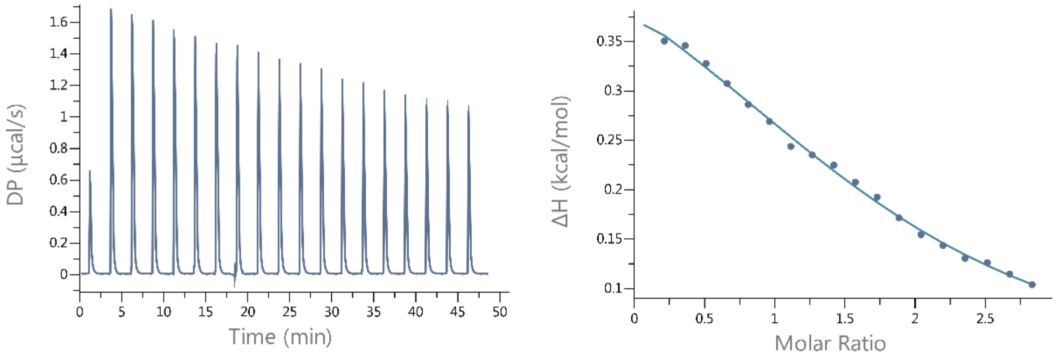

H-L-Arg-OMe x 2HCl to Z-2 (PBS):

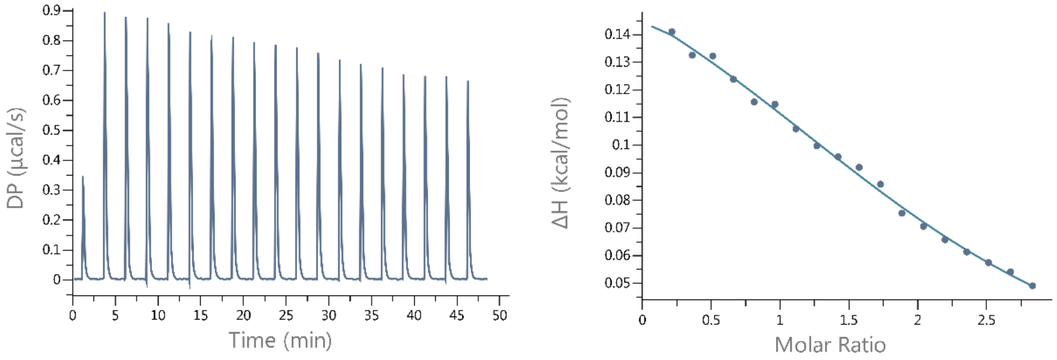

H-L-Arg-OMe x 2HCl to E-2 (PBS):

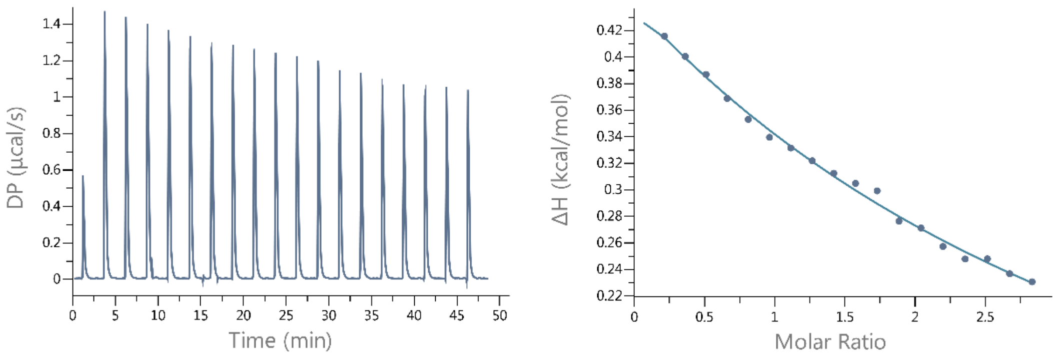

H-L-Orn-OMe x 2HCl to Z-1 (PBS):

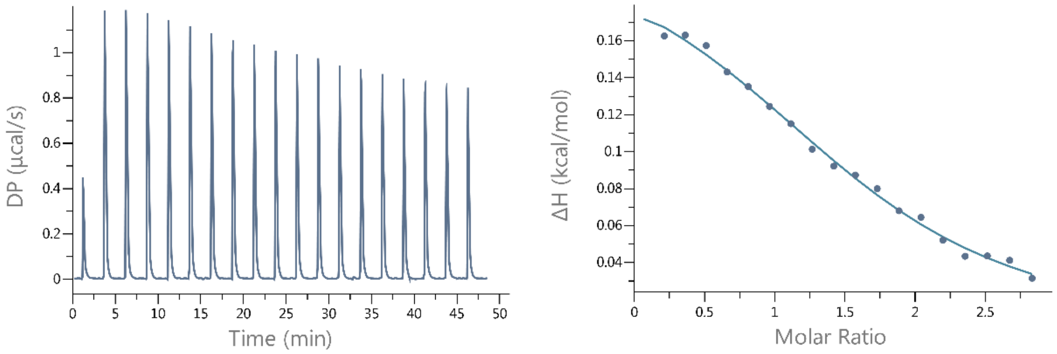

H-L-Orn-OMe x 2HCl to E-1 (PBS):

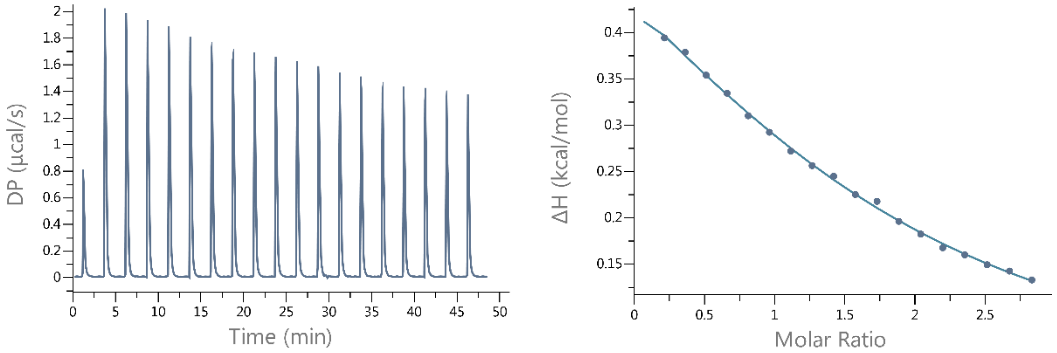

H-L-Orn-OMe x 2HCl to Z-2 (PBS):

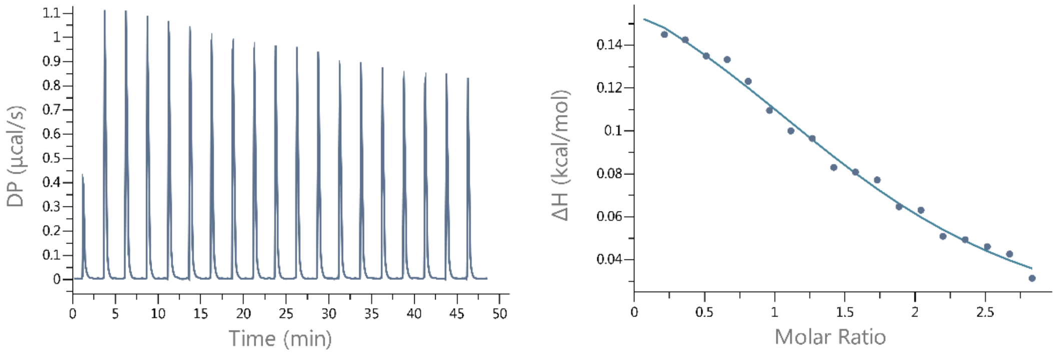

H-L-Orn-OMe x 2HCl to E-2 (PBS):

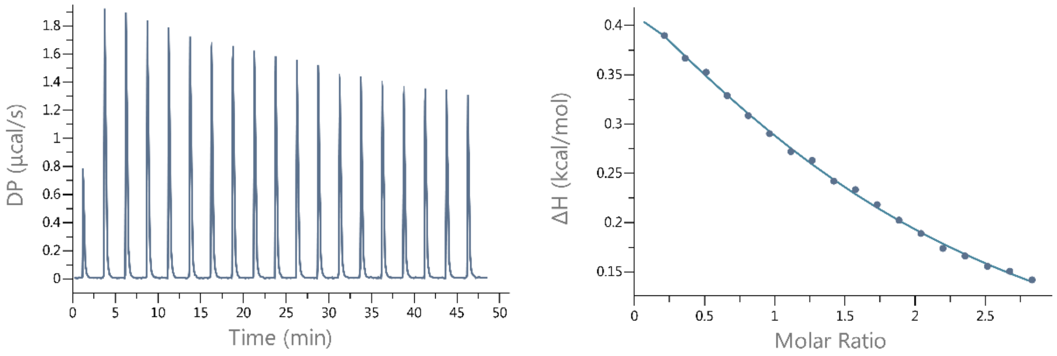

8. Supplementary references

1. L. Albert, A. Penalver, N. Djokovic, L. Werel, M. Hoffarth, D. Ruzic, J. Xu, L.-O. Essen, K. Nikolic, Y. Dou, O. Vázquez, *ChemBioChem* 2019, **20**, 1417–1429.

2. U. Megerle, R. Lechner, B. König, E. Riedle, *Photochem. Photobiol. Sci.* 2010, **9**, 1400–1406.

3. C. Bannwarth, S. Ehlert, S. Grimme, *J. Chem. Theory Comput.* 2019, **15**, 1652–1671.

4. C. Bannwarth, E. Caldeweyher, S. Ehlert, A. Hansen, P. Pracht, J. Seibert, S. Spicher, *Wiley Interdiscip. Rev.: Comput. Mol. Sci.* 2021, **11**.

5. S. Grimme, *J. Chem. Theory Comput.* 2019, **15**, 2847–2862.

6. P. Pracht, F. Bohle, S. Grimme, *Phys. Chem. Chem. Phys.* 2020, **22**, 7169–7192.

7. S. Ehlert, M. Stahn, S. Spicher, S. Grimme *J. Chem. Theory Comput.* 2021, **17**, 4250–4261.

8. S. Grimme, A. Hansen, S. Ehlert, J.-M. Mewes, *J. Chem. Phys.* 2021, **154**, 064103.

9. F. Weigend, R. Ahlrichs, *Phys. Chem. Chem. Phys.* 2005, **7**, 3297–3305.

10. V. Barone, M. Cossi, *J. Phys. Chem. A* 1998, **102**, 1995–2001.

11. S. Grimme, F. Bohle, A. Hansen, P. Pracht, S. Spicher, M. Stahn, *J. Phys. Chem.* 2021, **125**, 4039–4054.

12. F. Neese, F. Wennmohs, U. Becker, C. Riplinger, *J. Chem. Phys.* 2020, **152**, 224108.
