# Supplementary material for: Glucose-derived receptors for photo-controlled binding of amino acid esters in water
Source: Commun Chem. 2025 Feb 19;8:50. doi: 10.1038/s42004-025-01445-x (PMC11840139; doi:10.1038/s42004-025-01445-x)
Supplement: Supplementary file 2 — Description of Additional Supplementary Files [file 42004_2025_1445_MOESM2_ESM.pdf]

Description of Additional Supplementary Files

File name: Supplementary Data 1

Description: NMR spectra of all synthesized compounds

File name: Supplementary Data 2

Description: The conformer-rotamer ensemble sorting settings employed with CENSO, cartesian coordinates of and information on the ensembles.
