# Supplementary material for: Glucose-derived receptors for photo-controlled binding of amino acid esters in water
Source: Commun Chem. 2025 Feb 19;8:50. doi: 10.1038/s42004-025-01445-x (PMC11840139; doi:10.1038/s42004-025-01445-x)

4,6-*O*-isopropylidene-phenyl-β-*D*-glucopyranoside - ^{1}H NMR spectrum (400 MHz, CD_3OD)

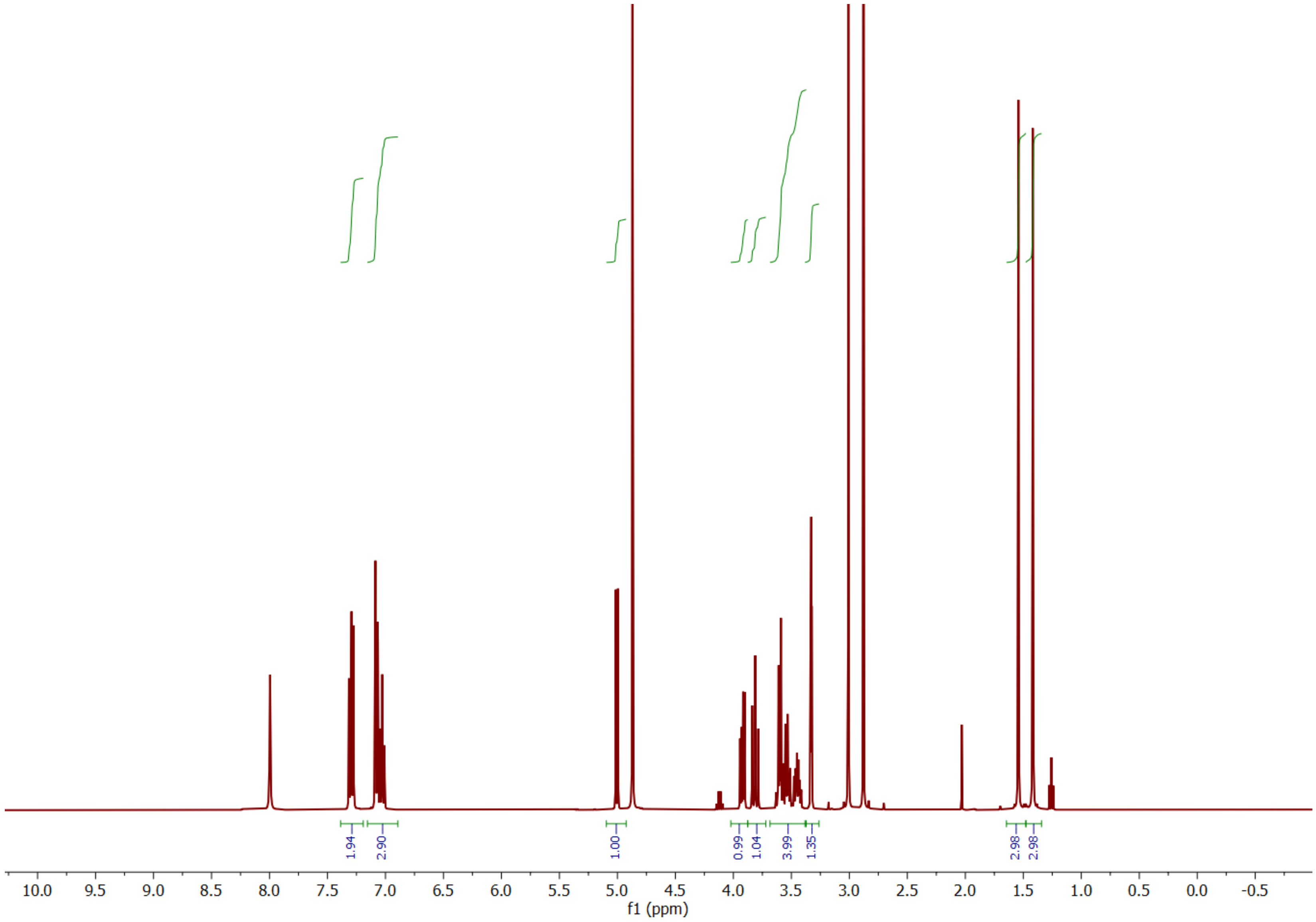

4,6-*O*-isopropylidene-phenyl-β-*D*-glucopyranoside – ^{13}C NMR spectrum (101 MHz, CD_3OD)

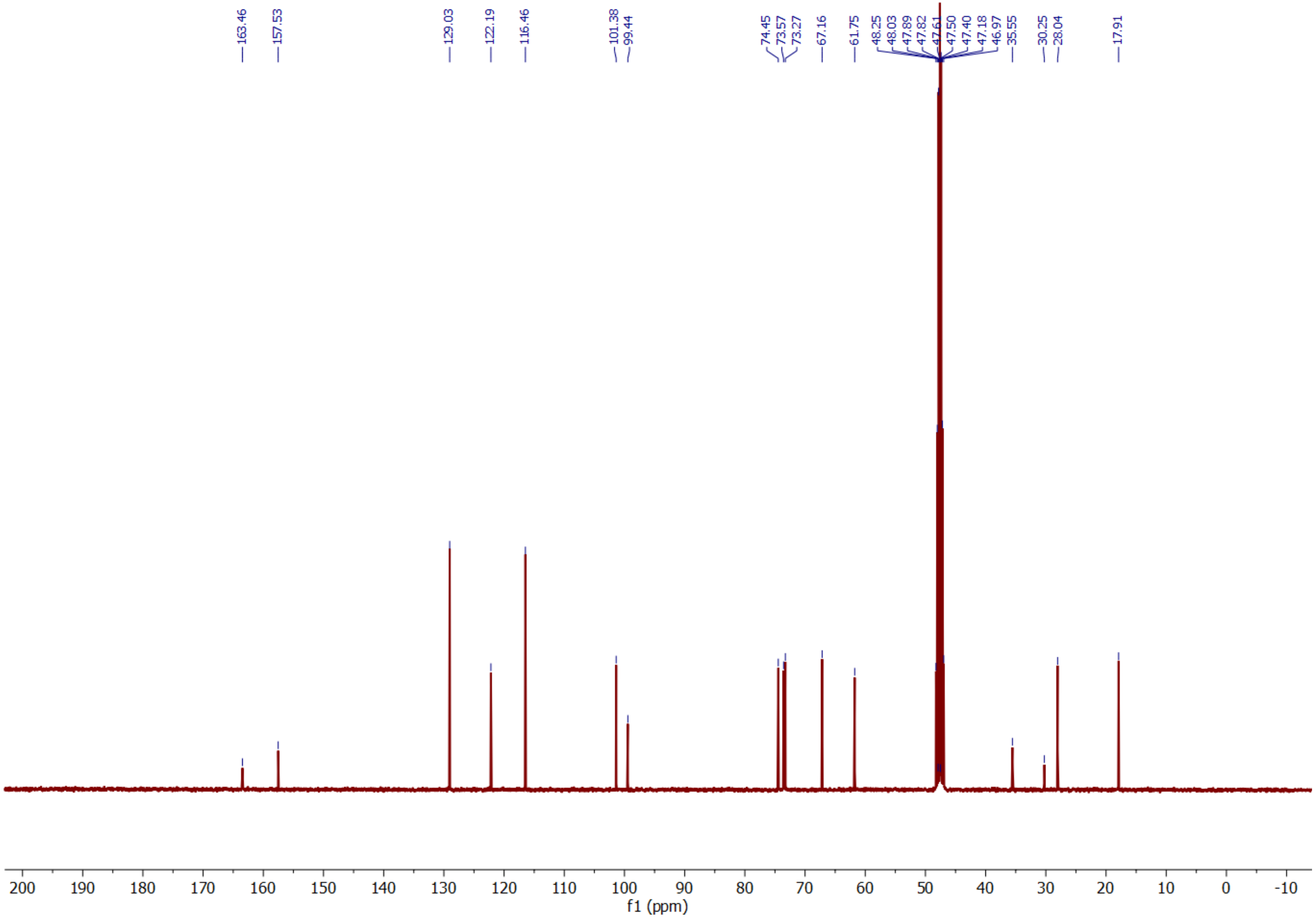

3 - ^{1}H NMR spectrum (400 MHz, $CDCl_3$)

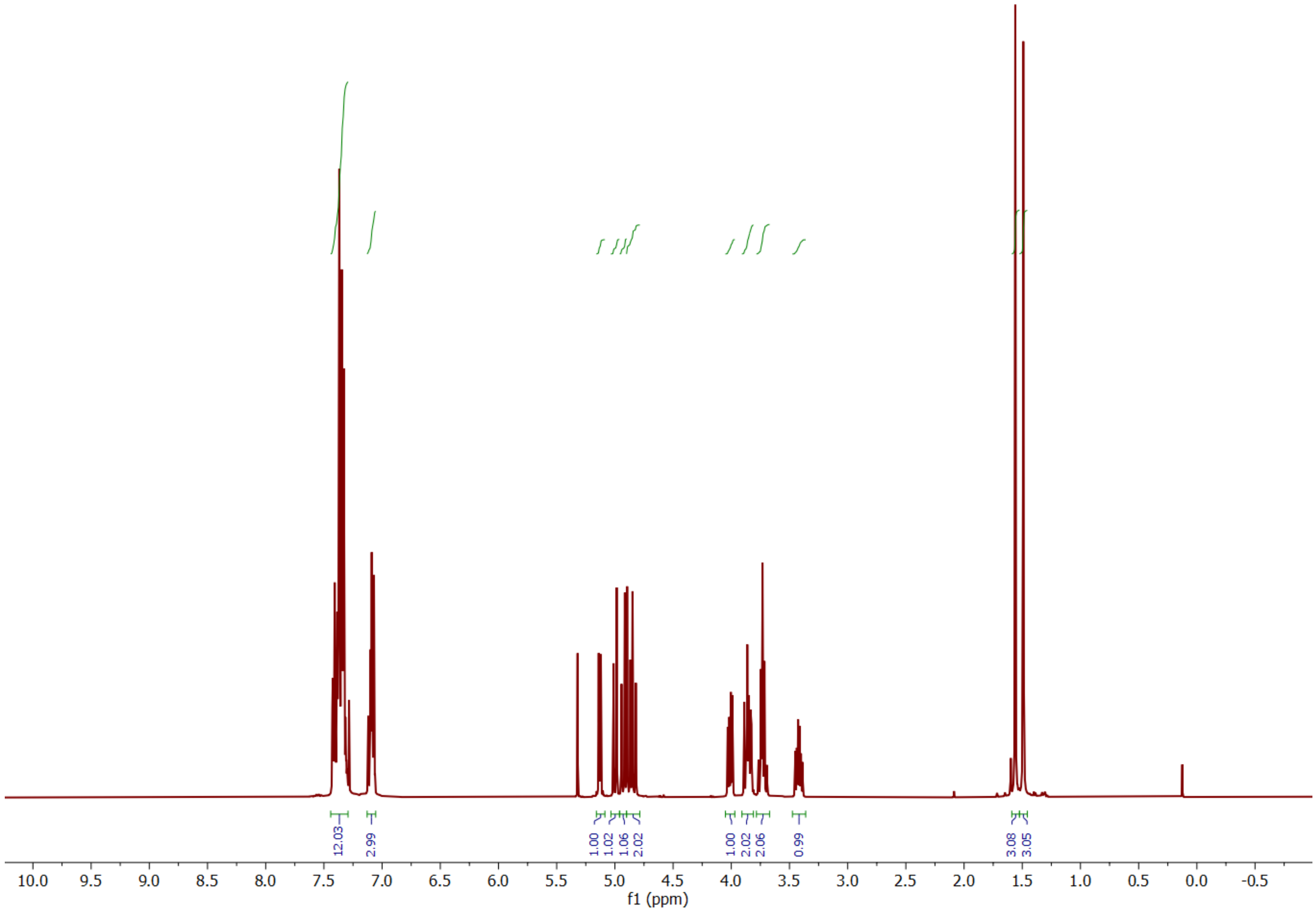

3 – ^{13}C NMR spectrum (101 MHz, $CDCl_3$)

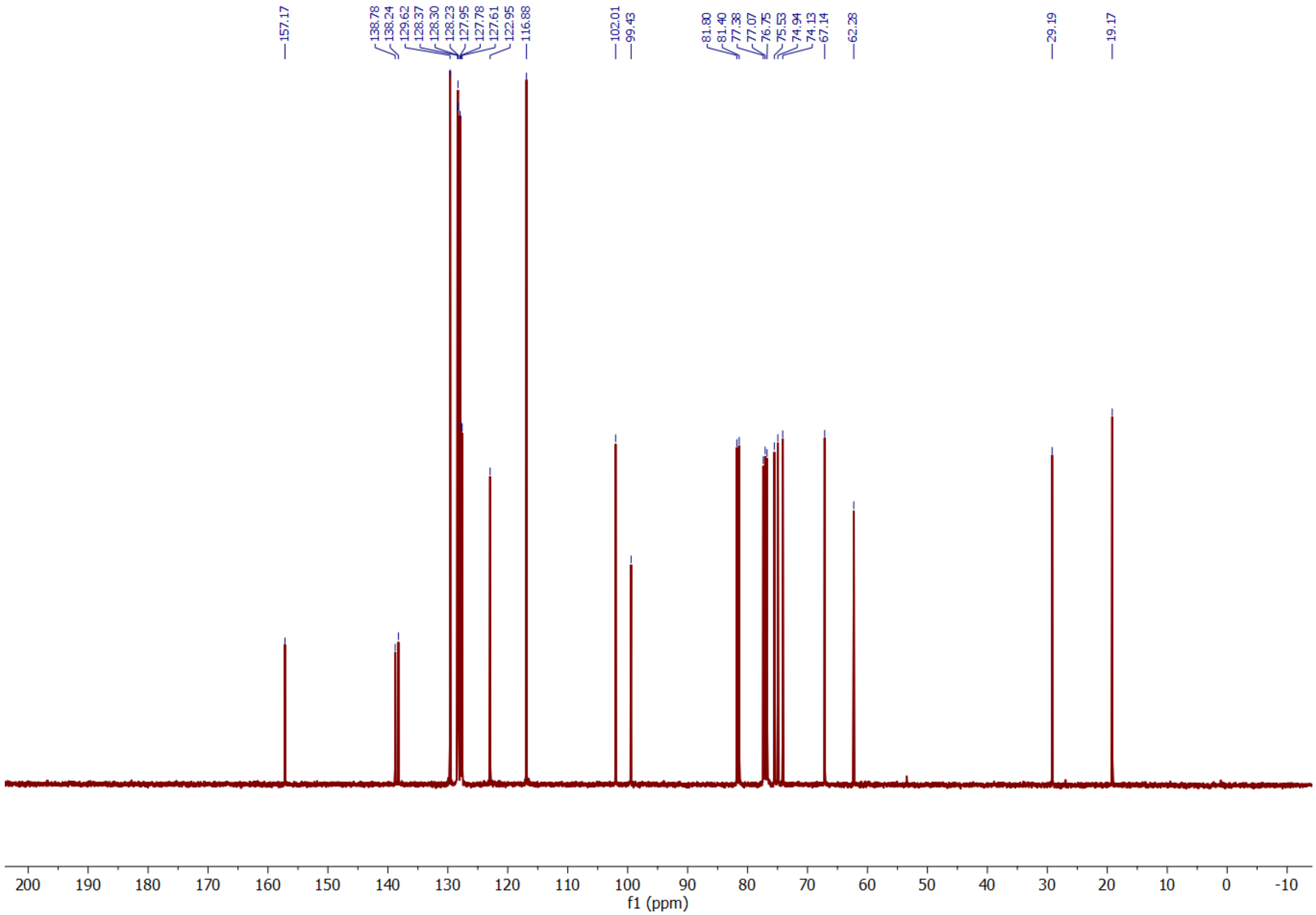

4 - ^{1}H NMR spectrum (400 MHz, $CDCl_3$)

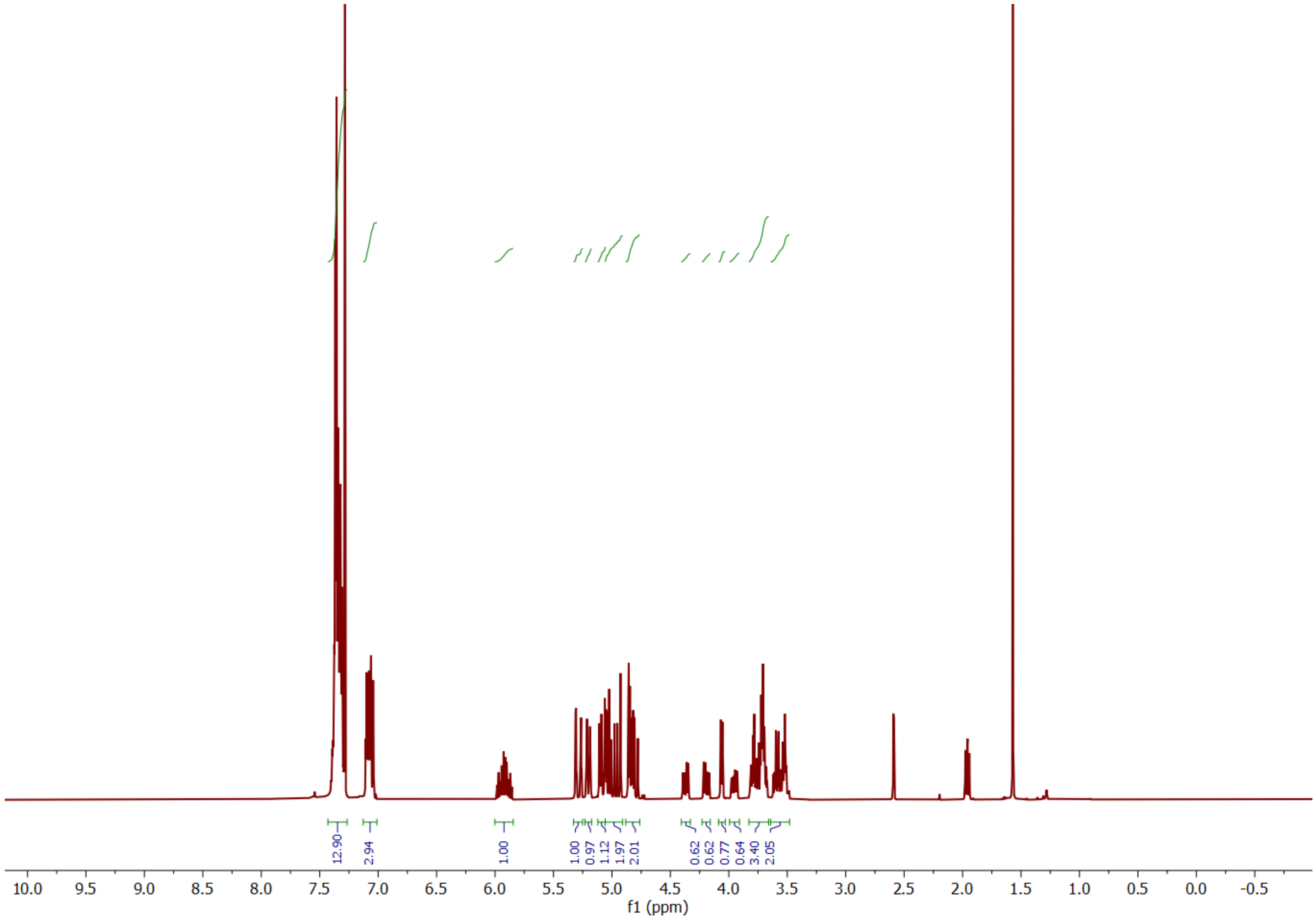

4 – ^{13}C NMR spectrum (101 MHz, $CDCl_3$)

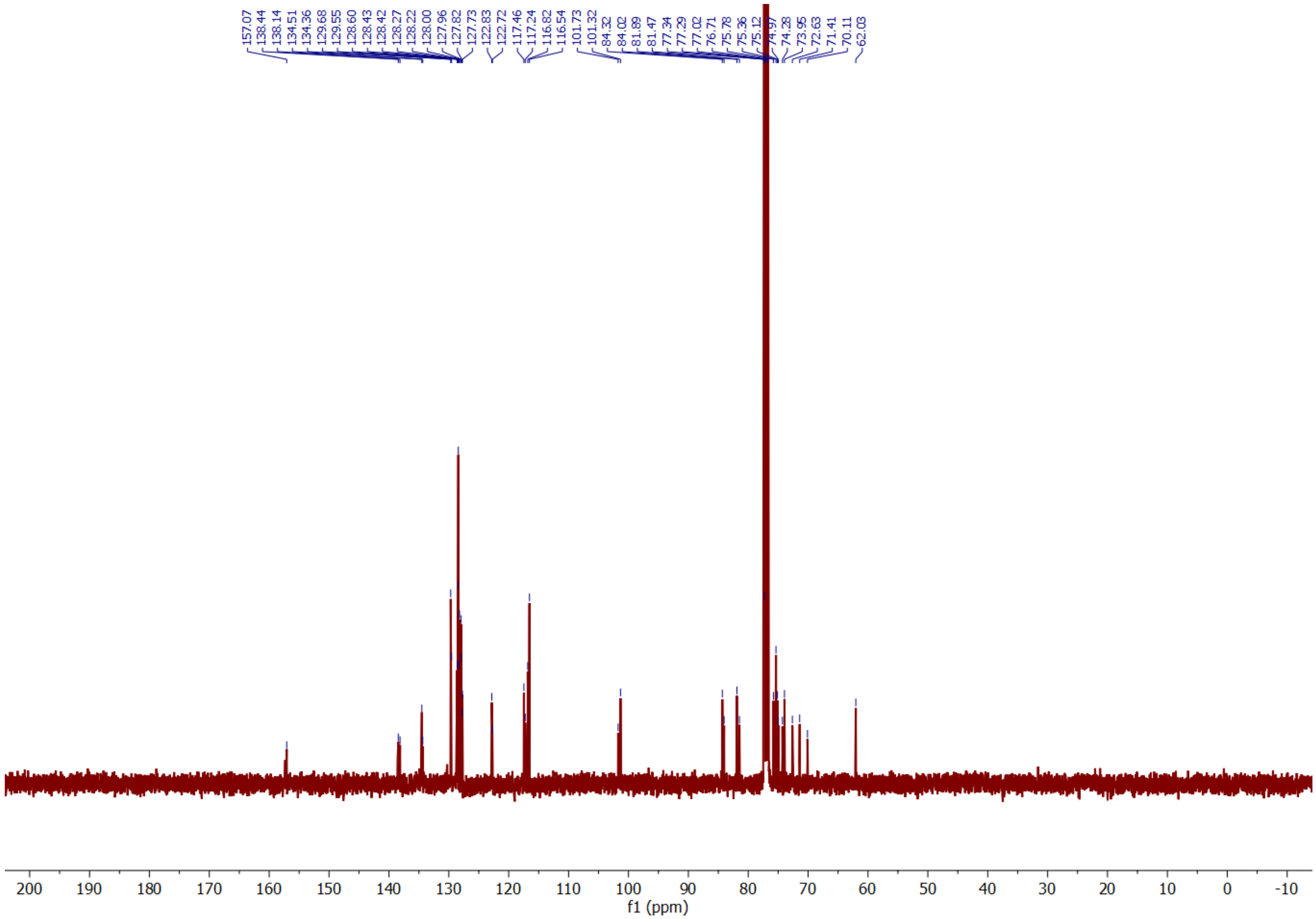

4-*O*-allyl-2,3-di-*O*-benzyl-4-*O*-(2-(2-chloroethoxy)ethoxy)-phenyl-β-*D*-glucopyranoside - ^{1}H NMR spectrum (400 MHz, $CDCl_3$)

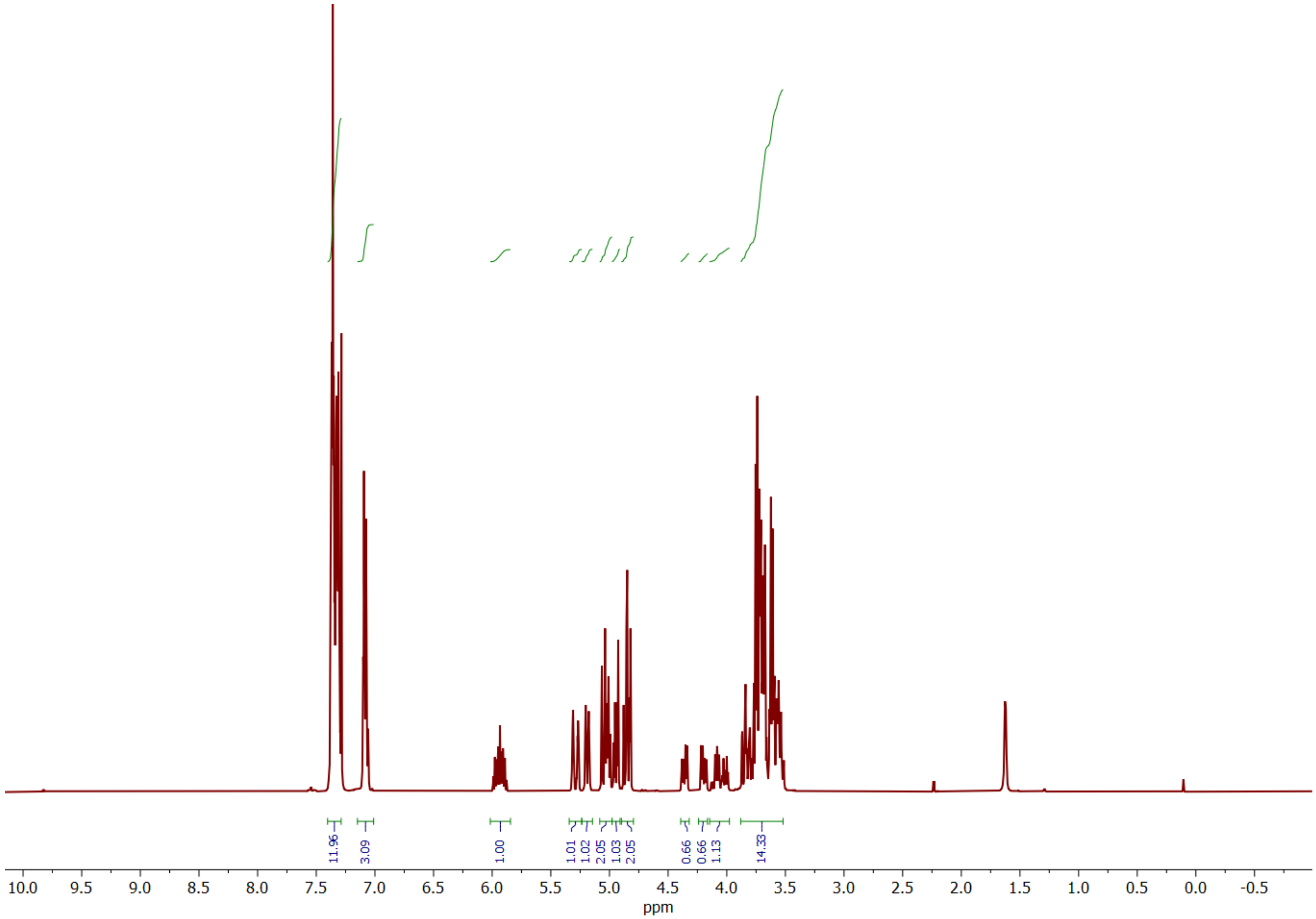

4-*O*-allyl-2,3-di-*O*-benzyl-4-*O*-(2-(2-chloroethoxy)ethoxy)-phenyl-β-*D*-glucopyranoside – ^{13}C NMR spectrum (101 MHz, $CDCl_3$)

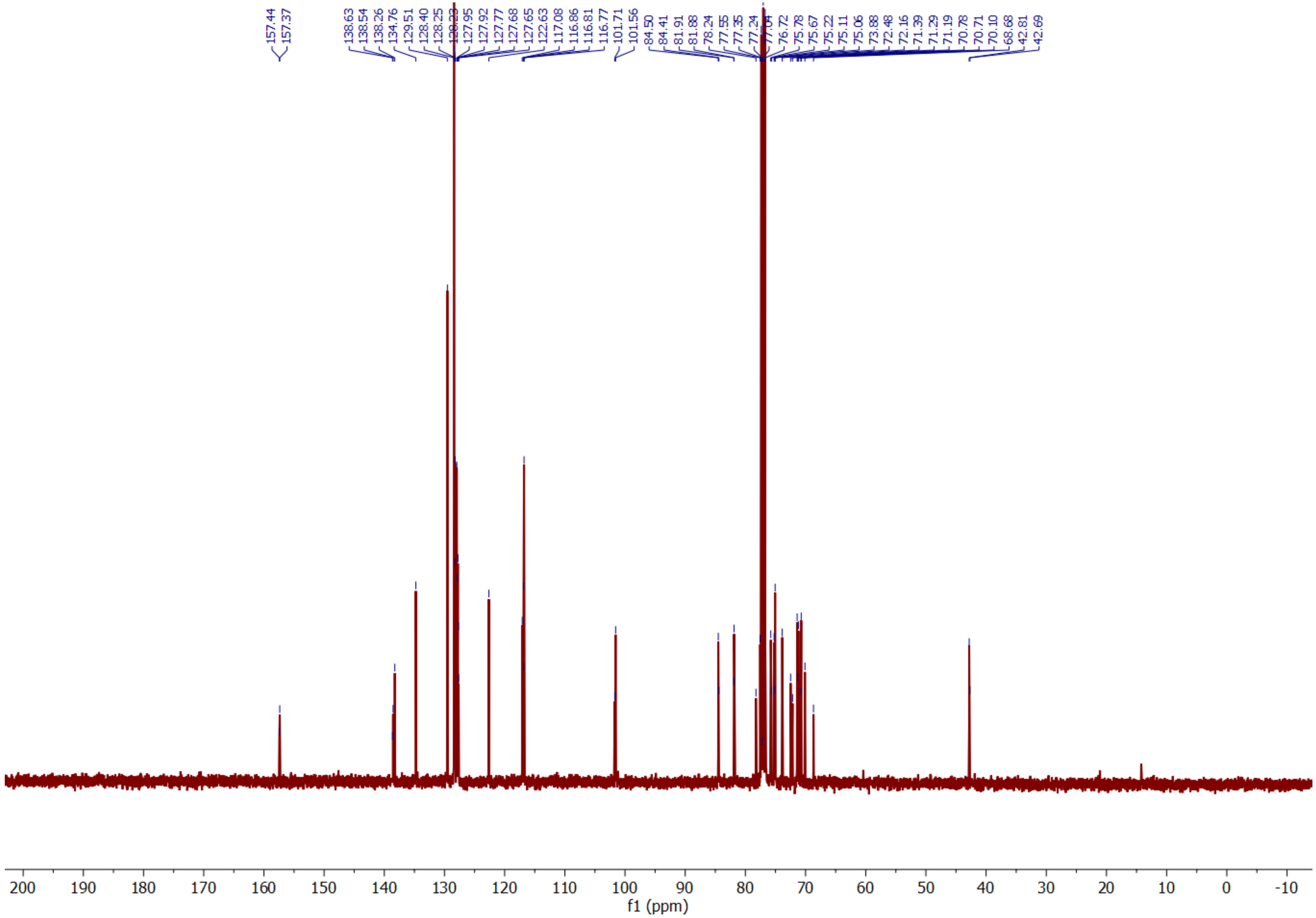

5 - ^{1}H NMR spectrum (400 MHz, $CDCl_3$)

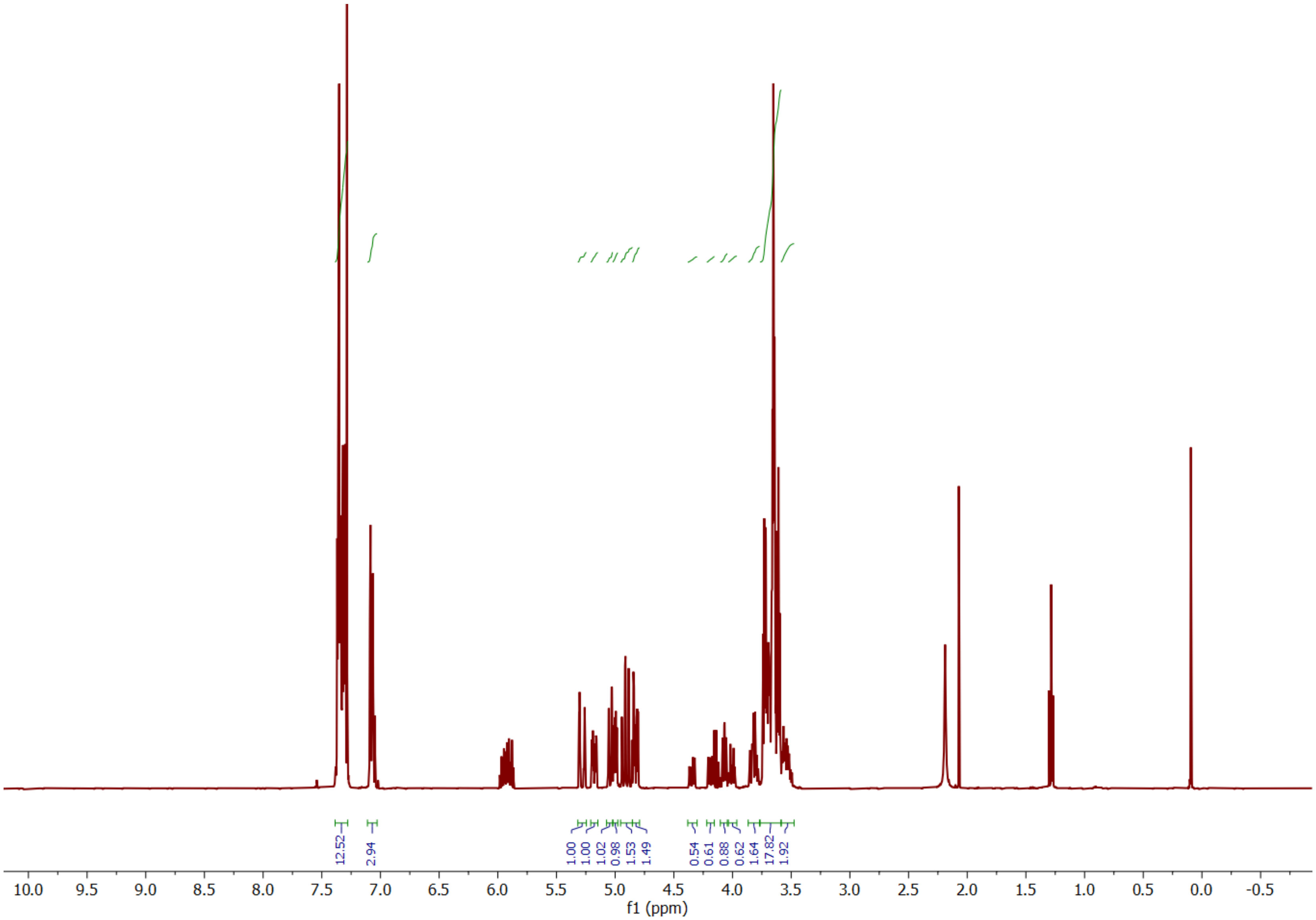

4-*O*-allyl-2,3-di-*O*-benzyl-glucose crown ether – ^{1}H NMR spectrum (400 MHz, $CDCl_3$)

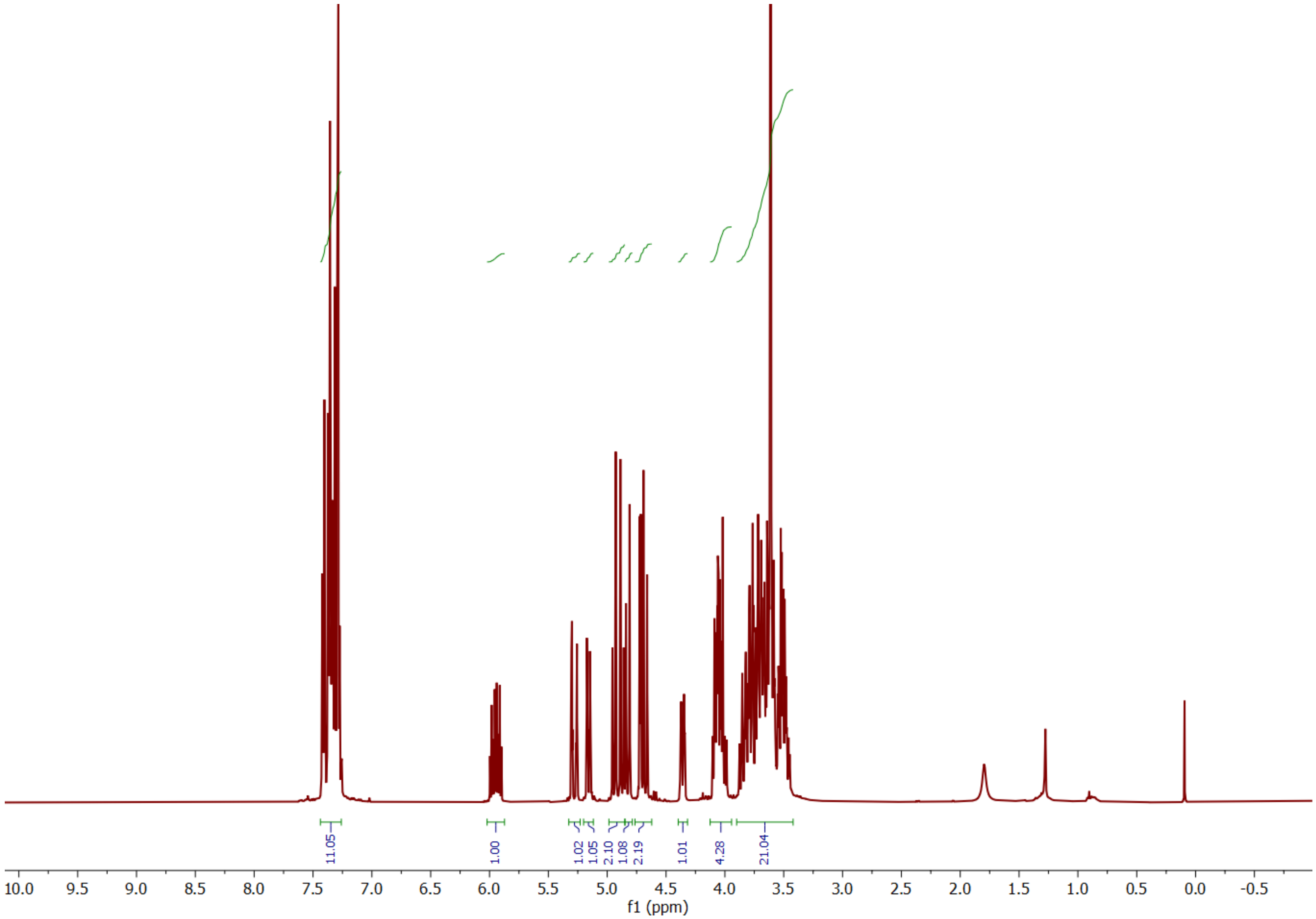

4-*O*-allyl-2,3-di-*O*-benzyl-glucose crown ether – ^{13}C NMR spectrum (101 MHz, $CDCl_3$)

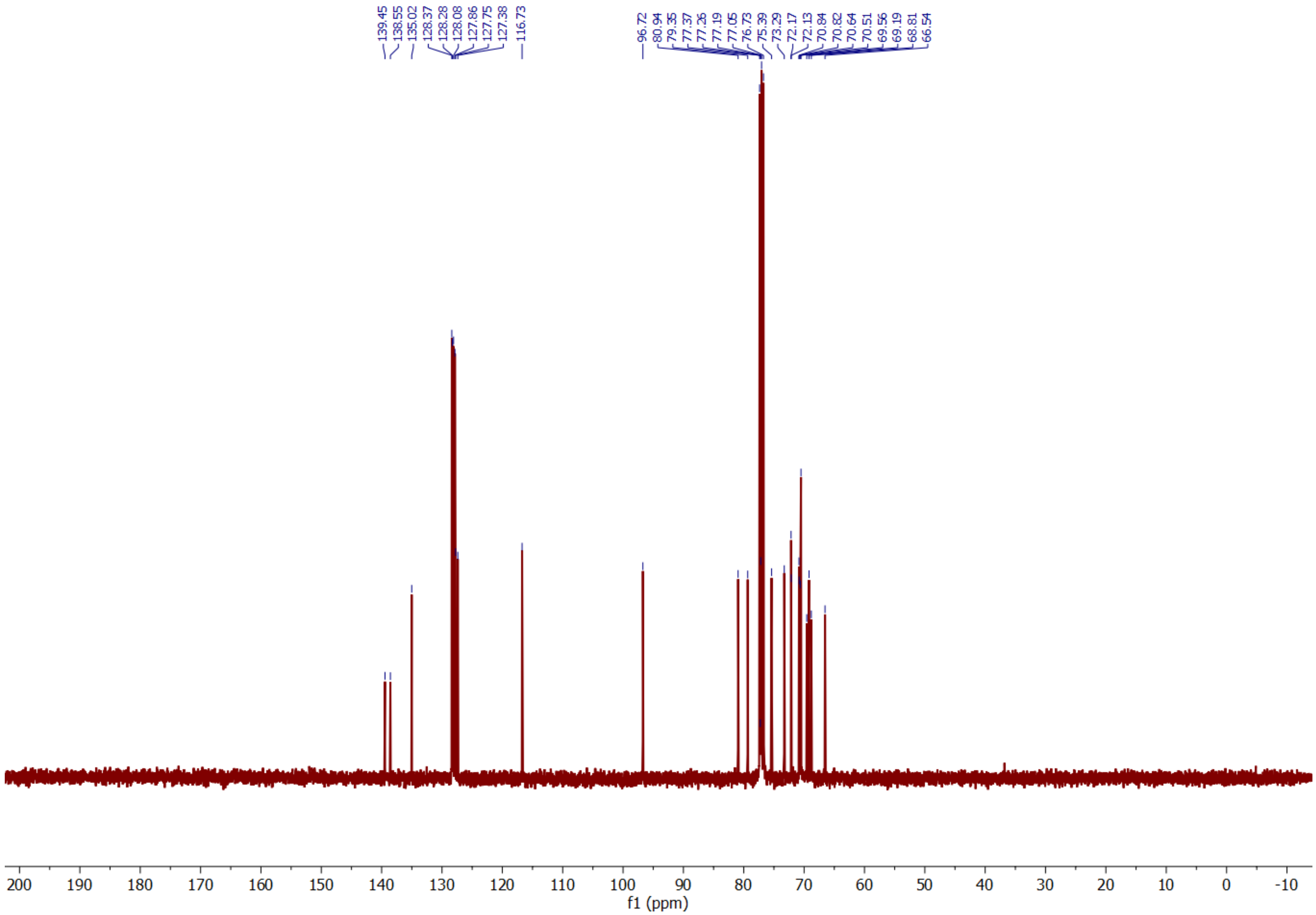

6 - ^{1}H NMR spectrum (400 MHz, $CDCl_3$)

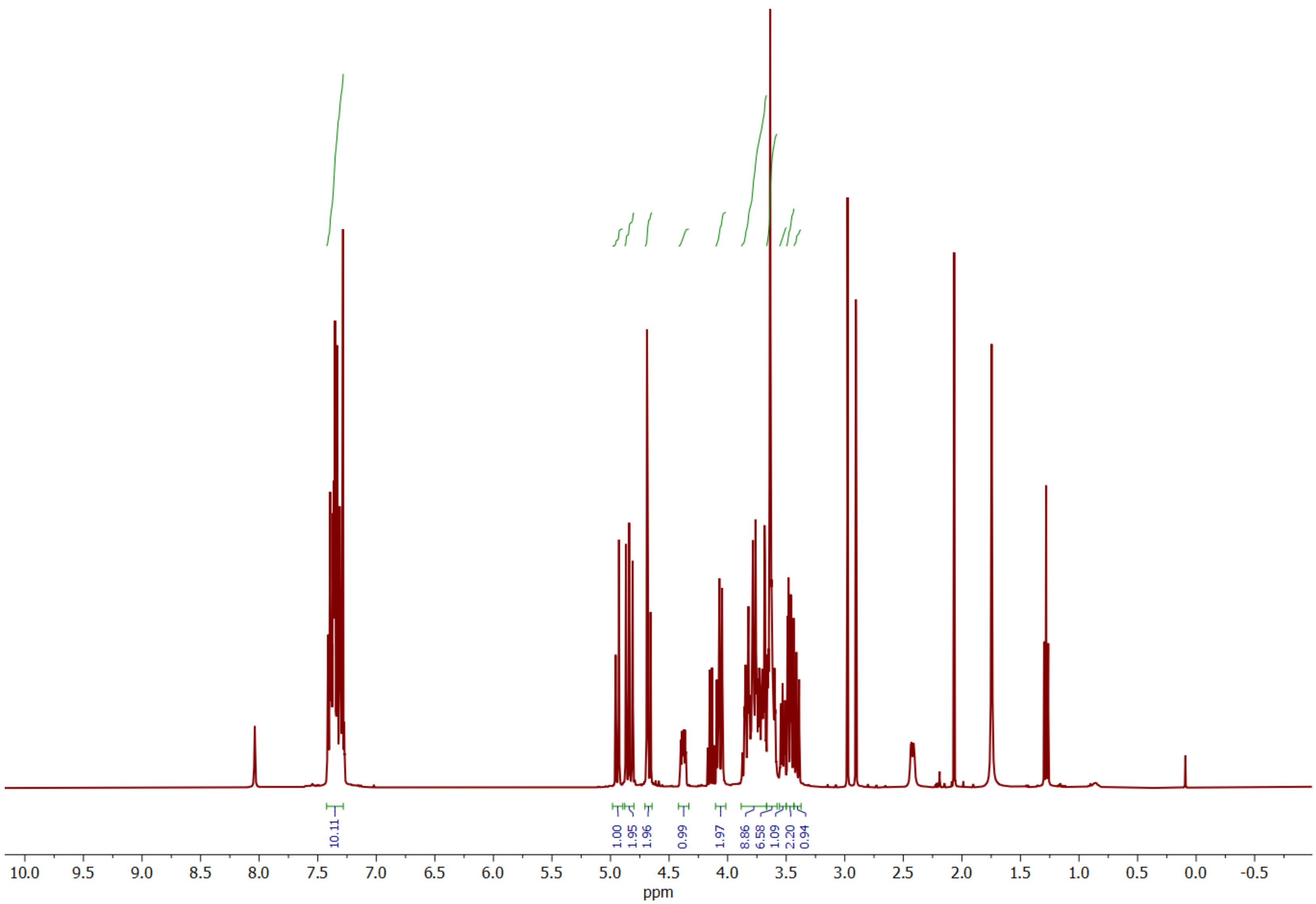

6 – ^{13}C NMR spectrum (101 MHz, $CDCl_3$)

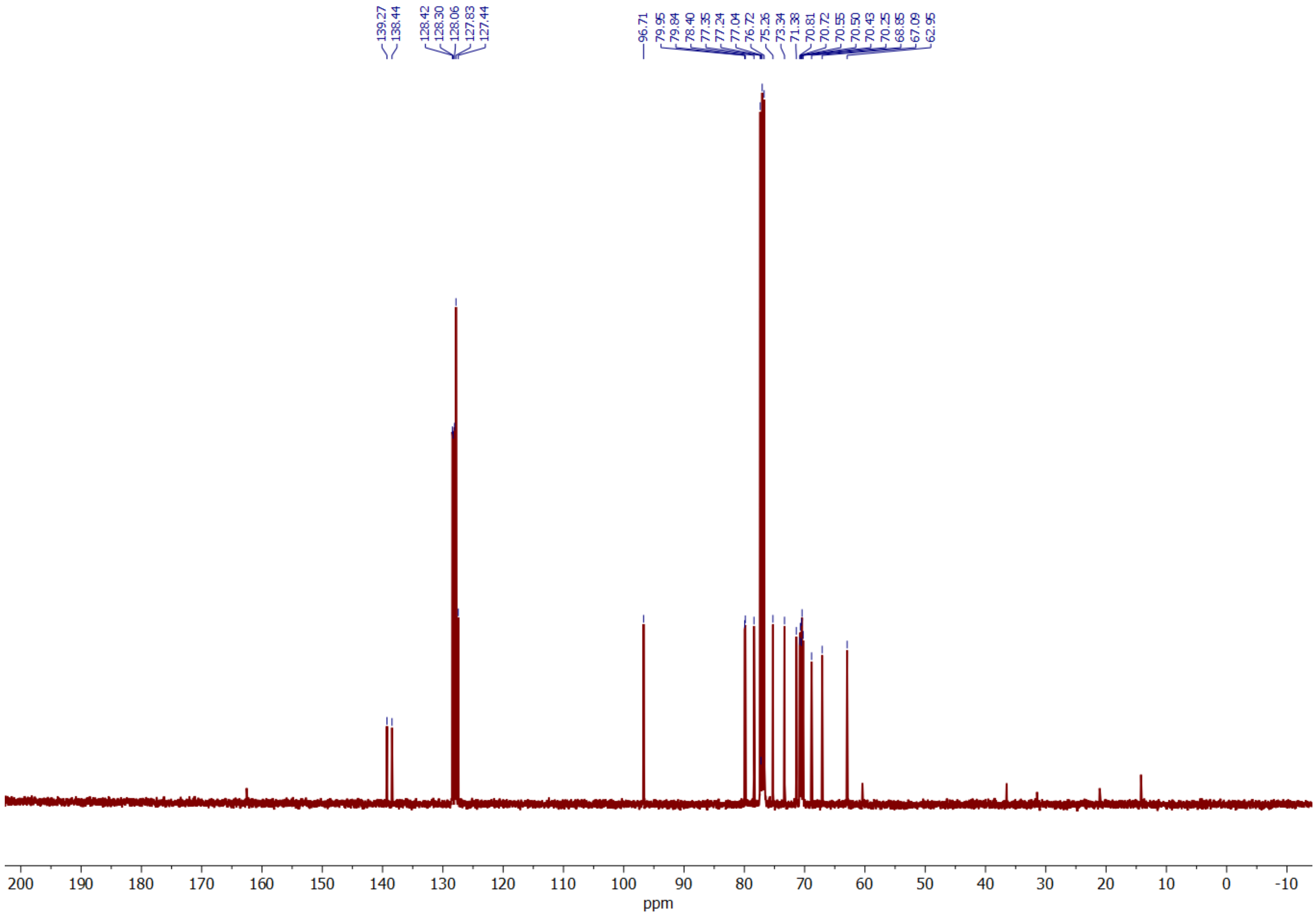

Bn-protected receptor 1 - ^{1}H NMR spectrum (400 MHz, $CDCl_3$)

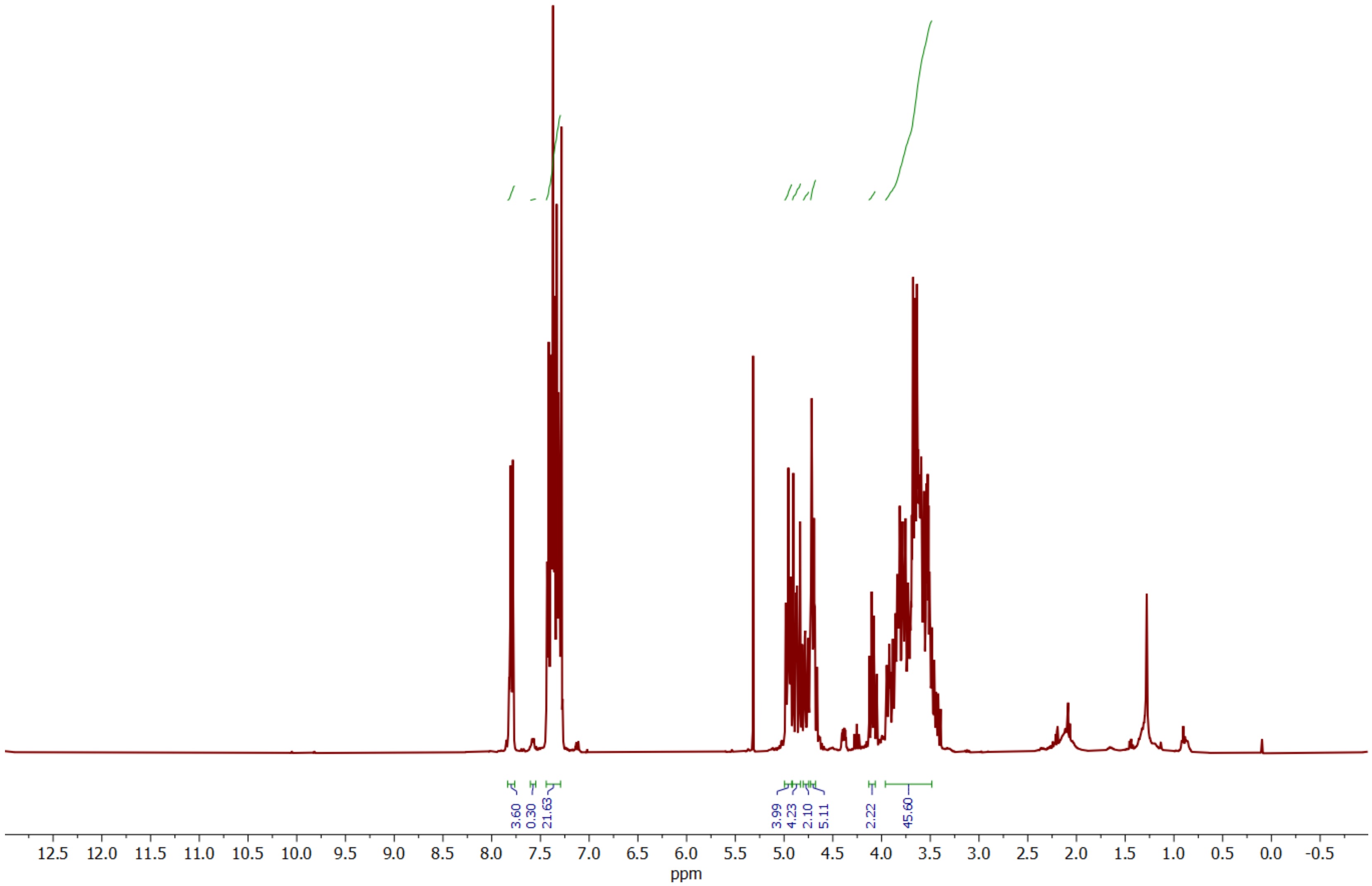

Bn-protected receptor 1 – ^{13}C NMR spectrum (101 MHz, $CDCl_3$)

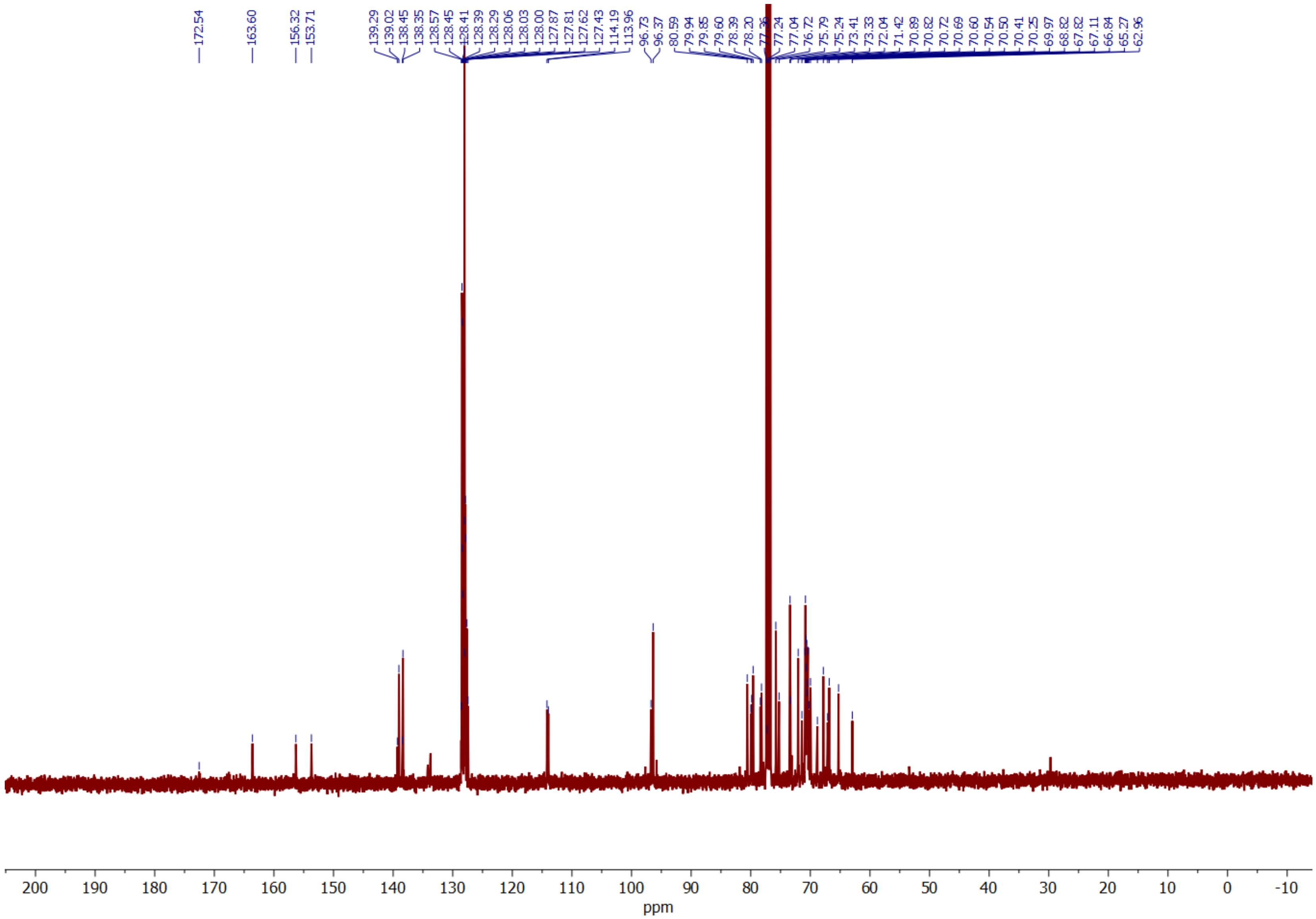

Bn-protected receptor 2 - ^{1}H NMR spectrum (400 MHz, $CDCl_3$)

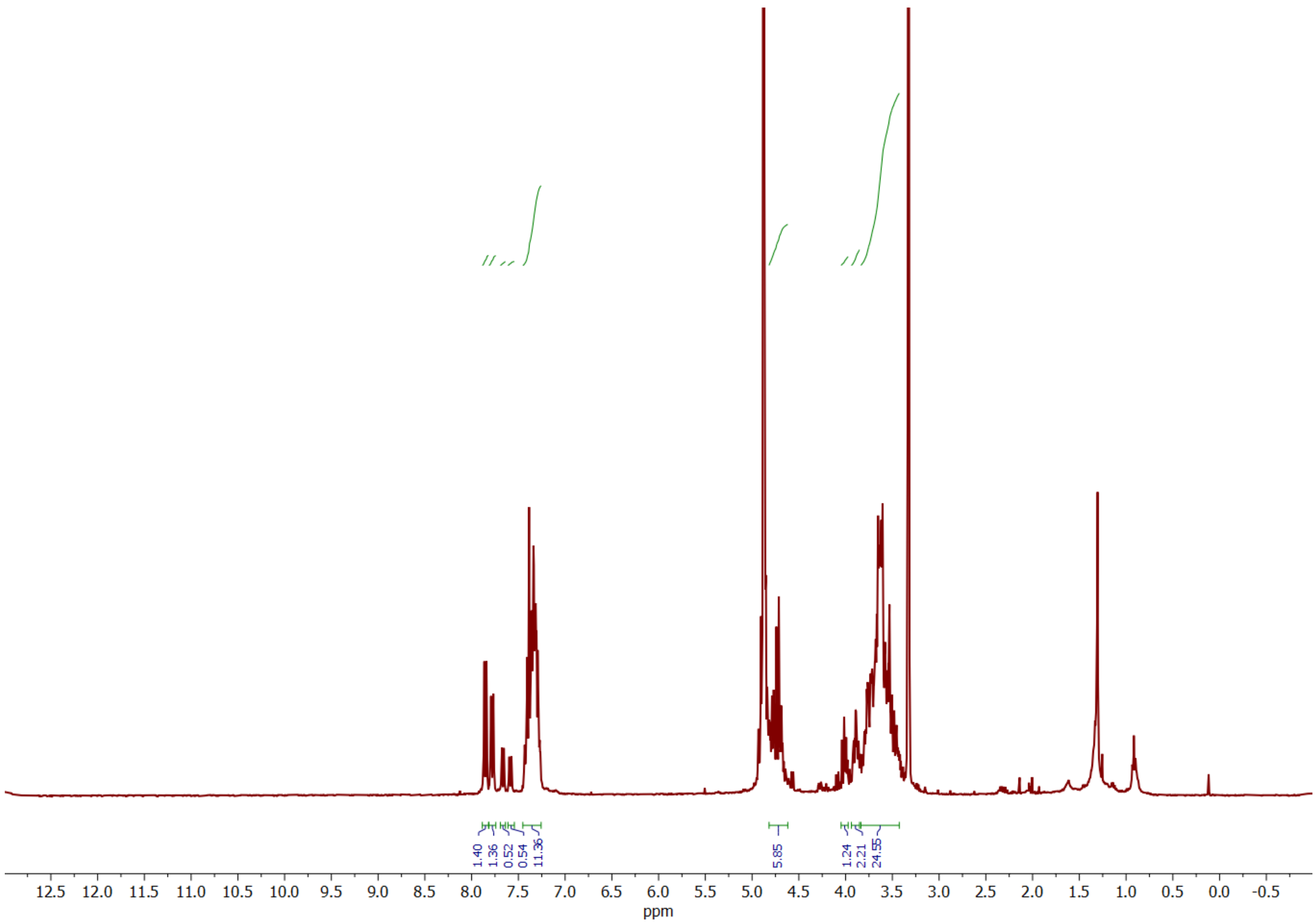

Bn-protected receptor 2 – ^{13}C NMR spectrum (101 MHz, $CDCl_3$)

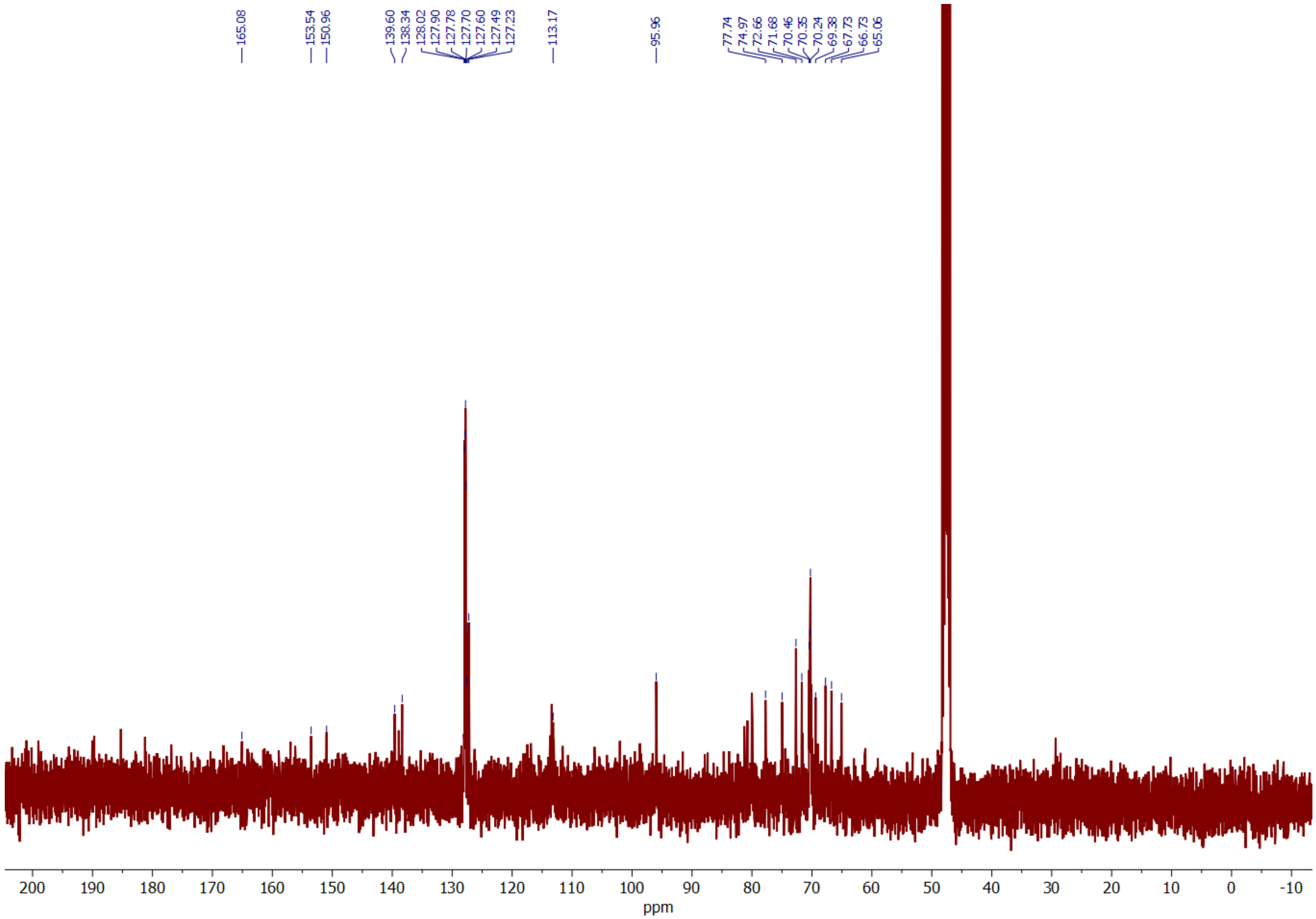

1 - ^{1}H NMR spectrum (400 MHz, CD_3OD)

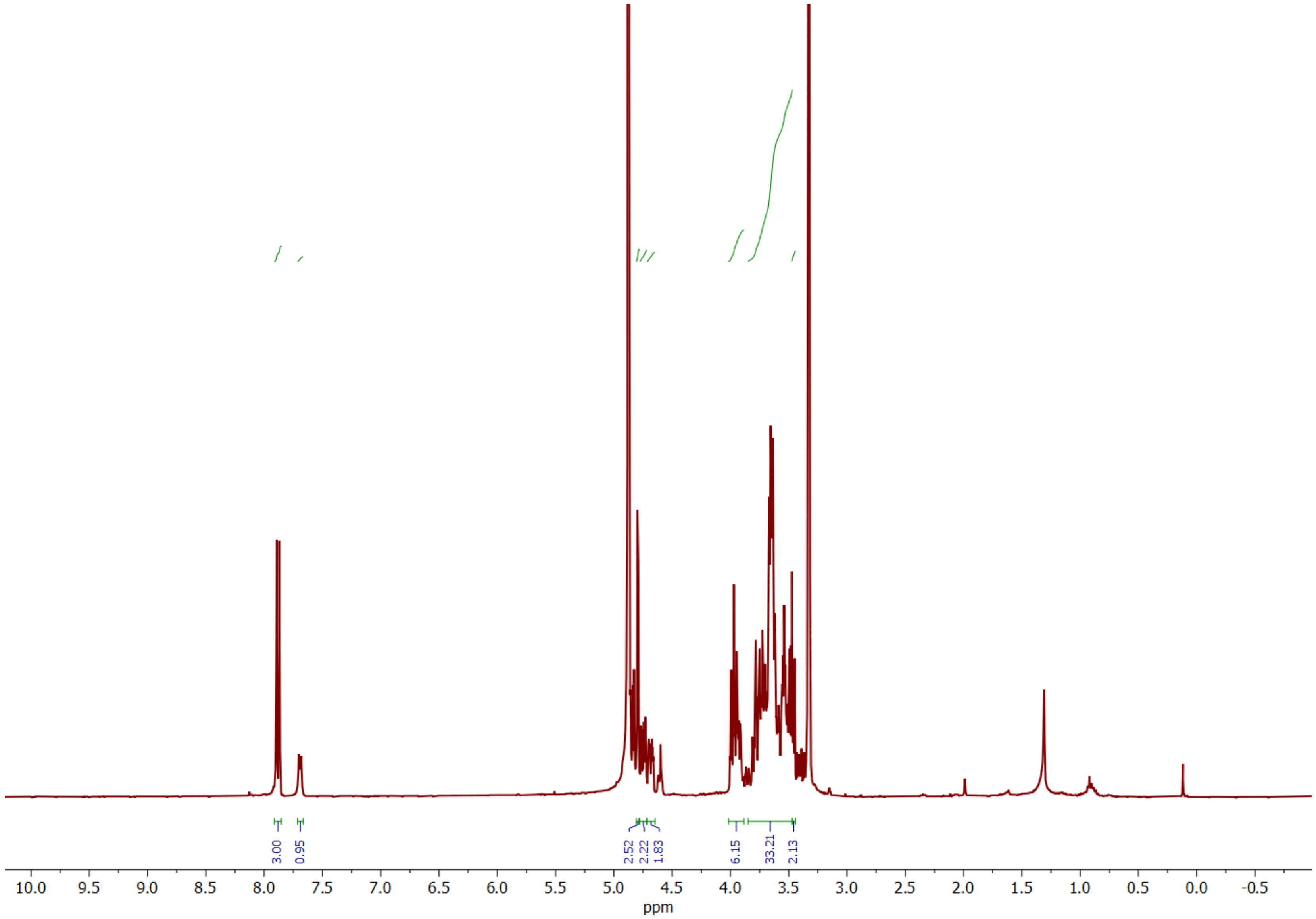

1 – ^{13}C NMR spectrum (101 MHz, CD_3OD)

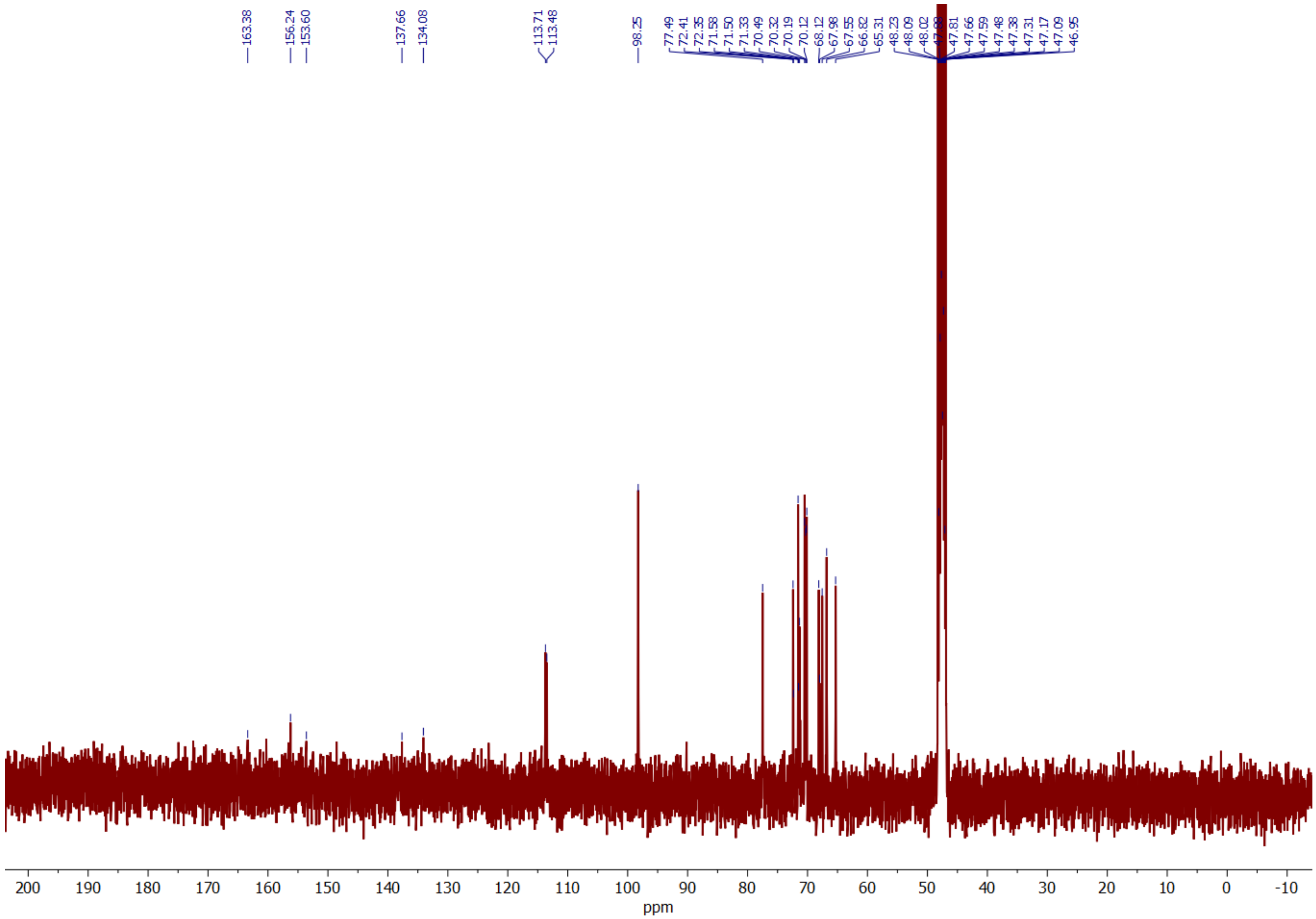

1 – ^{19}F NMR spectrum (377 MHz, CD_3OD)

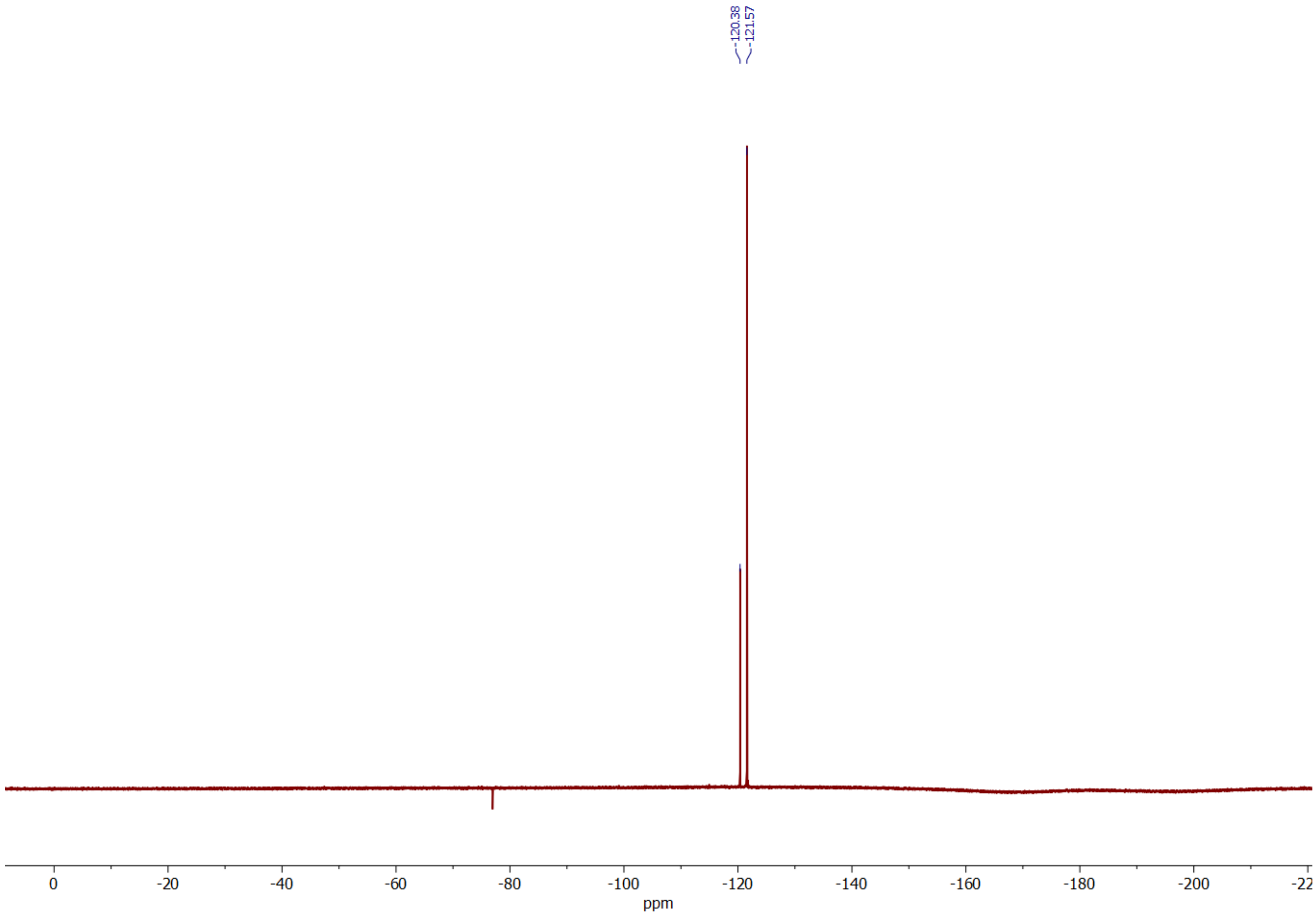

2 - ^{1}H NMR spectrum (400 MHz, CD_3OD)

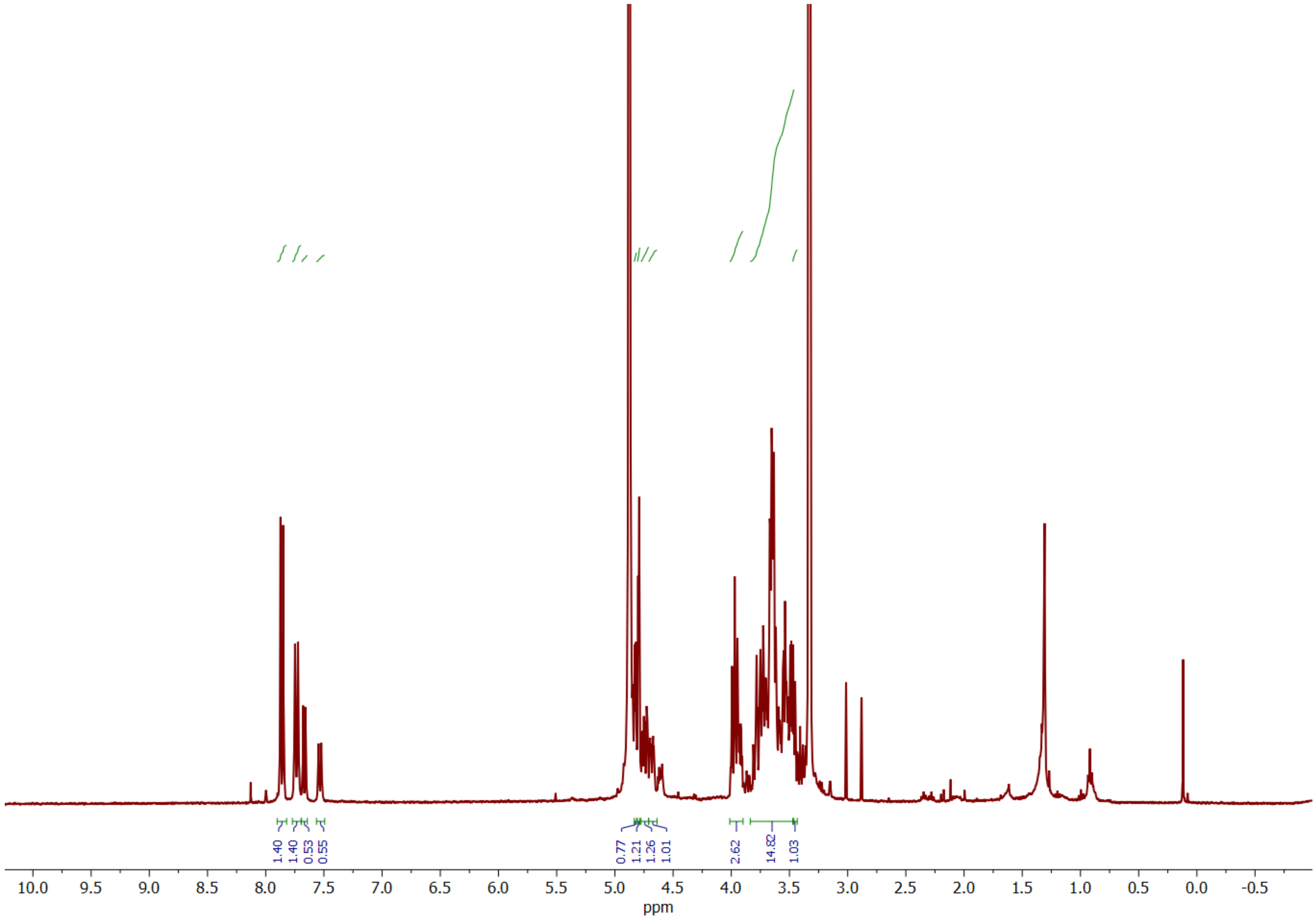

2 – ^{19}F NMR spectrum (377 MHz, CD_3OD)

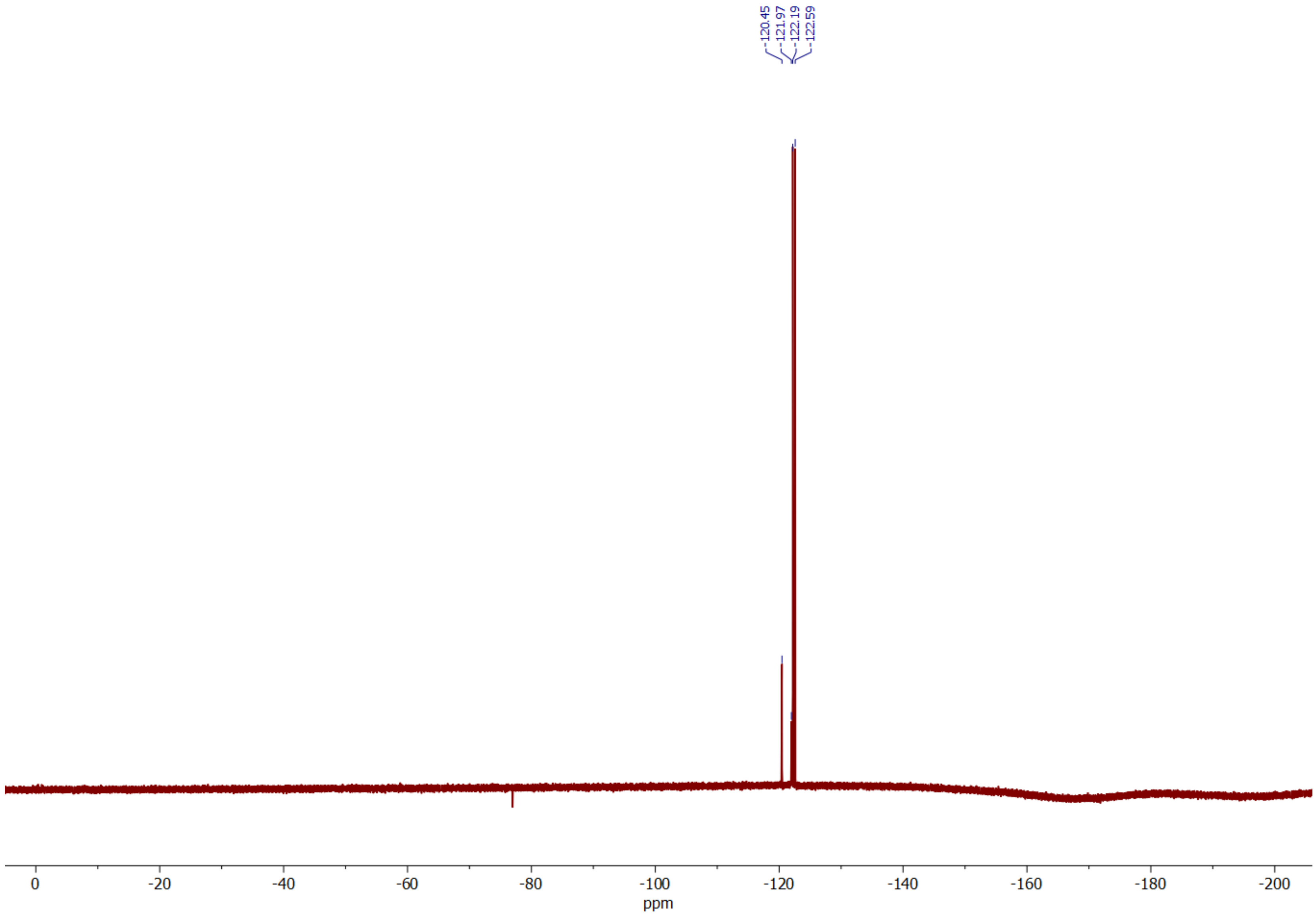

Supplement: Supplementary file 3 — Supplementary Data 1 [file 42004_2025_1445_MOESM3_ESM.pdf]
